# Soil Metabolome Impacts the Formation of the Eco-corona and Adsorption Processes on Microplastic Surfaces

**DOI:** 10.1021/acs.est.3c01877

**Published:** 2023-05-17

**Authors:** Shi Yao, Xiaona Li, Tao Wang, Xin Jiang, Yang Song, Hans Peter H. Arp

**Affiliations:** † CAS Key Laboratory of Soil Environment and Pollution Remediation, Institute of Soil Science, Chinese Academy of Sciences, Nanjing 210008, PR China; ‡ University of Chinese Academy of Sciences, Beijing 100049, PR China; § School of Environmental Science and Engineering, Jiangnan University, Wuxi 225127, PR China; ∥ Institute of Mountain Hazards and Environment, Chinese Academy of Sciences, Chengdu 610299, PR China; ⊥ Norwegian Geotechnical Institute (NGI), P.O. Box 3930, Ullevål Stadion, Oslo N-0806, Norway; # Department of Chemistry, Norwegian University of Science and Technology (NTNU), Trondheim NO-7491, Norway

**Keywords:** eco-corona, polyethylene, sorption, metabolite, phthalate

## Abstract

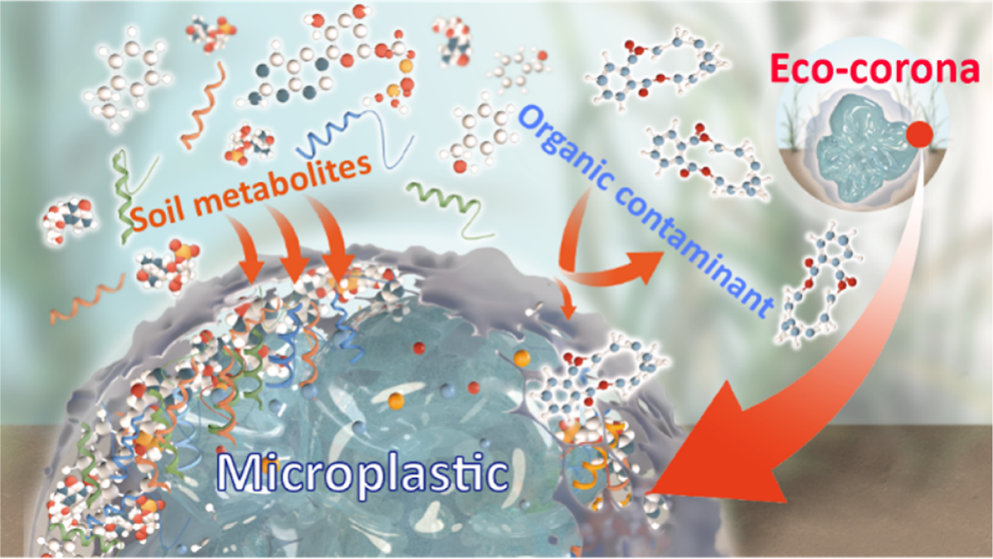

The eco-corona on microplastics refers to the initial layer of biomolecular compounds adsorbed onto the surface after environmental exposure. The formation and composition of the eco-corona in soils have attracted relatively little attention; however, the eco-corona has important implications for the fate and impacts of microplastics and co-occurring chemical contaminants. Here, it was demonstrated that the formation of the eco-corona on polyethylene microplastics exposed to water-extractable soil metabolites (WESMs) occurs quite rapidly via two pathways: direct adsorption of metabolites on microplastics and bridging interactions mediated by macromolecules. The main eco-corona components were common across all soils and microplastics tested and were identified as lipids and lipid-like molecules, phenylpropanoids and polyketides, nucleosides, nucleotides, and their analogues. WESMs were found to reduce the adsorption of co-occurring organic contaminants to microplastics by two pathways: reduced adsorption to the eco-corona surface and co-solubilization in the surrounding water. These impacts from the eco-corona and the soil metabolome should be considered within fate and risk assessments of microplastics and co-occurring contaminants.

## Abstract 2

This study shows that soil metabolites from different soils form an eco-corona of similar composition on microplastics, which acts as a barrier, inhibiting the adsorption of other metabolites and organic contaminants.

## Introduction

Plastic waste has been discharged into the aquatic and terrestrial environments at rates of 25–45 million metric tons annually.^1,2^ Over time, much of the discharged plastics will weather and slowly degrade into microplastics (<5 mm).^3,4^ The terrestrial environment is the main pool of microplastics globally.^5,6^ Several concerns about plastic pollution and microplastic pollution in terrestrial environments have been raised, including the spread of pathogenic microorganisms,^5,7^ impacts on animal growth and reproduction,^8,9^ the restriction of plant growth,^10,11^ human exposure through the food chain,^12−14^ and interference in biogeochemical cycling processes, including carbon, nitrogen, and nutrient cycles.^15,16^ Plastic pollution in both terrestrial and aquatic environments is considered a global threat to environmental and human health.^17^

In addition to littering and poor waste management, other ways plastic can accumulate in soils are through plastic film mulching, application of biosolid compost containing plastic residues, and air deposition.^17,18^ After entering the soil, microplastics inevitably encounter soil metabolomes, a group of low-molecular-weight bio-signaling compounds produced by plant root exudation, microbial and animal metabolism, and the decomposition of soil organic matter.^19^ Soil metabolomes are recognized as the most active biomolecules in soil dissolved organic matter (DOM) and act as key compounds which can pass through soil microbial cells and play vital roles in driving soil biogeochemical cycles.^5,20^ Thus, it is of interest to see which soil metabolites can adsorb and ultimately form a thin layer of biomolecular compounds on the surface of microplastic particles; this layer is often referred to as the “eco-corona”.^21−23^ Such an eco-corona has been observed on nanomaterials in aquatic environments to occur via the interactions of particles and DOM components such as proteins, metabolites, and extracellular polymers. The eco-corona modifies the surface properties of the particles and thereby can significantly influence their aggregation, migration, biodistribution, biofilm formation, and toxic effects.^24−27^ Therefore, elucidating the formation of the eco-corona on microplastics in soil is of great importance for microplastics’ environmental fate and risk assessment. Considering the heterogeneity of soils, diversified soil metabolites may lead to the formation of eco-coronas with varying compositions depending on the soil type.

An additional impact the eco-corona can have is on the sorption of co-occurring contaminants. Microplastics can have contaminants both adsorbed to their surface^28,29^ within or on top of the eco-corona and absorbed inside the plastic matrix itself. In this manner, microplastics can serve as sources, sinks, or vectors of contaminants and thereby impact the bioavailability and fate of contaminants to plants and soil-dwelling organisms.^30,31^ Although the eco-corona itself would not affect the internal absorption capacity of microplastics, it could still affect the adsorption capacity and kinetics on the surface of microplastics, such as by competing for surface sites and/or changing the surface properties of the microplastics. However, little focus has been placed on this issue until the present study.

The objectives of the study were to investigate the formation of the eco-corona on microplastics based on their interactions with different soil metabolomes and to characterize the impact that this eco-corona has on contaminant sorption. Three hypotheses were explored: (i) the adsorptive interactions between microplastics and soil metabolomes cause the eco-corona to form quickly; (ii) this eco-corona is soil-specific due to diversified soil metabolomes; and (iii) once this eco-corona is formed, it will affect the sorption of contaminants that are not part of soil metabolomes. To this end, black polyethylene film microplastics (PE_black-film_), white polyethylene film microplastics (PE_white-film_), and pure polyethylene microplastic granules (PE_pure-granule_) were selected as typical polyethylene (PE) microplastics, which are some of the most abundant microplastics in soil.^32^ Three types of soil, including a mollisol soil (M), fluvo-aquic soil (F), and red soil (R), were collected to obtain different soil metabolomes. Both non-targeted and targeted metabolomics, along with several spectroscopic techniques, were used to test the formation of eco-coronas on microplastics based on soil metabolites. To investigate the impact of the eco-corona on contaminant sorption, dibutyl phthalate (DBP), a priority pollutant among phthalates and the major contributor to phthalate pollution in soil,^33^ was selected as a proxy for a co-occurring soil contaminant for sorption experiments to microplastics with or without an eco-corona.

## Materials and Methods

### Soil Collection and Reagents

The mollisol soil, fluvo-aquic soil, and red soil were collected in Jiamusi City (Heilongjiang Province), Xinxiang City (Henan Province), and Yingtan City (Jiangxi Province), China, respectively. All soil samples were transported back to the laboratory in a cooler and stored at −80 °C. Part of the soil was air-dried and passed through a 2 mm screen to measure soil properties. The soil properties were analyzed according to standard procedures (Table S1, Supporting Information).^34^ Black and white PE films were purchased from Min Feng Plastic Film Company (Shandong, China). Pure PE granules used for the production of plastic products were purchased from Sigma-Aldrich (China). The PE microplastics were prepared by freezing, grinding, and sieving to 250–600 μm and were denoted PE_black-film_, PE_white-film_, and PE_pure-granule_. Methanol (chromatographic purity), DBP (purity ≥ 99%), and LC–MS grade water were purchased from Ehrenstorfer GmbH (Germany) and Accustandard Inc. (USA), respectively. Sodium azide (NaN_3_) and bovine serum albumin (BSA, UniProtKB P02769, purity ≥ 98%, MW = 65 kDa) were purchased from Sigma-Aldrich (China).

### Extraction of Soil Metabolites

Fumigation was conducted to promote microbial cell lysis in order to release intracellular metabolites.^20^ Water was used as the extractant to efficiently extract water-soluble intracellular and extracellular metabolites (WESMs) from soils after fumigation.^20^ Here, a total of 100 g of soil was weighed and transferred into 250 mL beakers, which were then placed in a vacuum desiccator. Simultaneously, three beakers containing 15 mL of ethanol-free chloroform (with a small amount of anti-overflow glass boiling beads in the beakers) and a small beaker containing NaOH solution (to absorb CO_2_ released during fumigation) were placed in the vacuum desiccator. Then, using a vacuum pump, a vacuum was generated in the desiccator to let the ethanol-free chloroform boil for 5 min. The valve of the vacuum desiccator was closed, and the desiccator was incubated at 25 °C for 24 h in darkness. All extracts were filtered through a 0.22 μm cellulose acetate membrane using LC–MS-grade water (+0.02% NaN_3_) (pH 6.92). Sodium azide was added to inhibit bacterial and biofilm formation during the experimental process. Soil (5 g, fumigated) and extractant (soil–water ratio of 1:4) were added to a 50 mL amber glass centrifuge tube. Then, the centrifuge tube was placed in a shaker and oscillated at a speed of 200 revolution min^–1^ at 4 °C for 2 h. After oscillation, the liquid solutions were sampled and centrifuged at 3000 revolutions min^–1^ for 15 min, and the supernatant was filtered through a 0.45 μm filter into a 1000 mL amber glass (WESM solution) (the experimental procedures of this study are shown in Figure S1).

### Sorption of WESMs by Microplastics

Ten-milligram samples of microplastics (i.e., PE_black-film_, PE_white-film_, and PE_pure-granule_) were added to an amber glass vial containing 8 mL of WESM solution. The vials were oscillated in a rotating oscillator at 50 revolution min^–1^ under dark conditions at 25 °C. After 96 h, the samples were filtered through a 0.22 μm centrifugal membrane (blank controls were obtained before and after the procedure without microplastics). At the same time, pure water was used as the background solution to determine the compound composition released by the microplastics. All the treatments were conducted with four replicates. The filtrates were dried in a vacuum centrifuge concentrator (LGJ-18, Songyuan Huaxing Company, China) for 6–8 h. After drying, 120 μL of complex solution (acetonitrile/water = 1: 1) was added to the sample vial for redissolution, followed by low-temperature ultrasonic extraction for 5 min (5 °C, 40 kHz), centrifugation at 4 °C, and centrifugation at 13,000 revolution min^–1^ for 5 min. Then, the supernatant was removed and injected into the vial for analysis (Text S1).

### Characterization of the Eco-corona

The surface morphology of the microplastics was observed by scanning electron microscopy (SEM, Hitachi SU8010). A Fourier transform infrared (FTIR, Nicolet iS10) spectrometer in transmission mode was used to investigate the changes in functional groups on the surface of the microplastics. The surface-element composition was determined by X-ray photoelectron spectroscopy (XPS, ESCALAB 250 Xi) with a monochromatic Al source (1486.6 eV). Raman spectra of the microplastic surfaces were measured by confocal Raman microscopy (Raman, Renishaw inVia) with a 532 nm laser with a step of 600 nm and a 1 MPa tablet with a size of 50 × 50 μm. The CH and CH_2_ Raman bands (near 2850 cm^–1^) represent the main portion of PE microplastics, while the C–N stretching and amide III bands (near 1130 cm^–1^) were mapped to represent the potential adsorbed compounds that form the eco-corona. WiRE software (Version 5) was used to process the data (the ratio of the peak areas near 1130 and near 2850 cm^–1^) and visualize the spatial distribution.

### Direct Pathway of Eco-corona Formation by Sorption of Soil Metabolites

Based on the experimental sorption results, eight compounds, including all-trans-retinoic acid, thymine, aminocaproic acid, betaine, l-isoleucine, l-glutamic acid, phytosphingosine, and deoxyadenosine, were screened as the typical components of soil metabolites that form eco-coronas on microplastics. The detailed screening principle is shown in Text S2. Thus, target metabolomics was conducted to quantify the sorption of these eight compounds onto microplastics. In the sorption kinetic experiments, 0.4 mL of a 500 μg L^–1^ mixed standard solution of the eight metabolites was added to 19.6 mL of background solution (LC–MS water with 0.02% NaN_3_) to obtain a concentration of 10 μg L^–1^ for all eight metabolites in the vials. Then, 10 mg of microplastics (i.e., PE_black-film_, PE_white-film_, or PE_pure-granule_) was added to each vial. The vials were oscillated in a rotating oscillator at 50 revolution min^–1^ under dark conditions at 25 °C. The sampling time was set as 1, 2, 24, 48, and 72 h. The liquid solutions were sampled and centrifuged at a high speed of 10,000 revolution min^–1^ for 3 min. Ultra-performance liquid chromatography–tandem mass spectrometry (UPLC–MS/MS) was used for qualitative and quantitative detection of the target substances in the samples (Text S3).

### BSA Coating of Microplastics

BSA was used as a model large molecular compound to investigate the role of macromolecular soil metabolites on the formation of the eco-corona. BSA was selected due to its being a well characterized macromolecule commonly used in soil studies to understand protein interactions and because it was previously found to have a high affinity toward microplastics.^35,36^ Ten-milligram samples of microplastics (i.e., PE_black-film_, PE_white-film_, and PE_pure-granule_) were added to amber glass vials containing 8 mL of 100 mg L^–1^ BSA solution (+0.02% NaN_3_). The vials were oscillated in a rotating oscillator at 50 revolution min^–1^ under dark conditions at 25 °C. After 96 h, the microplastics loaded with BSA were filtered out. The 0.4 mL of mixed standard sample of the eight metabolite solutions mentioned above was added to 19.6 mL of background solution (LC–MS water with 0.02% NaN_3_) to obtain each metabolite concentration of 10 μg L^–1^ in the vials. Then, the microplastics loaded with BSA were added to each amber glass vial. The vials were oscillated in a rotating oscillator at 50 revolution min^–1^ under dark conditions at 25 °C. After 96 h, the samples were filtered through a 0.22 μm centrifugal membrane (a blank control before and after treatment was set without microplastics), with three replicates for each treatment. Following this, the supernatant was removed and injected into the vial for analysis. As a part of the system conditioning and quality control process, a pooled quality control (QC) sample was prepared by mixing equal volumes of all samples. Moreover, circular dichroism (CD, J-1500, JASCO Corporation, Tokyo, Japan) was used to evaluate the effect of microplastics on the secondary structure of BSA molecules. The detection wavelength range was 190–300 nm with a resolution of 1 nm, and the nitrogen flow rate was set to 3 L min^–1^.^37^ The microplastics did not significantly change the structure of BSA in the short term (Figure S2).

### Influence of the Eco-corona and WESMs on the Sorption of DBP

DBP, a widely used plasticizer, was selected as a representative soil contaminant. WESM solutions extracted from the mollisol soil, fluvo-aquic soil, and red soil (Figure S1) were used as the background solutions, and an LC–MS water (+0.02% NaN_3_) treatment group was set as the blank control. In the sorption kinetic experiments, 0.4 mL of 100 mg L^–1^ DBP standard solution was added to 19.6 mL of background solution to obtain a DBP concentration of 2 mg L^–1^ in the vials. Then, 10 mg of microplastics (i.e., PE_black-film_, PE_white-film_, or PE_pure-granule_) was added to each vial. The vials were oscillated in a rotating oscillator at 50 revolution min^–1^ under dark conditions at 25 °C. The sampling time was set as 0, 1, 2, 4, 8, 12, 24, 48, 72, and 96 h. In the sorption isotherm experiments, 0.4 mL aliquots of 25, 50, 75, 100, 175, 250, 375, and 500 mg L^–1^ DBP standard solutions were added to obtain DBP concentrations of 0.5, 1, 1.5, 2, 3.5, 5, 7.5, and 10 mg L^–1^, respectively. To clarify the influence of eco-corona formation on DBP sorption on microplastics, eco-corona-formed microplastics were used for the sorption experiment with water as the background solution, and the concentrations of DBP were set as 2, 5, and 10 mg L^–1^. The liquid solutions were sampled and centrifuged at a high speed at 10,000 revolution min^–1^ for 3 min, and the supernatant was analyzed by high-performance liquid chromatography (HPLC) to determine the concentration of DBP (Text S4).

### Quality Control and Data Analysis

The results of the metabolomics raw data analysis are shown in Text S5. The diversity of the metabolite community was expressed by the Shannon index, one-way ANOVA was performed with Statistical Product and Service Solutions (SPSS V20.0), and significant differences were compared by Duncan’s test at the *p* ≤ 0.05 level. Origin Pro (version 8.5) was used for data analysis and plotting. Collinearity analysis was performed using Cytoscape 3.8.0 and Gephi, and data with a Spearman correlation coefficient *r* < 0.7 and *p* > 0.05 were excluded from the collinearity data. Blank experiments with either no DBP or only DBP added to the solutions were conducted. Preliminary tests were performed to confirm that the PE microplastics would neither release DBP nor other phthalates during the sorption experiment, which may have been present as an additive within the selected PE microplastics.^33^ The average recovery of DBP was 96.76 ± 6.56%. In the sorption experiments, the average sorption efficiency of DBP ranged from 78.71 to 82.47% (relative standard deviation ≤ 2.16%), indicating good reproducibility. Pseudo-first-order and pseudo-second-order kinetics were used to fit the kinetic experimental results. Linear and Freundlich sorption isothermal models were used to describe the sorption process of DBP on microplastics (Table S2).

## Results

### Eco-corona Formation on Microplastic Surfaces

**Figure 1:**
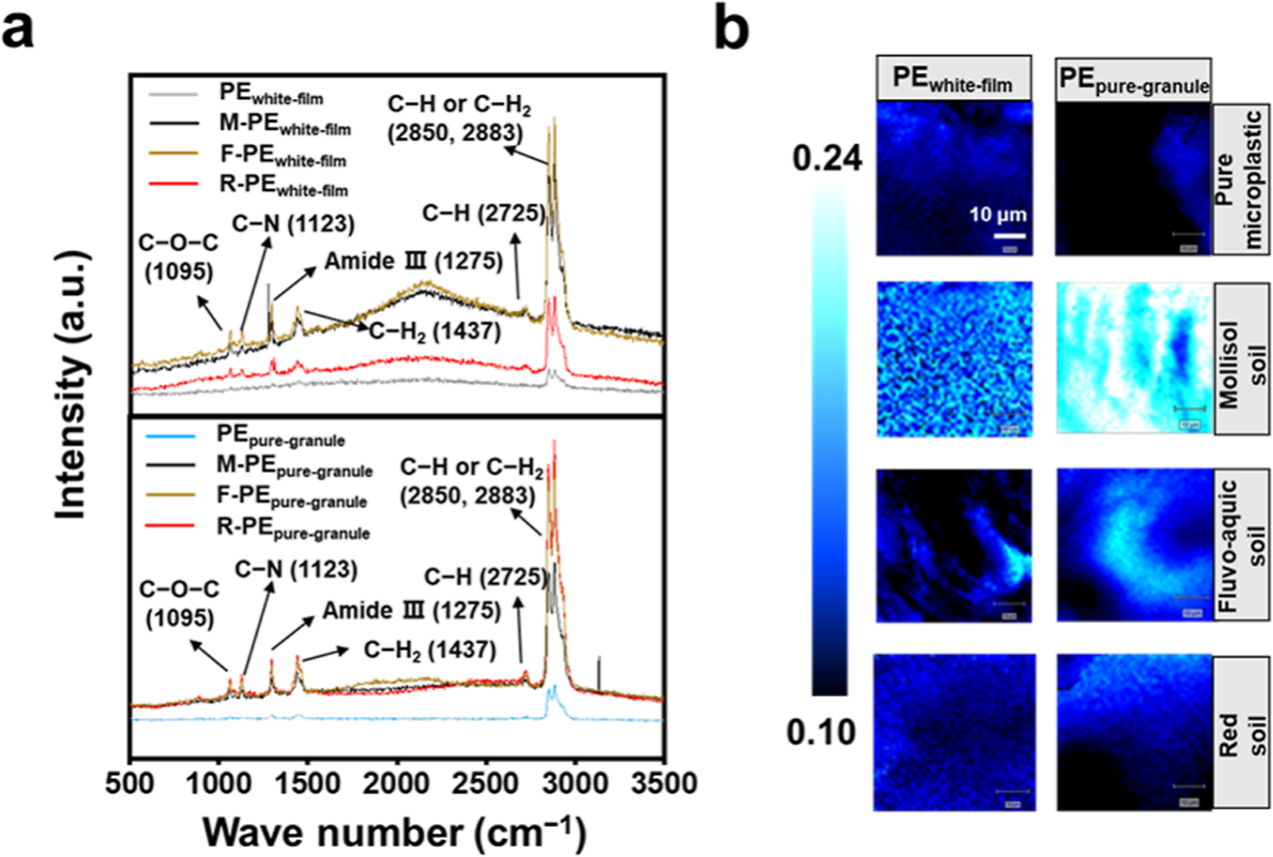
Confocal micro-Raman spectroscopy analysis of the coating on microplastic particles incubated with the water extracted soil metabolites from a mollisol soil (M), a fluvo-aquic soil (F), and a red soil (R), respectively. (a) Raman spectra. (b) False color Raman image of the microplastic particles (dark) and the biomolecules forming a putative eco-corona (light) on their surfaces, generated from the spectral mapping data. The brightness of the color represents the relative percentage of eco-corona on the surface of microplastics; the lighter the color, the larger the percentage of eco-corona. Window size, 50 × 50 μm. PE_white-film_: white polyethylene film microplastics; PE_pure-granule_: pure polyethylene microplastic granules. The black polyethylene film microplastics cannot be analyzed by Raman detection because of the limitation of the detection technology.^42^

The WESMs were extracted after soil fumigation and represented all extracellular and intracellular metabolites in the soil. Several lines of evidence were accumulated demonstrating that WESMs were part of the formation of the eco-corona. First, the significant decrease in dissolved organic carbon (DOC) concentration in the sorption experiment indicates that considerable amounts of WESMs were sorbed by microplastics within 72 h, regardless of the soil and microplastic types (Figure S3). This was further confirmed by comparing SEM images of microplastics before and after the sorption of WESMs, showing that the surface of the microplastics became more wrinkled and textured after WESM interactions (Figure S4). Third, Raman spectroscopy showed that the C–C bending of fatty acids (1061 cm^–1^), the C–O–C stretching of carbohydrates (1095 cm^–1^), the C–N stretching of lipids or proteins (1123 cm^–1^), the C–N stretching of the amide III bond of amino compounds (1275 cm^–1^), and the CH and CH_2_ stretching of lipids (1437, 2725, 2883–2850 cm^–1^) on the surface of PE_white-film_ and PE_pure-granule_ were different before and after interaction with WESMs (Figure 1a).^38,39^ Moreover, FTIR and XPS were used to detect the property changes due to the interactions of microplastics with WESMs, and the results showed that the absorption peak intensity of several bonds increased after the interaction with WESMs, including C=C or C=O, C–O–C, saturated C–H, unsaturated and aromatic C–H, and C–C (Figure S5a).^40,41^ XPS analysis demonstrated an increase in C, N, and S in the microplastics that interacted with WESMs, which complements the Raman and FTIR results (Figure S5b). Finally, microscopic Raman imaging was used to directly observe the eco-corona on the surface of microplastics (Figure 1b). The brightness of the color represents the proportion of eco-corona on the surface of the microplastics, and a lighter color represents a larger proportion of eco-corona. After interacting with WESMs, the surface of the microplastics displayed a larger size and increased strength of the eco-corona region (light). The light regions in the microplastics without exposure to WESMs may be related to the additives added during plastic production or other impurities on the surface (e.g., those acquired during transport or by sorption from the atmosphere). Therefore, these results collectively suggested that WESMs form an eco-corona on PE microplastic surfaces within days after the microplastics enter the soil.

### Eco-corona Is Mainly Derived from Soil WESMs Rather Than Absorbed Compounds Released from Microplastics

**Figure 2:**
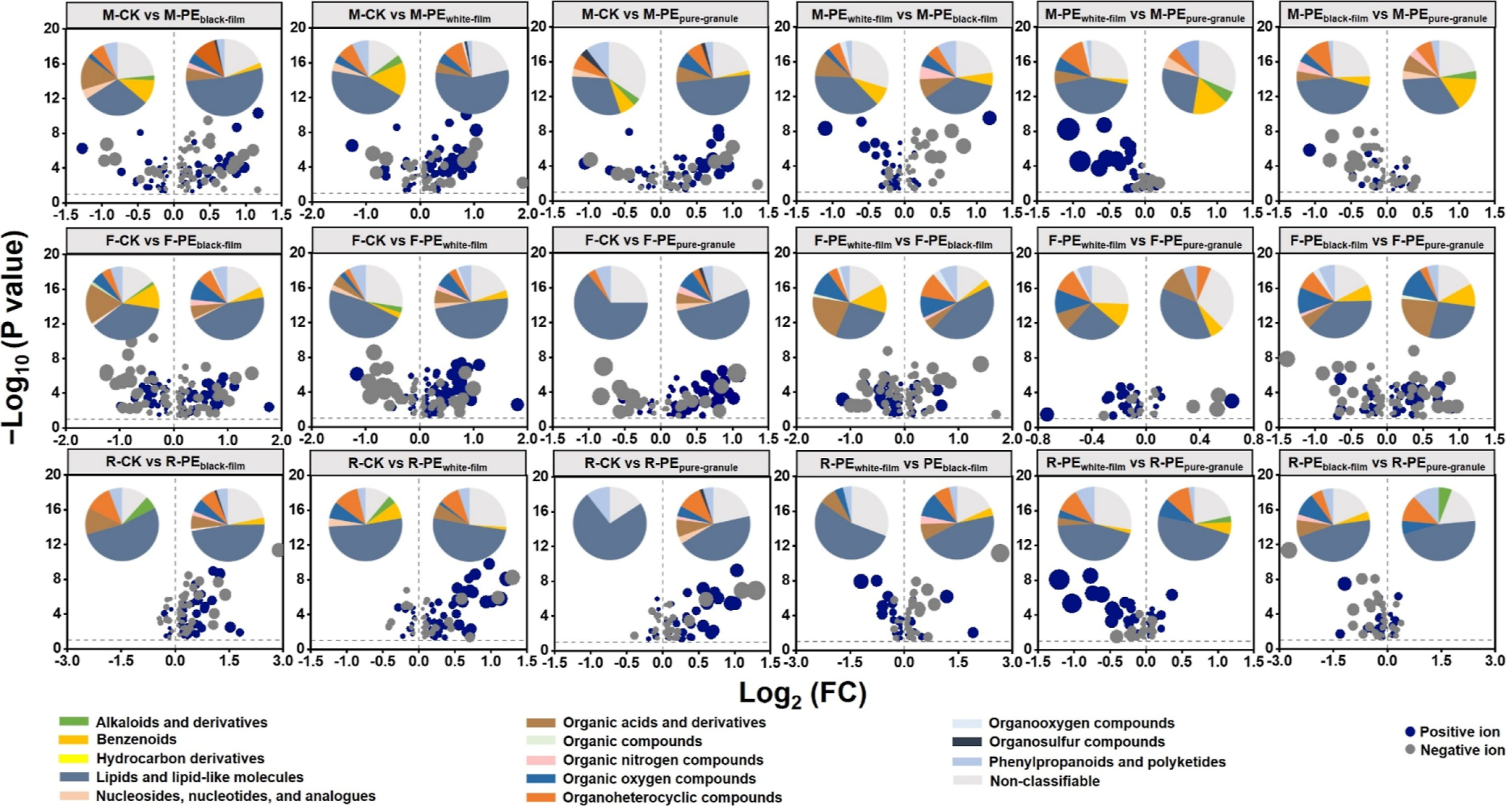
Volcano-plot of fold changes (FC) in water extracted soil metabolites quantified when microplastics were introduced to different soil solutions [mollisol soil (M), red soil (R), and fluvo-aquic soil (F)]. The pie charts compare the distribution of the soil metabolites present in the two systems indicated. CK: solution without microplastics; PE_black-film_: black polyethylene film microplastics; PE_white-film_: white polyethylene film microplastics; PE_pure-granule_: pure polyethylene microplastic granules. The FC value is quantified as the ratio of the concentrations of each metabolite group in the two systems. For example, within the chart of M-CK vs M-PE_black-film_, FC = M-PE_black-film_/M-CK. The positive Log_2_(FC) values mean those metabolites were abundant in M-PE_black-film_, while the negative Log_2_(FC) values mean those metabolites were abundant in M-CK. The −Log_10_(p value) higher than 1.30 indicates that the significance of all data was in a level of *p* ≤ 0.05.

A total of 16,434 compounds were detected among the three soil types, of which 605 compounds could be identified (Table S3). Significant changes in WESM composition were observed 72 h after mixing microplastics with different soil types (Figure S6). The WESMs identified were distinct among the three soil types (Figures S6 and S7). The number of common metabolites across all three soil types whose abundance significantly decreased (*p* ≤ 0.05) after the interaction with microplastics (between 140 and 165) was larger than the number of soil-specific metabolites that significantly decreased (Figure S8). This implies some similarity in the composition of the eco-corona across all three soils. In general, the presence of microplastics resulted in a significant (*p* ≤ 0.05) decrease in lipids and lipid-like molecules, benzenoids, alkaloids and derivatives, nucleosides, nucleotides and analogues, phenylpropanoids and polyketides, organoheterocyclic compounds, and other compounds in WESMs (Figure 2). Considering specific chemical substances, the main compounds that decreased in concentration after interaction between WESMs and microplastics included oleragenoside, cyclopassifloside x, nevskin, all-trans-retinoic acid, ajmaline, austalide j, and others (Figure S9a). The main elements contained in these decreased compounds are C, H, O, and N, and the main functional groups are C–C, C–O–C, C–N, amide bonds, lipid CH and CH_2_, etc., consistent with the spectroscopic results above.

The analysis of the additives released by the microplastics indicated that although the three types of microplastics could release diverse compounds, including lipids and lipid-like molecules, benzenoids, organooxygen compounds, organic acids and derivatives, and others (Figures S10 and S11), no one compound appeared in the top 50 of the WESMs that decreased when exposed to the microplastics mentioned above. However, nine released compounds were identified as being among the top 50 WESMs that increased in concentration (Figure S9b). This indicated that part of the enriched WESMs in the surrounding water originated from the microplastics, but microplastic-released additives were not major components of the eco-corona. It may be the case for other types of microplastics; additives could be a more substantial part of the WESM solution than observed here. Considering the impacts of microplastics in soil, it is interesting that adding microplastics significantly impacted the diversity and composition of WESMs (Figure S12) by both decreasing WESMs through the adsorption of soil metabolomes and the co-occurrent release of plastic additives. Thus, the interaction network structure of the WESMs became enhanced after interacting with microplastics (Table S4, Figure S13).

### Sorption of WESM Components to Microplastics

**Figure 3:**
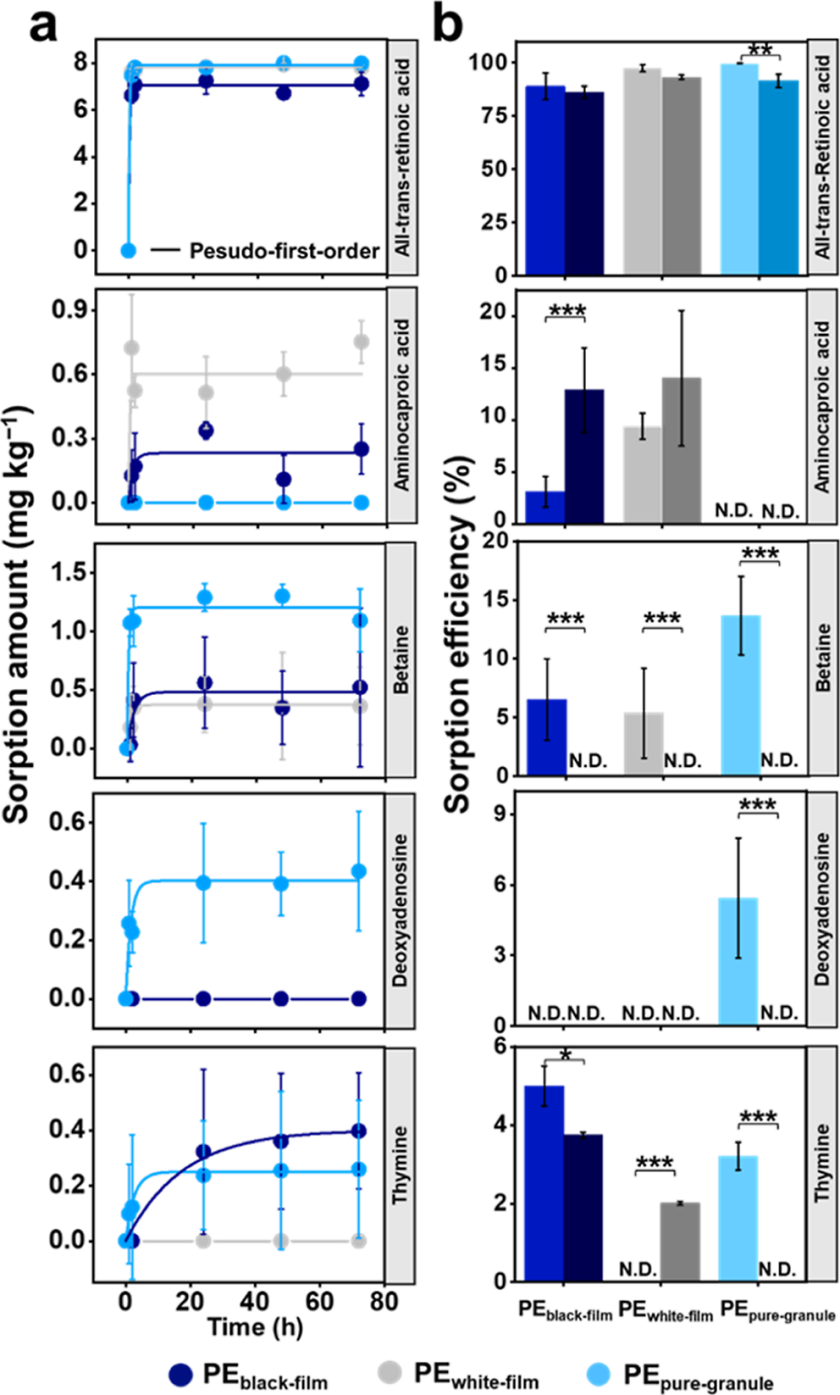
Sorption of all-trans-retinoic acid, aminocaproic acid, betaine, deoxyadenosin, and thymine on microplastics. The sorption kinetics of them (a) on black polyethylene film microplastics (PE_black-film_), white polyethylene film microplastics (PE_white-film_), and pure polyethylene microplastic granules (PE_pure-granule_) in water and the sorption efficiency of them onto microplastics under different treatment conditions (b). The color order of each microplastic treatment in (b) represents the sorption on microplastics in water (light) and the sorption on bovine serum albumin-coated microplastics in water (dark) (***p ≤ 0.001, **p ≤ 0.01, and *p ≤ 0.05). Raw data can be found in Table S5.

The concentrations of eight commercially available compounds, including all-trans-retinoic acid, thymine, aminocaproic acid, betaine, l-isoleucine, l-glutamic acid, phytosphingosine, and deoxyadenosine, were significantly decreased in the WESM solution after interaction with microplastics (Figure S14). Thus, target metabolomics was conducted to test their direct sorption on microplastics. Direct adsorption of all-trans-retinoic acid, aminocaproic acid, betaine, deoxyadenosine, and thymine on microplastics was observed, and equilibrium was quickly reached within 24 h except for the sorption of thymine on PE_white-film_ (Figure 3a, Table S5). Proteins form an integral part of macromolecules in soil solution and have been found to have a high affinity toward microplastics.^35,36^ It was found that there was a dual effect of BSA on the sorption behavior of soil metabolites on microplastics. BSA indeed facilitated the sorption of thymine on PE_white-film_ and increased the sorption efficiency of aminocaproic acid on the film microplastics (Figure 3b), showing increased bridging interactions for the sorption of certain soil metabolites on microplastics. Furthermore, the presence of BSA on the microplastics reduced the sorption of all-trans-retinoic acid, betaine, and deoxyadenosine on the microplastics (Figure 3b). Therefore, these results collectively suggested that eco-coronas can form through both direct sorption to the surface of the microplastics or, in some cases, indirect sorption of soil metabolites to molecules already on the microplastics. There was no sorption of l-isoleucine, l-glutamic acid, or phytosphingosine on microplastics in this target metabolomic test, suggesting that these compounds may be sorbed by molecules other than BSA on the surface of microplastics at the pH of the experiments conducted (Table S6).

### Eco-corona and WESMs Reduce the Sorption of Organic Contaminants to Microplastics

The DBP sorption results could be well described by the pseudo-first-order and pseudo-second-order kinetic models and the linear and Freundlich isotherm models (*R*^2^ > 0.97) (Table S7, Figure S15). Compared to the sorption results of DBP in pure water, the presence of WESMs in the aqueous phase significantly reduced the sorption capacity of DBP on microplastics (*p* ≤ 0.05). This could be due to two co-occurring mechanisms: (i) WESMs forming the eco-corona outcompete DBP for adsorption sites on microplastics, and (ii) co-solubilization occurs with soil metabolites in the surrounding aqueous solution.^43^ The sorption rates (*k*_1_ and *k*_2_) of DBP on PE microplastics were generally significantly higher in the pure water solutions than in the WESM solutions (*p* ≤ 0.05) (Table S7). The sorption of DBP and the sorption of WESMs forming the eco-corona appear to occur simultaneously in this experiment.

**Figure 4:**
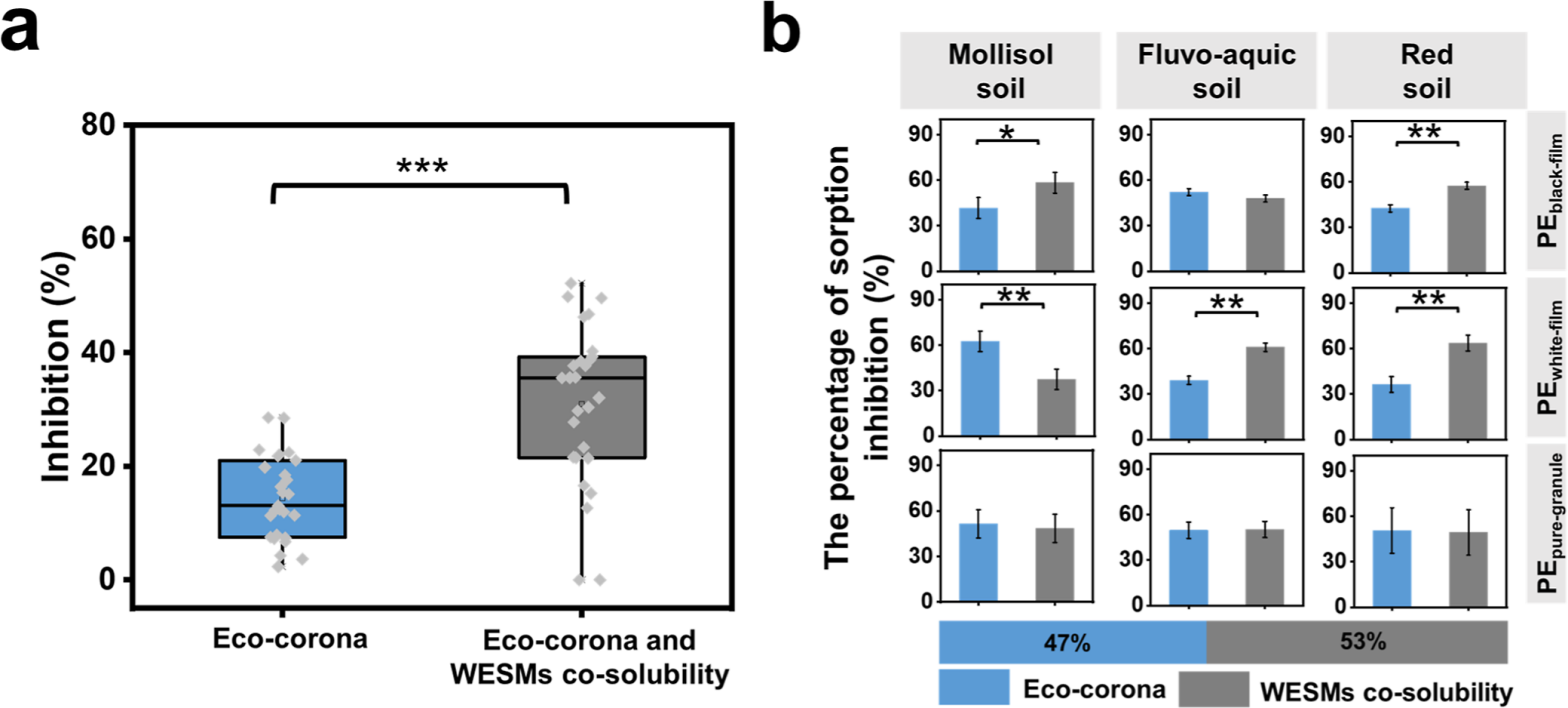
Effects of eco-corona and water extracted soil metabolites (WESMs) co-solubility on the sorption reduction of dibutyl phthalate (DBP) on microplastic. (a) Box and whisker plot of the inhibition ratio due solely to the eco-corona on the sorption of DBP, vs the inhibition ratio of eco-corona and WESM’s co-solubility on the sorption of DBP, both are compared to the sorption of DBP to pure microplastics in water. (b) Relative contributions of eco-corona and WESMs co-solubility to inhibit the sorption of DBP on microplastics. The inhibition is quantified as the ratio of reduction of two system partition ratio (*K*_P_ = *C*_sorbed_/*C*_water_), e.g., for eco-corona, inhibition = (*K*_P-ck_ – *K*_P-eco-corona_)/*K*_P-ck_; for WESMs, inhibition = (*K*_P-ck_ – *K*_P-WESMs_)/*K*_P-ck_. *K*_P-ck_ denotes the partition coefficient of DBP sorption on microplastics in pure water. PE_black-film_: black polyethylene film microplastics; PE_white-film_: white polyethylene film microplastics; PE_pure-granule_: pure polyethylene microplastic granules (***p ≤ 0.001, **p ≤ 0.01, and *p ≤ 0.05).

The sorption of DBP on eco-corona-formed microplastics and pure microplastics was compared, showing a 14.4% decrease in the sorption of DBP on eco-corona-formed microplastics relative to pure microplastics (Figure 4a), which can be attributed to the role of outcompeting adsorption (and negligible bridging interactions^44^). This decrease is significantly lower than the inhibition ratio of WESMs on the sorption of DBP compared to pure microplastics in water (*p* ≤ 0.05) (Figures 4a, S16). These tests with the eco-corona-formed microplastics also support the results in pure water that WESMs could inhibit the sorption of DBP on PE microplastics by (i) forming an eco-corona on the surface of microplastics, thus lowering the number of adsorption sites on the microplastics and providing a barrier effect; and (ii) enhancing the solubilization of DBP in the WESM solution. The relative contributions of both mechanisms to the inhibition of DBP sorption on microplastics were further calculated. Generally, the eco-corona inhibition effect (47%) on the sorption of DBP on microplastics was slightly lower than the WESM co-solubility effect (53%). The inhibition effect of the eco-corona was, however, significantly higher than that of WESM co-solubility, as in the case of PE_white-film_ in mollisol soil (Figure 4b). These findings on the influence of the eco-corona on the sorption mechanism provide new insights into the interactions between microplastics and other contaminants in soil.

## Discussion and Environmental Implications

The spectroscopic techniques as well as the non-targeted and targeted metabolomics approaches used here support hypothesis (i) that a soil metabolite-based eco-corona could readily and rapidly form on the surfaces of PE microplastics. The formation of the eco-corona on microplastics with soil metabolomes is a spontaneous and fast process. The eco-corona was formed within 72 h of interaction with WESMs (Figure 3a). A close inspection of the sorption kinetics of the selected metabolites with target metabolomics showed that sorption approached equilibrium within 24 h. The interaction of nanoplastics with biogenic extracellular polymers was previously found to form an eco-corona within 24 h.^24,45−47^ These results collectively indicate that an eco-corona from the soil metabolome can form quickly and remain relatively stable over several days to weeks on material surfaces.

The metabolomic analysis did not strictly support hypothesis (ii) that the eco-corona would be soil-specific due to the diversity of soil metabolomes. Although a minority of identified WESMs were soil-specific (with a maximum of 72 soil-specific WESMs in the case of fluvo-aquic soil and PE_white-flim_), the majority of WESMs were independent of soil type (with a minimum of 140 common WESMs for all soil types and PE_black-film_) and consisted of commonly observed WESMs (Figure S8). It should also be emphasized that the three different soil metabolomes themselves showed significant differences by principal component analysis (PCA) (Figure S6), but nevertheless, common WESMs were more likely to form an eco-corona. This indicates that the main selective combination of certain soil metabolites with PE microplastics is independent of soil type. These compounds, including lipids and lipid-like molecules, phenylpropanoids and polyketides, organoheterocyclic compounds, nucleosides, nucleotides and their analogues, are quite common components in soil solution (Figures 2, S8). PE microplastics, as semicrystalline polymers with a high hydrophobicity, have been reported to be strong sorbents for highly hydrophobic compounds (polycyclic aromatic hydrocarbons),^48,49^ as well as many other substances, in studies with PE as a passive sampler.^50−52^ Therefore, it is inevitable that these eco-corona components can be rapidly adsorbed on PE microplastics. Therefore, for future studies, it would be more fitting to phrase hypothesis (ii) as follows: the majority of metabolites in the eco-corona are common across soil metabolomes and consist of commonly occurring hydrophobic components.

The formation of the eco-corona could occur via direct sorption of soil metabolites on PE microplastic surfaces, or the eco-corona could form as a second layer by sorption of soil metabolites to macromolecules already on the surface. Targeted metabolomics analysis confirmed the direct sorption of all-trans-retinoic acid, aminocaproic acid, betaine, deoxyadenosine, and thymine. All-trans-retinoic acid was easily adsorbed by materials with hydrophobic moieties due to its high hydrophobicity^53^ and presented a higher sorption efficiency than the other metabolites on PE microplastics (Figure 3, Table S8). This result further indicates that soil metabolites with high hydrophobicity readily form an eco-corona on PE microplastics. Second, the sorption of thymine on BSA-coated PE_white-film_ and the increased sorption of aminocaproic acid on the three BSA-coated microplastics confirmed that macromolecules could act as a “bridge” in the adsorption of certain soil metabolites on microplastics. It was reported that hydrogen bonding and electrostatic interactions of exopolymeric substances on nanoplastic surfaces are enhanced in a protein-containing environment, facilitating the sorption of these substances on nanoplastics.^54^ Here, we also found an inhibitory effect of BSA on the sorption of aminocaproic acid, betaine, and deoxyadenosine on microplastics at the same time, indicating that BSA coated on microplastics could outcompete or inhibit the sorption sites for these metabolites. Thus, the effect of macromolecules on the sorption of metabolites is difficult to generalize when considering hundreds of compounds in WESMs. The presence of macromolecules is also an important consideration, as proteins are not always the most abundant constituent in the eco-corona when they form outside an organism.^55^

Previous studies have shown that DOM reduces the sorption capacity of organic contaminants on microplastics, which is mainly explained by DOM enhancing the co-solubilization of hydrophobic contaminants in the liquid phase.^43,56,57^ Here, the eco-corona was confirmed to act as a barrier inhibiting the adsorption of organic contaminants on the surface of microplastics, which supports our hypothesis (iii). This is particularly relevant for smaller microplastics, as the smaller the size of the microplastic is, the larger the specific surface area for adsorption. The relative contributions of the eco-corona to this inhibitory sorption effect were quite similar to the solubilizing effect of DOM (Figure 4). It was previously found that for PE microplastics, the more crystalline the PE structure was, the more adsorptive processes dominated over absorptive processes;^58^ therefore, it is to be expected that the impact of the eco-corona on sorption reduction would be more pronounced with increasing microplastic crystallinity.

Eco-corona formation is one way microplastics can influence soil function and health, such as by having a larger impact on the soil microbial community and potentially processes such as soil carbon storage and nutrient cycling driven by microbes.^59−62^ Such mechanisms are complex.^63^ Microbes regulate the composition and turnover of WESMs, and in turn, WESMs influence the diversity, structure, and function of microbial communities.^64^ It has been demonstrated that microbial attachment to microplastic surfaces is controlled in a substrate-dependent manner, and the eco-corona can directly influence the composition of biofilms on microplastic surfaces.^65^ Thus, the relationship between the eco-corona and the subsequent biofilm on microplastics and the dynamics of this relationship need further research. The formation of an eco-corona on microplastics with soil metabolomes is a universal phenomenon that changes the surface morphology, functional groups, and elemental composition of PE microplastics (Figures 1, S2 and S3) and thereby the environmental fate of plastic particles in soil through sorption and weathering processes.^66−68^ The eco-corona on nanomaterials has been reported to change the internalization, transport, toxicity, aggregation, etc., of particles in diverse organisms.^24−26,39,65,69,70^ This study provides further insights into the eco-corona of microplastics to further develop an in-depth understanding of the dynamics, risks, and impacts of the accumulation of microplastics and co-occurring chemical contaminants in soil.

## Supporting Information Available

Detailed non-targeted analysis, screening principle of soil metabolites, targeted analysis, determination of dibutyl phthalate, data analysis, physicochemical properties of soils, experimental procedures, circular dichroism spectra of bovine serum albumin, parameters of model fitting, concentration of dissolved organic matter, characterization of microplastics (SEM, XPS, and FTIR), soil metabolite raw data, metabolomics results (PCA, volcano-plot, Venn plot, heatmaps and Shannon index, and co-occurrence network), compounds released from microplastics, abundance and properties of targeted metabolites, and sorption results (PDF)

## References

[ref1] Borrelle S. B.; Ringma J.; Law K. L.; Monnahan C. C.; Lebreton L.; McGivern A.; Murphy E.; Jambeck J.; Leonard G. H.; Hilleary M. A.; Eriksen M.; Possingham H. P.; De Frond H.; Gerber L. R.; Polidoro B.; Tahir A.; Bernard M.; Mallos N.; Barnes M.; Rochman C. M. Predicted growth in plastic waste exceeds efforts to mitigate plastic pollution. Science 2020, 369, 1515–1518. 10.1126/science.aba3656. 32943526

[ref2] Lau W. W. Y.; Shiran Y.; Bailey R. M.; Cook E.; Stuchtey M. R.; Koskella J.; Velis C. A.; Godfrey L.; Boucher J.; Murphy M. B.; Thompson R. C.; Jankowska E.; Castillo Castillo A.; Pilditch T. D.; Dixon B.; Koerselman L.; Kosior E.; Favoino E.; Gutberlet J.; Baulch S.; Atreya M. E.; Fischer D.; He K. K.; Petit M. M.; Sumaila U. R.; Neil E.; Bernhofen M. V.; Lawrence K.; Palardy J. E. Evaluating scenarios toward zero plastic pollution. Science 2020, 369, 1455–1461. 10.1126/science.aba9475. 32703909

[ref3] Rillig M. C.; Kim S. W.; Kim T. Y.; Waldman W. R. The global plastic toxicity debt. Environ. Sci. Technol. 2021, 55, 2717–2719. 10.1021/acs.est.0c07781. 33596648 PMC7931444

[ref4] Duan J.; Bolan N.; Li Y.; Ding S.; Atugoda T.; Vithanage M.; Sarkar B.; Tsang D. C. W.; Kirkham M. B. Weathering of microplastics and interaction with other coexisting constituents in terrestrial and aquatic environments. Water Res. 2021, 196, 117011 10.1016/j.watres.2021.117011. 33743325

[ref5] Souza Machado A. A.; Kloas W.; Zarfl C.; Hempel S.; Rillig M. C. Microplastics as an emerging threat to terrestrial ecosystems. Global Change Biol. 2018, 24, 1405–1416. 10.1111/gcb.14020. PMC5834940 29245177

[ref6] Wong J. K. H.; Lee K. K.; Tang K. H. D.; Yap P. S. Microplastics in the freshwater and terrestrial environments: Prevalence, fates, impacts and sustainable solutions. Sci. Total Environ. 2020, 719, 137512 10.1016/j.scitotenv.2020.137512. 32229011

[ref7] Kirstein I. V.; Kirmizi S.; Wichels A.; Garin-Fernandez A.; Erler R.; Loder M.; Gerdts G. Dangerous hitchhikers? Evidence for potentially pathogenic Vibrio spp. on microplastic particles. Mar. Environ. Res. 2016, 120, 1–8. 10.1016/j.marenvres.2016.07.004. 27411093

[ref8] Huerta Lwanga E.; Gertsen H.; Gooren H.; Peters P.; Salanki T.; van der Ploeg M.; Besseling E.; Koelmans A. A.; Geissen V. Microplastics in the terrestrial ecosystem: Implications for lumbricus terrestris (Oligochaeta, Lumbricidae). Environ. Sci. Technol. 2016, 50, 2685–2691. 10.1021/acs.est.5b05478. 26852875

[ref9] Zhu D.; Chen Q.; An X.; Yang X.; Christie P.; Ke X.; Wu L.; Zhu Y. Exposure of soil collembolans to microplastics perturbs their gut microbiota and alters their isotopic composition. Soil Biol. Biochem. 2018, 116, 302–310. 10.1016/j.soilbio.2017.10.027.

[ref10] Li L.; Luo Y.; Li R.; Zhou Q.; Peijnenburg W. J. G. M.; Yin N.; Yang J.; Tu C.; Zhang Y. Effective uptake of submicrometre plastics by crop plants via a crack-entry mode. Nat. Sustain. 2020, 3, 929–937. 10.1038/s41893-020-0567-9.

[ref11] Boots B.; Russell C. W.; Green D. S. Effects of microplastics in soil ecosystems: Above and below ground. Environ. Sci. Technol. 2019, 53, 11496–11506. 10.1021/acs.est.9b03304. 31509704

[ref12] Nel A.; Xia T.; Mädler L.; Li N. Toxic potential of materials at the nanolevel. Science 2006, 311, 622–627. 10.1126/science.1114397. 16456071

[ref13] Lehner R.; Weder C.; Petri-Fink A.; Rothen-Rutishauser B. Emergence of nanoplastic in the environment and possible impact on human health. Environ. Sci. Technol. 2019, 53, 1748–1765. 10.1021/acs.est.8b05512. 30629421

[ref14] Wright S. L.; Kelly F. J. Plastic and human health: A micro issue?. Environ. Sci. Technol. 2017, 51, 6634–6647. 10.1021/acs.est.7b00423. 28531345

[ref15] Seeley M. E.; Song B.; Passie R.; Hale R. C. Microplastics affect sedimentary microbial communities and nitrogen cycling. Nat. Commun. 2020, 11, 2372 10.1038/s41467-020-16235-3. 32398678 PMC7217880

[ref16] Roth V. N.; Lange M.; Simon C.; Hertkorn N.; Bucher S.; Goodall T.; Griffiths R. I.; Mellado-Vazquez P. G.; Mommer L.; Oram N. J.; Weigelt A.; Dittmar T.; Gleixner G. Persistence of dissolved organic matter explained by molecular changes during its passage through soil. Nat. Geosci. 2019, 12, 755–761. 10.1038/s41561-019-0417-4.

[ref17] MacLeod M.; Arp H. P. H.; Tekman M. B.; Jahnke A. The global threat from plastic pollution. Science 2021, 373, 61–65. 10.1126/science.abg5433. 34210878

[ref18] Evangeliou N.; Grythe H.; Klimont Z.; Heyes C.; Eckhardt S.; Lopez-Aparicio S.; Stohl A. Atmospheric transport is a major pathway of microplastics to remote regions. Nat. Commun. 2020, 11, 3381 10.1038/s41467-020-17201-9. 32665541 PMC7360784

[ref19] Kaiser K.; Kalbitz K. Cycling downwards - dissolved organic matter in soils. Soil Biol. Biochem. 2012, 52, 29–32. 10.1016/j.soilbio.2012.04.002.

[ref20] Swenson T. L.; Jenkins S.; Bowen B. P.; Northen T. R. Untargeted soil metabolomics methods for analysis of extractable organic matter. Soil Biol. Biochem. 2015, 80, 189–198. 10.1016/j.soilbio.2014.10.007.

[ref21] Junaid M.; Wang J. Interaction of nanoplastics with extracellular polymeric substances (EPS) in the aquatic environment: A special reference to eco-corona formation and associated impacts. Water Res. 2021, 201, 117319 10.1016/j.watres.2021.117319. 34130084

[ref22] Koelmans A. A.; Bakir A.; Burton G. A.; Janssen C. R. Microplastic as a vector for chemicals in the aquatic environment: Critical review and model-supported reinterpretation of empirical studies. Environ. Sci. Technol. 2016, 50, 3315–3326. 10.1021/acs.est.5b06069. 26946978 PMC6863595

[ref23] Zettler E. R.; Mincer T. J.; Amaral-Zettler L. A. Life in the “plastisphere”: Microbial communities on plastic marine debris. Environ. Sci. Technol. 2013, 47, 7137–7146. 10.1021/es401288x. 23745679

[ref24] Fadare O. O.; Wan B.; Liu K.; Yang Y.; Zhao L.; Guo L. Eco-corona vs protein corona: Effects of humic substances on corona formation and nanoplastic particle toxicity in Daphnia magna. Environ. Sci. Technol. 2020, 54, 8001–8009. 10.1021/acs.est.0c00615. 32464058

[ref25] Liu G.; Jiang R.; You J.; Muir D. C. G.; Zeng E. Microplastic impacts on microalgae growth: Effects of size and humic acid. Environ. Sci. Technol. 2020, 54, 1782–1789. 10.1021/acs.est.9b06187. 31809028

[ref26] Schultz C. L.; Bart S.; Lahive E.; Spurgeon D. J. What is on the outside matters-surface charge and dissolve organic matter association affect the toxicity and physiological mode of action of polystyrene nanoplastics to C. elegans. Environ. Sci. Technol. 2021, 55, 6065–6075. 10.1021/acs.est.0c07121. 33848142

[ref27] Sooriyakumar P.; Bolan N.; Kumar M.; Singh L.; Yu Y.; Li Y.; Weralupitiya C.; Vithanage M.; Ramanayaka S.; Sarkar B.; Wang F.; Gleeson D. B.; Zhang D.; Kirkham M. B.; Rinklebe J.; M Siddique K. H. Biofilm formation and its implications on the properties and fate of microplastics in aquatic environments: A review. J. Hazard. Mater. Adv. 2022, 6, 100077 10.1016/j.hazadv.2022.100077.

[ref28] Alimi O. S.; Farner Budarz J.; Hernandez L. M.; Tufenkji N. Microplastics and nanoplastics in aquatic environments: Aggregation, deposition, and enhanced contaminant transport. Environ. Sci. Technol. 2018, 52, 1704–1724. 10.1021/acs.est.7b05559. 29265806

[ref29] Velzeboer I.; Kwadijk C. J. A. F.; Koelmans A. A. Strong sorption of PCBs to nanoplastics, microplastics, carbon nanotubes, and fullerenes. Environ. Sci. Technol. 2014, 48, 4869–4876. 10.1021/es405721v. 24689832

[ref30] Wang C.; Zhao J.; Xing B. Environmental source, fate, and toxicity of microplastics. J. Hazard. Mater. 2021, 407, 124357 10.1016/j.jhazmat.2020.124357. 33158648

[ref31] Hartmann N. B.; Rist S.; Bodin J.; Jensen L. H. S.; Schmidt S. N.; Mayer P.; Meibom A.; Baun A. Microplastics as vectors for environmental contaminants: Exploring sorption, desorption, and transfer to biota. Integrated Environ. Assess. Manag. 2017, 13, 488–493. 10.1002/ieam.1904. 28440931

[ref32] He D.; Luo Y.; Lu S.; Liu M.; Song Y.; Lei L. Microplastics in soils: Analytical methods, pollution characteristics and ecological risks. TrAC, Trends Anal. Chem. 2018, 109, 163–172. 10.1016/j.trac.2018.10.006.

[ref33] Net S.; Sempere R.; Delmont A.; Paluselli A.; Ouddane B. Occurrence, fate, behavior and ecotoxicological state of phthalates in different environmental matrices. Environ. Sci. Technol. 2015, 49, 4019–4035. 10.1021/es505233b. 25730609

[ref34] Lu R. Analytical Methods for Soils and Agricultural Chemistry; China Agricultural Science and Technology Press, 1999.

[ref35] Schmidt M. P.; Martinez C. E. Supramolecular association impacts biomolecule adsorption onto goethite. Environ. Sci. Technol. 2018, 52, 4079–4089. 10.1021/acs.est.7b06173. 29516738

[ref36] Tan Y.; Zhu X.; Wu D.; Song E.; Song Y. Compromised autophagic effect of polystyrene nanoplastics mediated by protein corona was recovered after lysosomal degradation of corona. Environ. Sci. Technol. 2020, 54, 11485–11493. 10.1021/acs.est.0c04097. 32786567

[ref37] Jing P.; Li Y.; Su Y.; Liang W.; Leng Y. The role of metal ions in the behavior of bovine serum albumin molecules under physiological environment. Spectrochim. Acta, Part A 2022, 267, 120604 10.1016/j.saa.2021.120604. 34802930

[ref38] Movasaghi Z.; Rehman S.; Rehman I. U. Raman spectroscopy of biological tissues. Appl. Spectrosc. Rev. 2007, 42, 493–541. 10.1080/05704920701551530.

[ref39] Ramsperger A. F. R. M.; Narayana V. K. B.; Gross W.; Mohanraj J.; Thelakkat M.; Greiner A.; Schmalz H.; Kress H.; Laforsch C. Environmental exposure enhances the internalization of microplastic particles into cells. Sci. Adv. 2020, 6, eabd1211 10.1126/sciadv.abd1211. 33298447 PMC7725476

[ref40] Courtene-Jones W.; Quinn B.; Murphy F.; Gary S. F.; Narayanaswamy B. E. Optimisation of enzymatic digestion and validation of specimen preservation methods for the analysis of ingested microplastics. Anal. Methods 2017, 9, 1437–1445. 10.1039/c6ay02343f.

[ref41] Shim W. J.; Hong S. H.; Eo S. E. Identification methods in microplastic analysis: a review. Anal. Methods 2017, 9, 1384–1391. 10.1039/c6ay02558g.

[ref42] Lim X. Microplastics are everywhere - but are they harmful?. Nature 2021, 593, 22–25. 10.1038/d41586-021-01143-3. 33947993

[ref43] Seidensticker S.; Zarfl C.; Cirpka O. A.; Fellenberg G.; Grathwohl P. Shift in mass transfer of wastewater contaminants from microplastics in the presence of dissolved substances. Environ. Sci. Technol. 2017, 51, 12254–12263. 10.1021/acs.est.7b02664. 28965391

[ref44] Wijesekara H.; Bolan N. S.; Bradney L.; Obadamudalige N.; Seshadri B.; Kunhikrishnan A.; Dharmarajan R.; Ok Y. S.; Rinklebe J.; Kirkham M. B.; Vithanage M. Trace element dynamics of biosolids-derived microbeads. Chemosphere 2018, 199, 331–339. 10.1016/j.chemosphere.2018.01.166. 29448201

[ref45] Shiu R. F.; Vazquez C. I.; Chiang C. Y.; Chiu M. H.; Chen C.; Ni C.; Gong G.; Quigg A.; Santschi P. H.; Chin W. C. Nano- and microplastics trigger secretion of protein-rich extracellular polymeric substances from phytoplankton. Sci. Total Environ. 2020, 748, 141469 10.1016/j.scitotenv.2020.141469. 33113698

[ref46] Summers S.; Henry T.; Gutierrez T. Agglomeration of nano- and microplastic particles in seawater by autochthonous and de novo-produced sources of exopolymeric substances. Mar. Pollut. Bull. 2018, 130, 258–267. 10.1016/j.marpolbul.2018.03.039. 29866555

[ref47] Canesi L.; Ciacci C.; Fabbri R.; Balbi T.; Salis A.; Damonte G.; Cortese K.; Caratto V.; Monopoli M. P.; Dawson K.; Bergami E.; Corsi I. Interactions of cationic polystyrene nanoparticles with marine bivalve hemocytes in a physiological environment: Role of soluble hemolymph proteins. Environ. Res. 2016, 150, 73–81. 10.1016/j.envres.2016.05.045. 27257827

[ref48] Huffer T.; Hofmann T. Sorption of non-polar organic compounds by micro-sized plastic particles in aqueous solution. Environ. Pollut. 2016, 214, 194–201. 10.1016/j.envpol.2016.04.018. 27086075

[ref49] Wang W.; Wang J. Comparative evaluation of sorption kinetics and isotherms of pyrene onto microplastics. Chemosphere 2018, 193, 567–573. 10.1016/j.chemosphere.2017.11.078. 29169132

[ref50] Shen M.; Song B.; Zeng G.; Zhang Y.; Teng F.; Zhou C. Surfactant changes lead adsorption behaviors and mechanisms on microplastics. Chem. Eng. J. 2021, 405, 126989 10.1016/j.cej.2020.126989.

[ref51] Xu B.; Liu F.; Brookes P. C.; Xu J. The sorption kinetics and isotherms of sulfamethoxazole with polyethylene microplastics. Mar. Pollut. Bull. 2018, 131, 191–196. 10.1016/j.marpolbul.2018.04.027. 29886936

[ref52] Hale S. E.; Martin T. J.; Goss K. U.; Arp H. P. H.; Werner D. Partitioning of organochlorine pesticides from water to polyethylene passive samplers. Environ. Pollut. 2010, 158, 2511–2517. 10.1016/j.envpol.2010.03.010. 20398988

[ref53] Banerjee S. S.; Chen D. Cyclodextrin conjugated magnetic colloidal nanoparticles as a nanocarrier for targeted anticancer drug delivery. Nanotechnology 2008, 19, 265602 10.1088/0957-4484/19/26/265602. 21828683

[ref54] Chen C.; Anaya J. M.; Zhang S.; Spurgin J.; Chuang C.; Xu C.; Miao A.; Chen E. Y. T.; Schwehr K. A.; Jiang Y.; Quigg A.; Santschi P. H.; Chin W. C. Effects of engineered nanoparticles on the assembly of exopolymeric substances from phytoplankton. PLoS One 2011, 6, e21865 10.1371/journal.pone.0021865. 21811550 PMC3140995

[ref55] Wheeler K. E.; Chetwynd A. J.; Fahy K. M.; Hong B. S.; Tochihuitl J. A.; Foster L. A.; Lynch I. Environmental dimensions of the protein corona. Nat. Nanotechnol. 2021, 16, 617–629. 10.1038/s41565-021-00924-1. 34117462

[ref56] Jiang M.; Hu L.; Lu A.; Liang G.; Lin Z.; Zhang T.; Xu L.; Li B.; Gong W. Strong sorption of two fungicides onto biodegradable microplastics with emphasis on the negligible role of environmental factors. Environ. Pollut. 2020, 267, 115496 10.1016/j.envpol.2020.115496. 33254727

[ref57] Guo X.; Wang X.; Zhou X.; Kong X.; Tao S.; Xing B. Sorption of four hydrophobic organic compounds by three chemically distinct polymers: Role of chemical and physical composition. Environ. Sci. Technol. 2012, 46, 7252–7259. 10.1021/es301386z. 22676433

[ref58] Yao S.; Cao H.; Arp H. P. H.; Li J.; Bian Y.; Xie Z.; Cherubini F.; Jiang X.; Song Y. The role of crystallinity and particle morphology on the sorption of dibutyl phthalate on polyethylene microplastics: Implications for the behavior of phthalate plastic additives. Environ. Pollut. 2021, 283, 117393 10.1016/j.envpol.2021.117393. 34034021

[ref59] Zang H.; Blagodatskaya E.; Wen Y.; Xu X.; Dyckmans J.; Kuzyakov Y. Carbon sequestration and turnover in soil under the energy crop *Miscanthus*: repeated ^13^C natural abundance approach and literature synthesis. GCB Bioenergy 2018, 10, 262–271. 10.1111/gcbb.12485.

[ref60] Fu Q.; Lai J.; Ji X.; Luo Z.; Wu G.; Luo X. Alterations of the rhizosphere soil microbial community composition and metabolite profiles of Zea mays by polyethylene-particles of different molecular weights. J. Hazard. Mater. 2022, 423, 127062 10.1016/j.jhazmat.2021.127062. 34482080

[ref61] Santos R. G.; Machovsky-Capuska G. E.; Andrades R. Plastic ingestion as an evolutionary trap: Toward a holistic understanding. Science 2021, 373, 56–60. 10.1126/science.abh0945. 34210877

[ref62] de Souza Machado A. A.; Lau C. W.; Till J.; Kloas W.; Lehmann A.; Becker R.; Rillig M. C. Impacts of microplastics on the soil biophysical environment. Environ. Sci. Technol. 2018, 52, 9656–9665. 10.1021/acs.est.8b02212. 30053368 PMC6128618

[ref63] Lee Y. K.; Murphy K. R.; Hur J. Fluorescence signatures of dissolved organic matter leached from microplastics: Polymers and additives. Environ. Sci. Technol. 2020, 54, 11905–11914. 10.1021/acs.est.0c00942. 32852946

[ref64] Hu A.; Choi M.; Tanentzap A. J.; Liu J.; Jang K. S.; Lennon J. T.; Liu Y.; Soininen J.; Lu X.; Zhang Y.; Shen J.; Wang J. Ecological networks of dissolved organic matter and microorganisms under global change. Nat. Commun. 2022, 13, 3600 10.1038/s41467-022-31251-1. 35739132 PMC9226077

[ref65] Rummel C. D.; Lechtenfeld O. J.; Kallies R.; Benke A.; Herzsprung P.; Rynek R.; Wagner S.; Potthoff A.; Jahnke A.; Schmitt-Jansen M. Conditioning film and early biofilm succession on plastic surfaces. Environ. Sci. Technol. 2021, 55, 11006–11018. 10.1021/acs.est.0c07875. 34339175

[ref66] Dong Z.; Hou Y.; Han W.; Liu M.; Wang J.; Qiu Y. Protein corona-mediated transport of nanoplastics in seawater-saturated porous media. Water Res. 2020, 182, 115978 10.1016/j.watres.2020.115978. 32622130

[ref67] Wang F.; Wong C. S.; Chen D.; Lu X.; Wang F.; Zeng E. Interaction of toxic chemicals with microplastics: A critical review. Water Res. 2018, 139, 208–219. 10.1016/j.watres.2018.04.003. 29653356

[ref68] Arp H. P. H.; Kuhnel D.; Rummel C.; MacLeod M.; Potthoff A.; Reichelt S.; Rojo-Nieto E.; Schmitt-Jansen M.; Sonnenberg J.; Toorman E.; Jahnke A. Weathering plastics as a planetary boundary threat: Exposure, fate, and hazards. Environ. Sci. Technol. 2021, 55, 7246–7255. 10.1021/acs.est.1c01512. 33973471

[ref69] Baalousha M.; Afshinnia K.; Guo L. Natural organic matter composition determines the molecular nature of silver nanomaterial-NOM corona. Environ. Sci.: Nano 2018, 5, 868–881. 10.1039/c8en00018b.

[ref70] Li X.; He E.; Xia B.; Liu Y.; Zhang P.; Cao X.; Zhao L.; Xu X.; Qiu H. Protein corona-induced aggregation of differently sized nanoplastics: impacts of protein type and concentration. Environ. Sci.: Nano 2021, 8, 1560–1570. 10.1039/d1en00115a.

