# Supplementary material for: Soil Metabolome Impacts the Formation of the Eco-corona and Adsorption Processes on Microplastic Surfaces
Source: Environ Sci Technol. 2023 May 17;57(21):8139–48. doi: 10.1021/acs.est.3c01877 (PMC10233752; doi:10.1021/acs.est.3c01877)
Supplement: Supplementary file 1 — es3c01877_si_001.pdf [file es3c01877_si_001.pdf]

Supporting Information for

Environmental Science & Technology

Soil Metabolome Impacts the Formation of the Eco-corona and Adsorption Processes on Microplastic Surfaces

Shi Yao [a,b], Xiaona Li [c], Tao Wang [d], Xin Jiang [a,b], Yang Song [a,b,*], Hans Peter H. Arp [e,f]

[a] *CAS Key Laboratory of Soil Environment and Pollution Remediation, Institute of Soil Science, Chinese Academy of Sciences, Nanjing 210008, PR China*

[b] *University of Chinese Academy of Sciences, Beijing 100049, PR China*

[c] *School of Environmental Science and Engineering, Jiangnan University, Wuxi 225127, PR China*

[d] *Institute of Mountain Hazards and Environment, Chinese Academy of Sciences, Chengdu 610299, PR China*

[e] *Norwegian Geotechnical Institute (NGI), P.O. Box 3930, Ullevål Stadion, N-0806 Oslo, Norway*

[f] *Department of Chemistry, Norwegian University of Science and Technology (NTNU), NO-7491 Trondheim, Norway*

Corresponding author: Yang Song

Yang Song, E-mail: ysong@issas.ac.cn

The supporting information contains 51 pages including 5 texts, 16 figures and 8 tables.

Text S1.

Non-targeted analysis of the eco-corona. Chromatographic separation of the metabolites was performed on an ExionLCTMAD system (AB Sciex, USA) equipped with an ACQUITY UPLC BEH C18 column (100 mm × 2.1 mm i.d., 1.7 µm; Waters, Milford, USA). The mobile phases consisted of 0.1% formic acid in water with formic acid (0.1%) (Solvent A) and 0.1% formic acid in acetonitrile/isopropanol (1/1, v/v) (solvent B). The solvent gradient changed according to the following conditions: from 0 to 3 min, 95% (A): 5% (B) to 80% (A): 20% (B); from 3 to 9 min, 80% (A): 20% (B) to 5% (A): 95% (B); from 9 to 13 min, 5% (A): 95% (B) to 5% (A): 95% (B); from 13 to 13.1 min, 5% (A): 95% (B) to 95% (A): 5% (B), from 13.1 to 16 min, 95% (A): 5% (B) to 95% (A): 5% (B) for equilibrating the systems. The sample injection volume was 20 μL and the flow rate was set to 0.4 mL min^{-1}. The column temperature was maintained at 40 °C. During the period of analysis, all these samples were stored at 4 °C. The ultra-performance liquid chromatography–tandem mass spectrometry (UPLC-MS/MS) system was coupled to a quadrupole-time-of-flight mass spectrometer (Triple TOFTM5600+, AB Sciex, USA) equipped with an electrospray ionization (ESI) source operating in positive mode and negative mode. The optimal conditions were set as followed: source temperature, 500 °C; curtain gas (CUR), 30 psi; both Ion Source GS1 and GS2, 50 psi; ion-spray voltage floating (ISVF), −4000 V in negative mode and 5000 V in positive mode, respectively; decluttering potential, 80 V; a collision energy (CE), 20 − 60 V rolling for MS/MS. Data acquisition was performed with using the Data Dependent Acquisition (DDA) mode. The detection was carried out over a mass range of 50 − 1000 m/z. As a part of the system conditioning and quality control process, a pooled quality control sample (QC) was prepared by mixing equal volumes of all samples. The QC samples were disposed and tested in the same manner as the analytic samples. This pooled sample, which represents the whole sample set, would be injected at regular intervals (every 8 samples) in order to monitor the stability of the analysis.

Text S2.

The screening principle of soil metabolites were selected for targeted metabolomics. Certain soil metabolites were selected for targeted metabolomics analysis to ver.ify direct sorption and to characterize the adsorption kinetics on microplastics during the eco-corona formation process. The pure compounds used in this experiment were selected according to the following screening criteria: (i) the standard products of metabolites could be purchased; (ii) the metabolites have been found to play a role in the metabolic pathway in the database of Kyoto Encyclopedia of Genes and Genomes Pathway (https://www.genome.jp/kegg/pathway.html); (iii) the compounds appeared in metabolites of different types of soils and were sorbed by microplastics commonly; and (iv) the detection method was stable and sensitive.

Text S3.

Targeted analysis of the eco-corona. The chromatographic conditions were listed as follows: ExionLC AD system, Waters T3 column (100 × 2.1 mm, 1.8 μm), column temperature 45 °C, standard injection volume of 10 μL and balance for 2 min before injection. The mobile phases consisted of 0.1% formic acid in water with formic acid (0.1%) (Solvent A) and 0.1% formic acid in acetonitrile/isopropanol (1:1, v/v) (solvent B). The solvent gradient changed according to the following conditions: from 0 to 2 min, 98% (A):2% (B) to 98% (A): 2% (B); from 2 to 10 min, 98% (A): 2% (B) to 2% (A): 98% (B); from 10 to 14 min, 2% (A): 98% (B) to 2% (A): 98% (B) for equilibrating the systems. Mass spectrometry conditions: positive mode detection IS, Curtain Gas (CUR) IS 35, Collision Gas (CAD) IS Medium, IonSpray Voltage (IS) is 5500, temperature is 550, Ion Source Gas1 (GS1) is 50, and Ion Source Gas2 (GS2) is 50.

Text S4.

Determination and calculation of dibutyl phthalate (DBP) sorption on microplastics. The concentration of DBP in solution was determined by high-performance liquid chromatography (HPLC) (Agilent 1260 Infinity □) equipped with an ultraviolet (UV) detector and a C18 column (4.6 × 250 mm). Methanol/ultra-pure water (9/1, v/v) was used as the mobile phase. The samples (20 μL) were injected into a C18 separation column at a flow rate of 1 mL min^{-1}, column temperature of 35 °C, and UV detection wavelength of 275 nm. The inhibition ratio, or the adsorption capacity reduction ratio, was calculated as follows: sorption inhibition ratio = ($K_{P\text{-}ck}$ − $K_{P\text{-}eco\text{-}corona}$)/ $K_{P\text{-}ck}$, where K_p is the adsorption coefficient representing the steady-state concentration (C) ratio of sorbed to dissolved analyte (C_{sorbed}/C_{water}), $K_{P\text{-}ck}$ is the adsorption coefficient of DBP on microplastics in pure water, $K_{P\text{-}eco\text{-}corona}$ the adsorption coefficient of DBP sorption on eco-corona formed microplastics.

Text S5.

Data analysis. After UPLC-MS/MS analyses, the raw data were imported into the Progenesis QI 2.3 (Nonlinear Dynamics, Waters, USA) for peak detection and alignment. The preprocessing results generated a data matrix that consisted of the retention time (RT), mass-to-charge ratio (m/z) values, and peak intensity. Metabolic features detected at least 80% in any set of samples were retained. After filtering, minimum metabolite values were imputed for specific samples in which the metabolite levels fell below the lower limit of quantitation and each metabolic feature were normalized by sum. The internal standard was used for data QC (reproducibility), metabolic features which the relative standard deviation (RSD) of QC > 30% were discarded. Following normalization procedures and imputation, statistical analysis was performed on log transformed data to identify significant differences in metabolite levels between comparable groups. Mass spectra of these metabolic features were identified by using the accurate mass, MS/MS fragments spectra and isotope ratio difference with searching in reliable biochemical databases as Human metabolome database (HMDB) (http://www.hmdb.ca/) and Metlin database (https://metlin.scripps.edu/). A multivariate statistical analysis was performed using ropls (Version1.6.2, http://bioconductor.org/packages/release/bioc/html/ropls. html) Principle component analysis using an unsupervised method was applied to obtain an overview of the metabolic data, general clustering, trends, or outliers were visualized. The metabolite variables were scaled to unit-variances prior to conducting the principal component analysis (PCA). Orthogonal partial least squares discriminate analysis (OPLS-DA) was used for statistical analysis to determine global metabolic changes between comparable groups. All the metabolite variables were scaled to Pareto scaling prior to conducting the OPLS-DA. The model validity was evaluated from model parameters R^2 and Q^2, which provide information for the interpretability and predictability, respectively, of the model and avoid the risk of over-fitting. Variable importance in the projection (VIP) were calculated in OPLS-DA model. P values were estimated with paired Student's t-test on single dimensional statistical analysis. The metabolites with $VIP >$ 1 and $p \leq 0.05$ were metabolites with significant difference. The three types of soil metabolomes

without any microplastics were conducted to a 72 h oscillation as blank control. It was found that only 6 and 9 compounds showed an increase and decrease trends after oscillation in mollisol soil metabolomes, 9 and 9 in fluvo-aquic soil, and 4 and 5 in red soil, accounting to only 2.48%, 2.96%, and 1.49% of the total metabolite numbers, respectively. This indicates that the 72 h experiment did not significantly change the main metabolite composition in the absence of microorganisms and microplastics, and that the analysis of the effect of microplastics on soil metabolomes via non-targeted methods is reliable.

Table S1. Physico-chemical properties of soils.

	pH	OM ($g\ kg^{-1}$)	DOC ($mg\ kg^{-1}$)	TN ($g\ kg^{-1}$)	TP ($g\ kg^{-1}$)	TK ($g\ kg^{-1}$)	CEC ($cmol\ kg^{-1}$)	ES ($mg\ kg^{-1}$)	Sand (%)	Silt (%)	Clay (%)	Microplastics (%)
Mollisol soil	5.25	44.82	115.91	2.23	1.08	16.30	25.33	9.80	6.68	65.88	27.44	0.002%
Fluvo-aquic soil	8.52	9.67	47.73	0.61	0.72	16.81	8.34	15.90	65.78	27.69	6.53	0.0005%
Red soil	4.64	13.40	85.23	0.73	0.55	12.48	17.69	31.95	27.60	36.44	35.96	0.0006%

Note: OM: organic matter; DOC: dissolved organic carbon; CEC: cation exchange capacity; TN: total nitrogen; TP: total phosphorus; TK: total potassium; ES: effective sulfur.

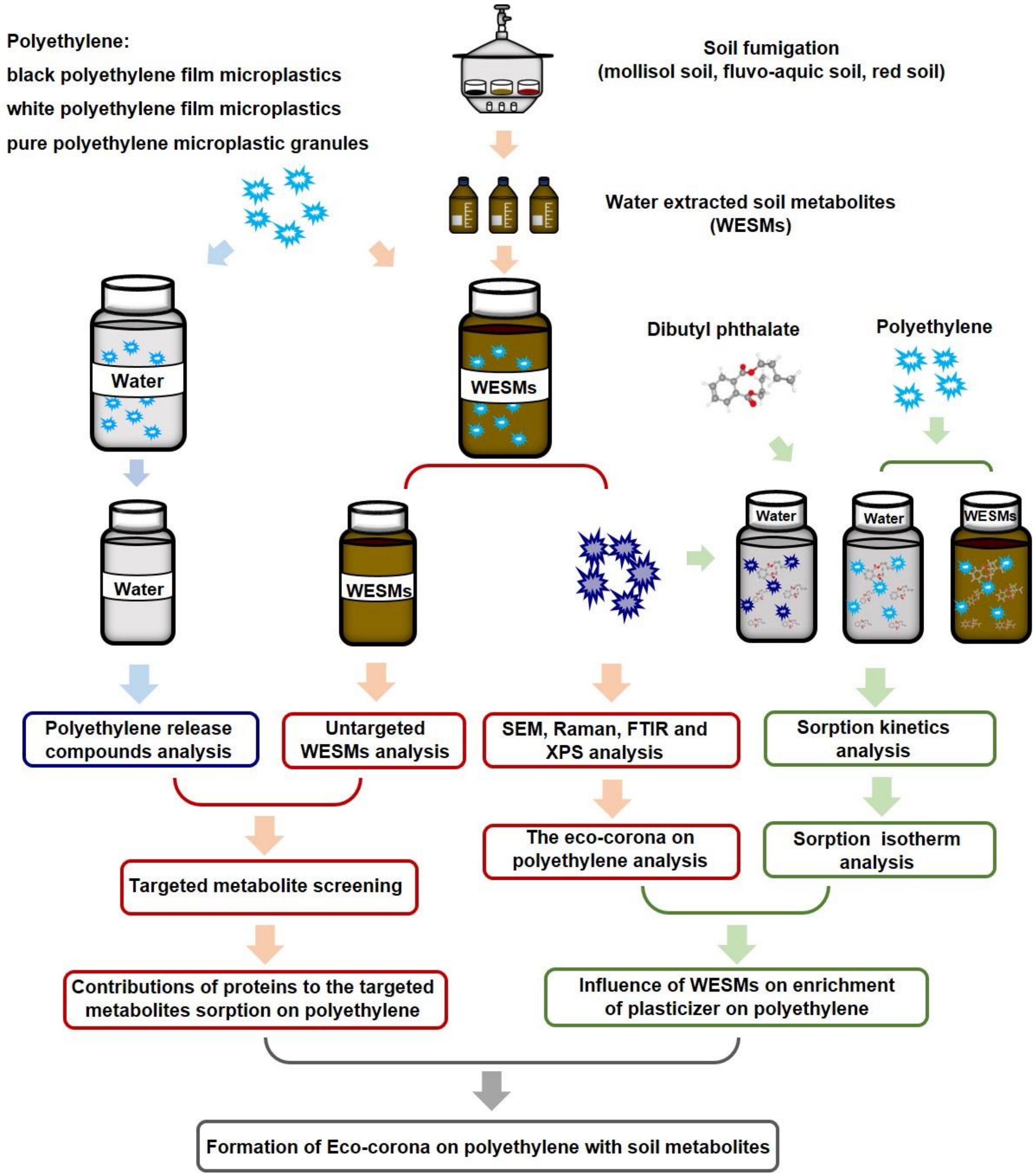

Figure S1. The experimental procedures of this study.

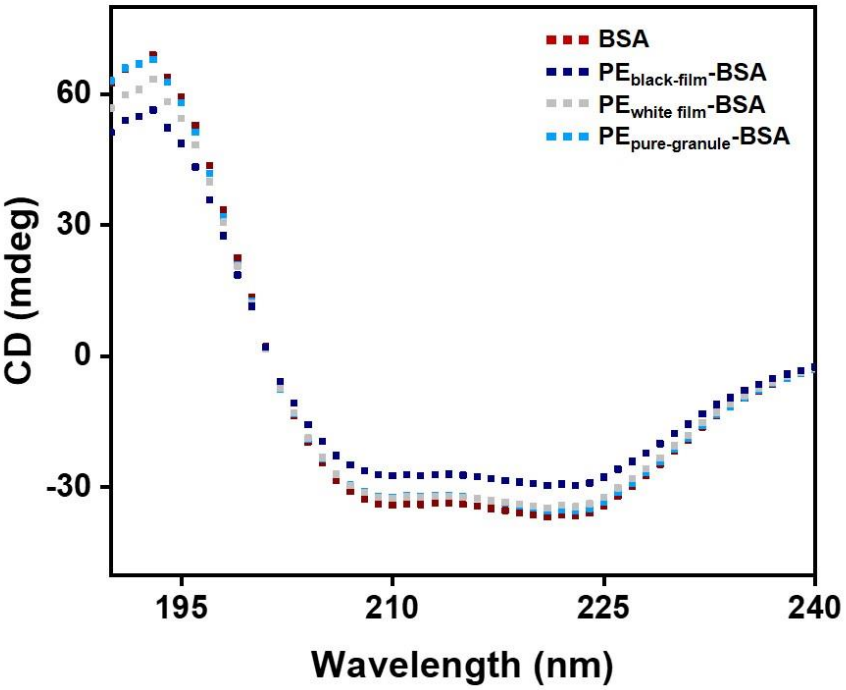

Figure S2. Circular dichroism (CD) spectra of the interaction between bovine serum albumin (BSA) and microplastics. $PE_{black-film}$: black polyethylene film microplastics; $PE_{white-film}$: white polyethylene film microplastics; $PE_{pure-granule}$: pure polyethylene microplastic granules. The protein structure did not change after exposure to microplastics.

Table S2. Kinetics models and isotherm models, equations and parameters for dibutyl phthalate sorption on microplastics.

	Models	Equations
Kinetics	Pesudo-first-order	$Q_t=Q_e(1-exp\ (-k_1t))$
	Pesudo-second-order	$Q_t=(k_2Q_e^2t)/(1+k_2Q_et)$
Isotherm	Linear	$Q_e=K_d\ C_e$
	Freundlich	$Q_e=K_FC_e^{1/n}$
Equilibrium partition ratio between DBP concentration in microplastics and background solution		$K_p=C_{sorbed}/C_{\mathrm{background\ solution}}$

Note: Q_t and Q_e are the sorption amount at time t and sorption equilibrium respectively (mg kg^{-1}); t is the sorption time (min); k_1 and k_2 are pseudo-first-order and pseudo-second-order rate constants, respectively, and the units are (min^{-1}) and (kg mg^{-1} min^{-1}), respectively. K_d and K_F are the Linear and Freundlich constant (L mg^{-1}), respectively; $1/n$ is the sorption affinity constant; K_p is partition coefficient.

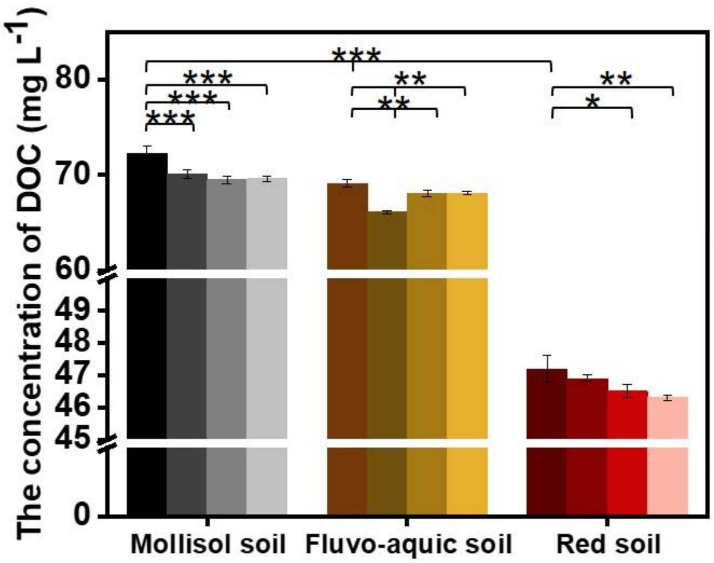

Figure S3. The concentration of dissolved organic matter (DOC) in different solutions of water extracted soil metabolites interacted with or without microplastics. The color depth of each group was in order of blank control, black polyethylene film microplastics, white polyethylene film microplastics and pure polyethylene microplastic granules sorption treatments. (***$p \leq 0.001$, **$p \leq 0.01$, *$p \leq 0.05$).

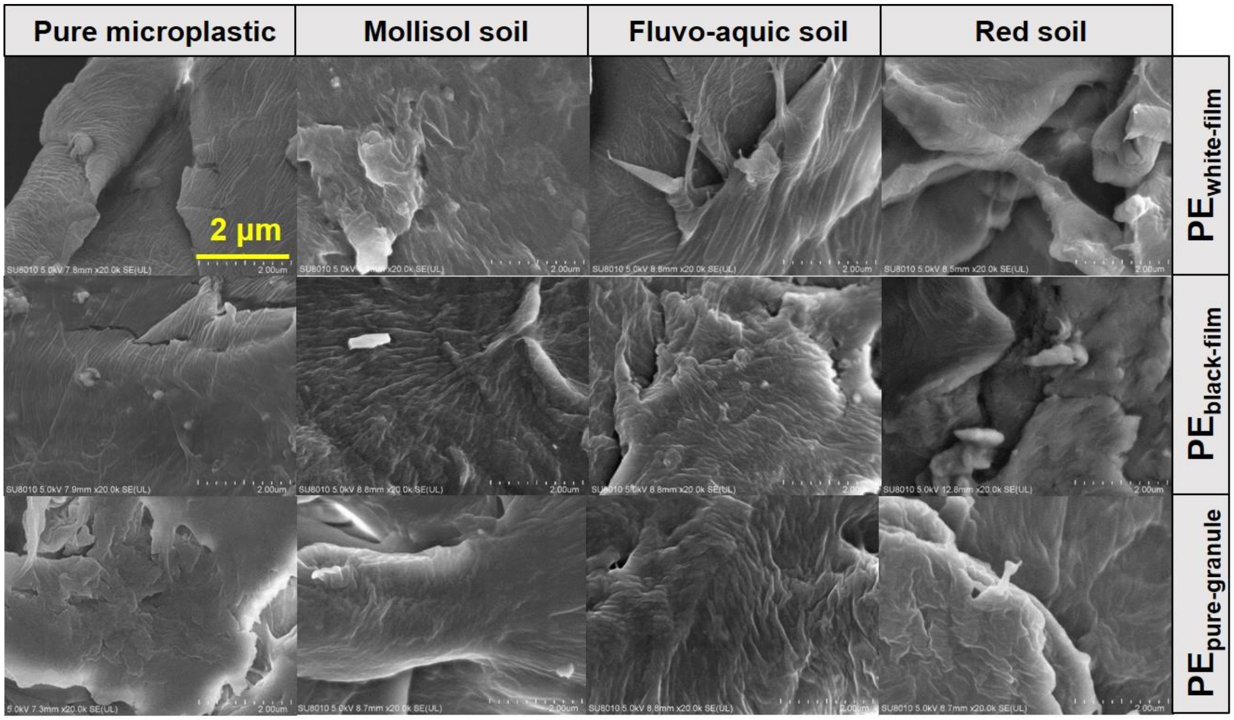

Figure S4. The scanning electron microscopy images of microplastics after interacting with water extracted soil metabolites from different soils or without (Pure microplastic). $PE_{black\text{-}film}$: black polyethylene film microplastics; $PE_{white\text{-}film}$: white polyethylene film microplastics; $PE_{pure\text{-}granule}$: pure polyethylene microplastic granules.

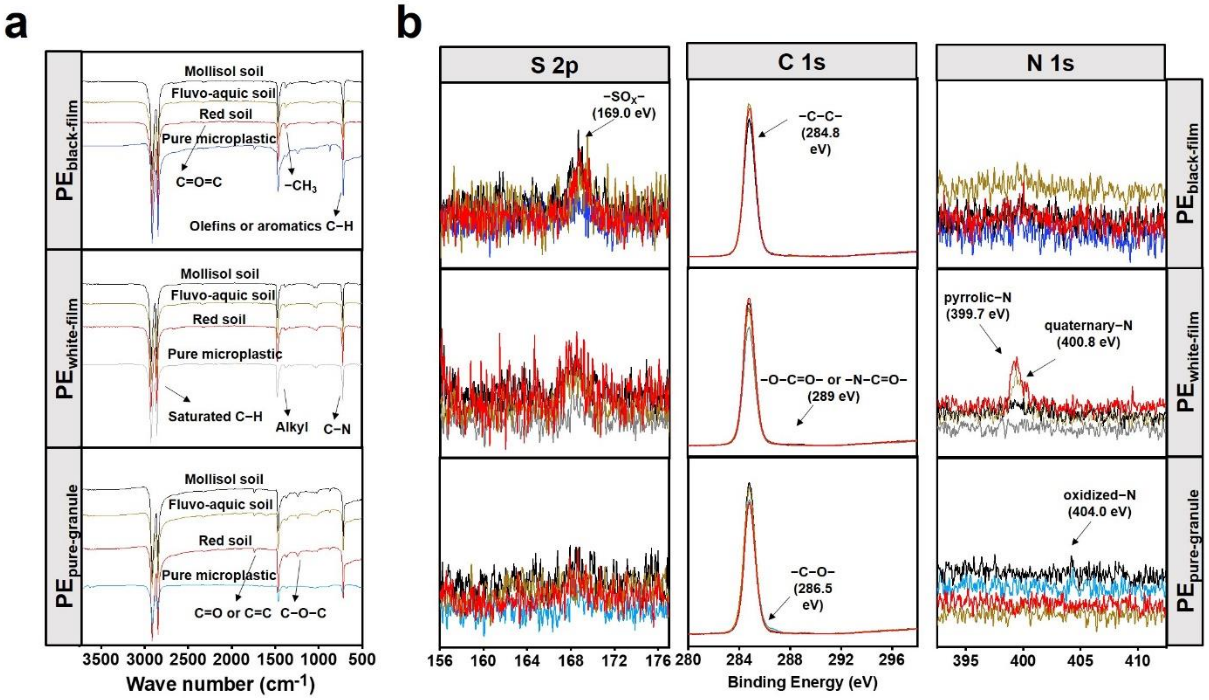

Figure S5. The fourier transform infrared spectroscopy (a) and X ray photoelectron spectroscopy (b) analysis of the coating of microplastic particles interacted with water extracted soil metabolites from different soils or without (Pure microplastic). $PE_{black\text{-}film}$: black polyethylene film microplastics; $PE_{white\text{-}film}$: white polyethylene film microplastics; $PE_{pure\text{-}granule}$: pure polyethylene microplastic granules.

Table S3. All identifiable soil metabolites.

Metabolite	Library ID	Mode	Formula	HMDB Superclass
Allysine	HMDB0001263	pos	C6H11NO3	Organic acids and derivatives
C9:2n-2,4	LMFA01030450	pos	C9H14O2	-
Pisumionoside	HMDB0039947	pos	C19H32O9	Lipids and lipid-like molecules
N6-Acetyl-5S-hydroxy-L-lysine	HMDB0033891	pos	C8H16N2O4	Organic acids and derivatives
Glu-Val	-	pos	C10H18N2O5	-
Sarmentosin	HMDB0030697	pos	C11H17NO7	Lipids and lipid-like molecules
Hypoxanthine	HMDB0000157	pos	C5H4N4O	Organoheterocyclic compounds
3-Isopropylcatechol	-	pos	C9H12O2	-
2,6,10,10-Tetramethyl-1-oxaspiro[4.5]decan-6-ol	HMDB0037907	pos	C13H24O2	Organoheterocyclic compounds
Cis-5-dodecenoic acid	-	pos	C12H22O2	-
Propyl 2,4-decadienoate	HMDB0037307;LMFA07011001	pos	C13H22O2	Lipids and lipid-like molecules
Monic acid	HMDB0061154	pos	C18H30O6	Lipids and lipid-like molecules
Mangiferic acid	HMDB0029800	pos	C18H32O2	Lipids and lipid-like molecules
3,6,7-Trihydroxy-4'-methoxyflavone 7-rhamnoside	LMPK12111586;HMDB0041455	pos	C22H22O10	Phenylpropanoids and polyketides
Sucrose	HMDB0000258	pos	C12H22O11	Organic oxygen compounds
2-Hydroxycinnamic acid	HMDB0002641	pos	C9H8O3	Phenylpropanoids and polyketides
4-Guanidinobutanoic acid	HMDB0003464	pos	C5H11N3O2	Organic acids and derivatives
(-)-erythro-Anethole glycol 1-glucoside	HMDB0033066	pos	C16H24O8	Organic oxygen compounds
Cibaric acid	HMDB0038580;LMFA02000289	pos	C18H28O5	Lipids and lipid-like molecules
[6]-Gingerdiol 3-acetate	HMDB0040566	pos	C19H30O5	Lipids and lipid-like molecules
N-stearoyl valine	LMFA08020122	pos	C23H45NO3	-
Bis(2-ethylhexyl) phthalate	-	pos	C24H38O4	-
Farnesyl acetone	-	pos	C18H30O	-
4-Isopropyl-3-cyclohexene-1-carboxylic acid	HMDB0037175	pos	C10H16O2	Lipids and lipid-like molecules
Sorbitan laurate	LMFA07011017;HMDB0029885	pos	C18H34O6	Lipids and lipid-like molecules
DDA	-	pos	C10H16O	-
Isopropyl apiosylglucoside	HMDB0041513	pos	C14H26O10	Organic oxygen compounds
Heptaethylene glycol	HMDB0061835	pos	C14H30O8	Organic oxygen compounds

Octaethylene glycol	HMDB0094680	pos	C16H34O9	Organic oxygen compounds
Genipinic acid	HMDB0038126	pos	C11H14O6	Organic acids and derivatives
Aloesol	HMDB0035712	pos	C13H14O4	Organoheterocyclic compounds
Betaine	HMDB0000043	pos	C5H11NO2	Organic acids and derivatives
3-(4-Isopropylphenyl)propanal	HMDB0036171	pos	C12H16O	Lipids and lipid-like molecules
1-Kestose	HMDB0011729	pos	C18H32O16	Organic oxygen compounds
L-Glutamic acid	HMDB0000148	pos	C5H9NO4	Organic acids and derivatives
1-[2-(2H-1,3-benzodioxol-5-yl)-1-benzofuran-5-yl]propane-1,3-diol	HMDB0131335	pos	C18H16O5	-
Aminohydroquinone	-	pos	C6H7NO2	-
Methionyl-Proline	HMDB0028981	pos	C10H18N2O3S	Organic acids and derivatives
N-(1-Deoxy-1-fructosyl)leucine	HMDB0037840	pos	C12H23NO7	Organic acids and derivatives
Pantothenic Acid	HMDB0000210	pos	C9H17NO5	Organooxygen compounds
M-Cresol	HMDB0002048	pos	C7H8O	Benzenoids
Marmeline	HMDB0030192	pos	C22H25NO3	Benzenoids
L-3-Cyanoalanine	HMDB0060245	pos	C4H6N2O2	Organic acids and derivatives
1-ACETYLPIPERIDINE	-	pos	C7H13NO	-
Chenodeoxycholic acid sulfate	LMST05020026;HMDB0002522	pos	C24H40O7S	Lipids and lipid-like molecules
PC(16:0/0:0)[U]	-	pos	C24H50NO7P	-
Alpha-Bisabolol oxide C	HMDB0038138	pos	C15H26O2	Organoheterocyclic compounds
4-Formylsalicylic acid	-	pos	C8H6O4	-
TG(16:1(9Z)/18:0/18:2(9Z,12Z))	HMDB0005425	pos	C55H100O6	Lipids and lipid-like molecules
TG(16:1(9Z)/18:0/18:1(9Z))	HMDB0005423	pos	C55H102O6	Lipids and lipid-like molecules
TG(18:0/18:1(9Z)/18:2(9Z,12Z))	HMDB0005405	pos	C57H104O6	Lipids and lipid-like molecules
TG(i-15:0/a-17:0/i-18:0)[rac]	HMDB0102236	pos	C53H102O6	Lipids and lipid-like molecules
6-Methoxymellein	HMDB0038510	pos	C11H12O4	Organoheterocyclic compounds
Geranylcitronellol	HMDB0032147	pos	C20H36O	Lipids and lipid-like molecules
Docosatrienoic acid	HMDB0002823	pos	C22H38O2	Lipids and lipid-like molecules
Riboflavin (Vitamin B2)	-	pos	C17H20N4O6	-
3-(4-hydroxyphenyl)-N-(4-oxobutyl)prop-2-enimidic acid	HMDB0139876	pos	C13H15NO3	Phenylpropanoids and polyketides
Cis-2-Carboxycyclohexyl-acetic acid	-	pos	C9H14O4	-
Cinnamic acid	HMDB0000567	pos	C9H8O2	Phenylpropanoids and polyketides
Thymine	HMDB0000262	pos	C5H6N2O2	Organoheterocyclic compounds
3,4-Methylenesebacic acid	HMDB0059729	pos	C12H18O4	Lipids and lipid-like molecules

D-Pantethine	HMDB0003828	pos	C22H42N4O8S2	Organic acids and derivatives
5-Hydroxyindoleacetic acid	HMDB0000763	pos	C10H9NO3	Organoheterocyclic compounds
5-Methoxy-DL-tryptophan	-	pos	C12H14N2O3	-
2,3-Butanediol glucoside	HMDB0040822	pos	C10H20O7	Organic oxygen compounds
Anthranilic acid	-	pos	C7H7NO2	-
Helenalin	-	pos	C15H18O4	-
N-Acetyl-DL-tryptophan	-	pos	C13H14N2O3	-
L-Homotyrosine	-	pos	C10H13NO3	-
8,8-Diethoxy-2,6-dimethyl-2-octanol	HMDB0034557	pos	C14H30O3	Organic oxygen compounds
N-Acetylmuramate	HMDB0060493	pos	C11H19NO8	Organic oxygen compounds
PPOH	-	pos	C15H18O3	-
13-amino-tridecanoic acid	LMFA01100006	pos	C13H27NO2	-
Crispolide	HMDB0036695	pos	C15H20O5	Lipids and lipid-like molecules
Melicopicine	-	pos	C18H19NO5	-
Procurcumadiol	HMDB0034721	pos	C15H22O3	Lipids and lipid-like molecules
Tanacetin	HMDB0035715	pos	C15H20O4	Lipids and lipid-like molecules
(R)-11,12,13-Trinor-1(5),6,9-guaiatrien-8-one	HMDB0039153	pos	C12H14O	Hydrocarbon derivatives
Preisocalamendiol	HMDB0035391	pos	C15H24O	Lipids and lipid-like molecules
Valerenolic acid	HMDB0036563	pos	C15H22O3	Lipids and lipid-like molecules
Nigellic acid	HMDB0036094	pos	C15H20O5	Lipids and lipid-like molecules
Cuminaldehyde	HMDB0002214	pos	C10H12O	Lipids and lipid-like molecules
9Z,13-Tetradecadien-11-ynal	LMFA06000180	pos	C14H20O	-
Caryophyllene epoxide	-	pos	C15H24O	-
Alpha-Carissanol	HMDB0035597	pos	C15H24O3	Lipids and lipid-like molecules
(3S,5R,6R,7E)-3,5,6-Trihydroxy-7-megastigmen-9-one	HMDB0038736	pos	C13H22O4	-
6,8,10,12-pentadecatetraenal	LMFA06000087	pos	C15H22O	-
12S-HHT	HMDB0012535	pos	C17H28O3	Lipids and lipid-like molecules
9,12,13-TriHOME	HMDB0004708;LMFA02000014	pos	C18H34O5	Lipids and lipid-like molecules
Longicamphenylone	-	pos	C14H22O	-
Thromboxane B3	LMFA03030006;HMDB0005099;LMFA03030016	pos	C20H32O6	Lipids and lipid-like molecules
METOLACHLOR	-	pos	C15H22ClNO2	-
Glycylprolylhydroxyproline	HMDB0002171	pos	C12H19N3O5	Organic acids and derivatives

6-[5]-ladderane-1-hexanol	LMFA05000063	pos	C18H28O	-
N,N-dimethyl-Safingol	LMSP01080056	pos	C20H43NO2	-
9-hydroxy-10-oxo-12(Z)-octadecenoic acid	-	pos	C18H32O4	-
2-(5,8-Tetradecadienyl)cyclobutanone	HMDB0037519	pos	C18H30O	Organooxygen compounds
18alpha-Hydroxyglycyrrhetic acid	HMDB0040499	pos	C30H46O5	Lipids and lipid-like molecules
Phenylacetaldehyde	HMDB0006236	pos	C8H8O	Benzenoids
10-Oxo-11-octadecen-13-olide	HMDB0029786	pos	C18H30O3	Phenylpropanoids and polyketides
4-Hydroxybenzaldehyde	HMDB0011718	pos	C7H6O2	Organic oxygen compounds
L-Isoleucine	HMDB0000172	pos	C6H13NO2	Organic acids and derivatives
Guanine	HMDB0000132	pos	C5H5N5O	Organoheterocyclic compounds
N-Acetylmannosamine	HMDB0001129	pos	C8H15NO6	Organic oxygen compounds
N-(6-aminohexanoyl)-6-aminohexanoic acid	LMFA00000009	pos	C12H24N2O3	-
2-{3-[(3,3-dimethyloxiran-2-yl)methyl]-4,6-dihydroxy-2-methoxyphenyl}acetic acid	HMDB0125462	pos	C14H18O6	Benzenoids
Isopropyl beta-D-glucoside	HMDB0032705	pos	C9H18O6	Organic oxygen compounds
5-Aminopentanamide	HMDB0012176	pos	C5H12N2O	Organic acids and derivatives
5,7-Megastigmadien-9-ol glucoside	HMDB0041044	pos	C19H32O6	Lipids and lipid-like molecules
2-Acetyl-1,5,6,7-tetrahydro-6-hydroxy-7-(hydroxymethyl)-4H-azepine-4-one	HMDB0035177	pos	C9H13NO4	Organoheterocyclic compounds
2-(14,15-Epoxyeicosatrienoyl) Glycerol	HMDB0013651	pos	C23H38O5	Lipids and lipid-like molecules
Glutaminylarginine	HMDB0028791	pos	C11H22N6O4	Organic acids and derivatives
Triacanthine	-	pos	C10H13N5	-
Quinceoxepine	HMDB0038107	pos	C12H18O	Organooxygen compounds
INDOLE-3-CARBINOL	HMDB0005785	pos	C9H9NO	Organoheterocyclic compounds
Galactosylhydroxylysine	HMDB0000600	pos	C12H24N2O8	Organic acids and derivatives
()-Ibuprofen	-	pos	C13H18O2	-
2-AI	-	pos	C9H11N	-
Brefeldin A	-	pos	C16H24O4	-
3b-Hydroxy-6b-methoxy-7(11)-eremophilen-12,8a-olide	HMDB0040755	pos	C16H24O4	Lipids and lipid-like molecules
2-hydroxyphenylpropionylglycine	HMDB0094723	pos	C11H13NO4	Organic acids and derivatives
1,N2-propanodeoxyguanosine	HMDB0059780	pos	C13H17N5O4	Nucleosides, nucleotides, and analogues
C12:5n-1,3,5,7,9	LMFA01030243	pos	C12H14O2	-
MG(15:0/0:0/0:0)	HMDB0011563	pos	C18H36O4	Lipids and lipid-like molecules
6-Hydroxyhexadecanoic acid	HMDB0112192	pos	C16H32O3	Lipids and lipid-like molecules

Phenylalanyl-Arginine	HMDB0028989	pos	C15H23N5O3	Organic acids and derivatives
3-Hydroxy-beta-ionone	HMDB0036821	pos	C13H20O2	Lipids and lipid-like molecules
Cinnamyl formate	HMDB0040577	pos	C10H10O2	Benzenoids
(+)-Abscisic Acid	-	pos	C15H20O4	-
Hydroxyibuprofen	-	pos	C13H18O3	-
Citreoviridin C	HMDB0030523	pos	C23H30O6	Organoheterocyclic compounds
19-Nor-5-androstenediol	HMDB0004590;LMST 02010050	pos	C18H28O2	Lipids and lipid-like molecules
4,11,13,15-Tetrahydroridentin B	HMDB0036150	pos	C15H24O4	Lipids and lipid-like molecules
Mono-(2-ethyl-5-oxohexyl) phthalate	HMDB0094645	pos	C16H20O5	Benzenoids
Chavicol	HMDB0034107	pos	C9H10O	Benzenoids
2,3-Dinor-TXB2	LMFA03030003;HMD B0002904	pos	C18H30O6	Lipids and lipid-like molecules
(3beta,6beta)-Furanoeremophilane-3,6-diol 6-acetate	HMDB0034930	pos	C17H24O4	Lipids and lipid-like molecules
Pentadecanoic acid	LMFA01010015;HMD B0000826	pos	C15H30O2	Lipids and lipid-like molecules
8-Deoxy-11-hydroxy-13-chlorogrosheimin	HMDB0041037	pos	C15H19ClO4	Lipids and lipid-like molecules
5-Phenyl-1-pentanol	HMDB0031624	pos	C11H16O	Lipids and lipid-like molecules
2,5-Dimethylbenzaldehyde	-	pos	C9H10O	-
8-Oxohexadecanoic acid	HMDB0030972	pos	C16H30O3	Lipids and lipid-like molecules
Sphingosine	LMSP01010001;HMD B0000252	pos	C18H37NO2	Organic nitrogen compounds
Nonadecanoic acid	LMFA01010019;HMD B0000772	pos	C19H38O2	Lipids and lipid-like molecules
2-Hydroxy-2,6,6-trimethylcyclohexanone	HMDB0037023	pos	C9H16O2	Organic oxygen compounds
12-Oxo-20-carboxy-leukotriene B4	HMDB0012550;LMFA 03020047	pos	C20H28O6	Lipids and lipid-like molecules
5-Hexyl-2-furanhexanoic acid	HMDB0112088	pos	C16H26O3	Lipids and lipid-like molecules
Palmitoleoyl Ethanolamide	HMDB0013648	pos	C18H35NO2	Organic nitrogen compounds
Glyceryl 5-hydroxydecanoate	HMDB0032297	pos	C13H26O5	Lipids and lipid-like molecules
(2xi,3xi,6E)-3,7-Dimethyl-6-octene-1,2,3,8-tetrol	HMDB0033642	pos	C10H20O4	Lipids and lipid-like molecules
Cerebronic acid	HMDB0039540	pos	C24H48O3	Lipids and lipid-like molecules
3-Hydroxy-4-phenylbutan-2-one	HMDB0032336	pos	C10H12O2	Benzenoids
Hericene B	HMDB0041180	pos	C37H58O5	Lipids and lipid-like molecules
1-Undecanol	HMDB0013113	pos	C11H24O	Lipids and lipid-like molecules
N1,N5,N10-Tris-trans-p-	HMDB0039962	pos	C37H44N4O6	Phenylpropanoids and polyketides

coumaroylspermine				
Palmitic amide	HMDB0012273	pos	C16H33NO	Lipids and lipid-like molecules
D6-Ambrettolide	HMDB0037805	pos	C16H28O2	Phenylpropanoids and polyketides
Stearoylethanolamide	HMDB0013078	pos	C20H41NO2	Organic nitrogen compounds
Ganoderic acid beta	HMDB0033234	pos	C30H44O6	Lipids and lipid-like molecules
N,N,N-trimethyl-sphingosine	LMSP01080057	pos	C21H43NO2	-
2-Heptadecylfuran	HMDB0033608	pos	C21H38O	Organoheterocyclic compounds
Oleragenoside	HMDB0036943	pos	C42H64O16	Lipids and lipid-like molecules
Aniline	HMDB0003012	pos	C6H7N	Benzenoids
Dimethylethanolamine	HMDB0032231	pos	C4H11NO	Organonitrogen compounds
N-Desmethyldiltiazem	HMDB0061023	pos	C21H24N2O4S	Organoheterocyclic compounds
Triphenyl phosphate	-	pos	C18H15O4P	-
DG(11D3/13D5/0:0)	HMDB0116373	pos	C47H80O7	Lipids and lipid-like molecules
All-trans-Retinoic acid	HMDB0001852	pos	C20H28O2	Lipids and lipid-like molecules
Deacetylnomilin	HMDB0035684	pos	C26H32O8	Lipids and lipid-like molecules
1-Phenylheptane	HMDB0061825	pos	C13H20	Benzenoids
Austalide J	HMDB0030156	pos	C25H32O7	Organoheterocyclic compounds
PG(20:4(5Z,8Z,11Z,14Z)/0:0)	LMGP04050010	pos	C26H45O9P	-
Octadecanol	LMFA05000085;HMDB0002350	pos	C18H38O	Lipids and lipid-like molecules
Vulgarone A	HMDB0036733	pos	C15H22O	Lipids and lipid-like molecules
Germacrenone	HMDB0035887	pos	C15H26O2	Lipids and lipid-like molecules
Sphinganine	HMDB0000269;LMSP01020001	pos	C18H39NO2	Organic nitrogen compounds
2,4,12-Octadecatrienoic acid isobutylamide	HMDB0032033	pos	C22H39NO	Lipids and lipid-like molecules
4,5-Dihydrovomifoliol	HMDB0040615	pos	C13H22O3	Lipids and lipid-like molecules
Diisobutyl adipate	LMFA07010834;HMDB0041618	pos	C14H26O4	Lipids and lipid-like molecules
Muzanzagenin	HMDB0032601	pos	C27H38O5	Lipids and lipid-like molecules
Propylene glycol mono- and diesters of fats and fatty acids	LMFA07011007;HMDB0032494	pos	C32H62O4	Lipids and lipid-like molecules
Palmitoyl Ethanolamide	LMFA08040057;LMFA08040013	pos	C18H37NO2	-
Phytosphingosine	HMDB0004610;LMSP01030001	pos	C18H39NO3	Organic nitrogen compounds
3-Methyl-5-propyl-2-furanundecanoic acid	HMDB0061646	pos	C19H32O3	Lipids and lipid-like molecules
Ankorine	-	pos	C19H29NO4	-

Tricosanoic acid	LMFA01010023;HMDB0001160	pos	C23H46O2	Lipids and lipid-like molecules
2-amino-14,16-dimethyloctadecan-3-ol	LMSP01080031	pos	C20H43NO	-
Eicosanoyl-EA	LMFA08040038	pos	C22H45NO2	-
Prosopinine	LMSP01080049	pos	C16H33NO3	-
(1R,2R,4S)-p-Menthane-1,2,8-triol 8-glucoside	HMDB0039894	pos	C16H30O8	Organic oxygen compounds
LysoSM(d18:0)	HMDB0012082	pos	C23H51N2O5P	Lipids and lipid-like molecules
Dihydromonacolin L	-	pos	C19H30O3	-
Heneicosanoic acid	HMDB0002345;LMFA01010021	pos	C21H42O2	Lipids and lipid-like molecules
N,N,O-Tridesmethyltramadol	HMDB0060850	pos	C13H19NO2	Benzenoids
(+)-4,11-Eudesmadien-3-one	HMDB0037061	pos	C15H22O	Lipids and lipid-like molecules
(+)-3,7(11)-Acoradiene	LMPR0103660002	pos	C15H22O	-
N-(14-Methylhexadecanoyl)pyrrolidine	HMDB0034373	pos	C21H41NO	Organoheterocyclic compounds
Spisulosine	LMSP01080032	pos	C18H39NO	-
D-erythro-Sphingosine C-20	-	pos	C20H41NO2	-
Allyl benzoate	HMDB0040592	pos	C10H10O2	Benzenoids
MG(a-21:0/0:0/0:0)[rac]	HMDB0072855	pos	C24H48O4	Lipids and lipid-like molecules
9,10-epoxy-12-octadecenoic acid	LMFA02000280	pos	C18H32O3	-
Dolicholide	HMDB0034086	pos	C28H46O6	Lipids and lipid-like molecules
10,20-Dihydroxyeicosanoic acid	HMDB0031923	pos	C20H40O4	Lipids and lipid-like molecules
Mutisianthol	LMPR0103360001	pos	C15H20O	-
Polyoxyethylene 40 monostearate	HMDB0032477	pos	C20H40O3	Lipids and lipid-like molecules
MG(19:0/0:0/0:0)	HMDB0072839	pos	C22H44O4	Lipids and lipid-like molecules
C16 Sphingosine	LMSP01040008	pos	C16H33NO2	-
Lucidenolactone	HMDB0030897	pos	C27H36O6	Lipids and lipid-like molecules
25-Hydroxyvitamin D3-26,23-lactone	HMDB0060126	pos	C27H40O4	Lipids and lipid-like molecules
Austalide L	HMDB0030158	pos	C25H32O6	Phenylpropanoids and polyketides
Pectachol	HMDB0039064	pos	C26H34O6	Phenylpropanoids and polyketides
Ethyl salicylate	HMDB0029817	pos	C9H10O3	Benzenoids
Avocadene	HMDB0031042;LMFA05000639	pos	C17H34O3	Lipids and lipid-like molecules
Pyruvophenone	-	pos	C9H8O2	-
Ricinoleic acid	LMFA02000184;HMDB0034297	pos	C18H34O3	Lipids and lipid-like molecules
Secoeremopetasitolide A	HMDB0041364	pos	C19H26O7	Lipids and lipid-like molecules

Oblongolide	-	pos	C14H20O2	-
(6b,7b,13R)-6,7-Diacetoxy-8,14-labdadiene-13-ol	HMDB0035288	pos	C24H38O5	Lipids and lipid-like molecules
Solasodine	LMST01150004;HMDB0035282	pos	C27H43NO2	Lipids and lipid-like molecules
Heptadecanoic acid	HMDB0002259;LMFA01010017	pos	C17H34O2	Lipids and lipid-like molecules
Xestoaminol C	LMSP01080033	pos	C14H31NO	-
Isokobusone	HMDB0036791	pos	C14H22O2	Organic oxygen compounds
4E,7Z,10Z-Tridecatrienyl acetate	LMFA07010271	pos	C15H24O2	-
(1S,2R,4R)-p-Menth-8-ene-2,10-diol 2-glucoside	HMDB0039055	pos	C16H28O7	Lipids and lipid-like molecules
(S)-11,12,13-Trinor-7-calamenone	HMDB0040823	pos	C12H14O	Benzenoids
13(S)-HOTrE	-	pos	C18H30O3	-
Corchorifatty acid D	HMDB0033243	pos	C18H28O4	Lipids and lipid-like molecules
Beta-Ionol	HMDB0036820	pos	C13H22O	Lipids and lipid-like molecules
Lycoramine	-	pos	C17H23NO3	-
Blumenol C O-[apiosyl-(1->6)-glucoside]	HMDB0031936	pos	C24H40O11	Lipids and lipid-like molecules
Capsaicin	HMDB0002227;LMFA08020085	pos	C18H27NO3	Benzenoids
Kessyl glycol	HMDB0037197	pos	C15H26O3	Organoheterocyclic compounds
Isoalantolactone	HMDB0035934	pos	C15H20O2	Lipids and lipid-like molecules
6-Keto-prostaglandin F1a	HMDB0002886	pos	C20H34O6	Lipids and lipid-like molecules
(4E,d14:1) sphingosine	LMSP01040006	pos	C14H29NO2	-
Corey PG-Lactone Diol	-	pos	C15H24O4	-
Floionolic acid	LMFA02000147;HMDB0034295	pos	C18H36O5	Lipids and lipid-like molecules
(R)-1,3-Octanediol	HMDB0029359;LMFA05000564	pos	C8H18O2	Lipids and lipid-like molecules
2'-Deoxymugineic acid	HMDB0033909	pos	C12H20N2O7	Organic acids and derivatives
Alanyl-Gamma-glutamate	HMDB0028701	pos	C8H15N3O4	Organic acids and derivatives
Osmaronin	HMDB0032769	pos	C11H17NO6	Lipids and lipid-like molecules
Heptane-1-thiol	HMDB0032304	pos	C7H16S	Organosulfur compounds
3-hydroxydecanoyl carnitine	HMDB0061636	pos	C17H33NO5	Lipids and lipid-like molecules
3,4-Dihydroxyphenylvaleric acid	HMDB0029233	pos	C11H14O4	Benzenoids
Gamma-Caprolactone	LMFA07040010;HMDB0003843	pos	C6H10O2	Organoheterocyclic compounds
Dicyclohexyl disulfide	HMDB0041448	pos	C12H22S2	Organosulfur compounds

Methyl 3-(2,3-dihydroxy-3-methylbutyl)-4-hydroxybenzoate	HMDB0032796	pos	C13H18O5	Benzenoids
Tanacetol A	HMDB0035722	pos	C17H26O4	Lipids and lipid-like molecules
Phaseic acid	-	pos	C15H20O5	-
Lauroyl diethanolamide	LMFA08040058;HMDB0032358	pos	C16H33NO3	Lipids and lipid-like molecules
2-(3-Hydroxyphenyl)ethanol 1'-glucoside	HMDB0038332	pos	C14H20O7	Organic oxygen compounds
12-Hydroxydodecanoic acid	HMDB0002059	pos	C12H24O3	Organic acids and derivatives
N-(2-Phenylethyl)-acetamide	-	pos	C10H13NO	-
16-Hydroxy hexadecanoic acid	HMDB0006294	pos	C16H32O3	Lipids and lipid-like molecules
2-Diethylaminoethanol	HMDB0033971	pos	C6H15NO	Organic nitrogen compounds
Indole-3-ethanol	-	pos	C10H11NO	-
(S)-10,16-Dihydroxyhexadecanoic acid	HMDB0037798	pos	C16H32O4	Lipids and lipid-like molecules
Vanillic acid-4-O-glucuronide	HMDB0060024	pos	C15H18O9	Organic oxygen compounds
Carvotanacetone	HMDB0059875	pos	C10H16O	Lipids and lipid-like molecules
3,4,5,6-Tetrahydrohippuric acid	HMDB0061679	pos	C9H13NO3	Organic acids and derivatives
Tricyclazole	HMDB0031809	pos	C9H7N3S	Organoheterocyclic compounds
Dihydrophaseic acid	LMPR0103050005;HMDB0038660	pos	C15H22O5	Lipids and lipid-like molecules
4-formyl Indole	-	pos	C9H7NO	-
5-(3E-Pentenyl)tetrahydro-2-oxo-3-furancarboxylic acid	HMDB0030991	pos	C10H14O4	Organoheterocyclic compounds
Solanolone	HMDB0033262	pos	C15H18O5	Benzenoids
1-H-Inden-1-one,2,3-dihydro-3,3,5,6-tetramethyl	HMDB0059683	pos	C13H16O	Benzenoids
Benzenemethanol, 2-(2-hydroxypropoxy)-3-methyl-	-	pos	C11H16O3	-
3,7,8,15-Scirpenetetrol	HMDB0037560	pos	C15H22O6	Lipids and lipid-like molecules
KAPA	-	pos	C9H17NO3	-
7-Aminonitrazepam	HMDB0041819	pos	C15H13N3O	Organoheterocyclic compounds
Cis-1,3,4,6,7,11b-Hexahydro-9-methoxy-2H-benzo[a]quinolizine-3-carboxylic acid	-	pos	C15H19NO3	-
2-ISOPROPYL-3-METHOXYCINNAMIC ACID	-	pos	C13H16O3	-
(R)-3,7-Dimethyl-5-indanecarboxylic acid	HMDB0033033	pos	C12H14O2	Benzenoids
2,6-Dimethyl-4-hydroxybenzaldehyde	-	pos	C9H10O2	-
(E)-2-Butenyl-4-methyl-threonine	-	pos	C9H17NO3	-
Tenuazonic acid	HMDB0036074	pos	C10H15NO3	Organoheterocyclic compounds

Dioscoretine	HMDB0038588	pos	C13H23NO3	Lipids and lipid-like molecules
Sebiferic acid	HMDB0036774	pos	C30H48O2	Lipids and lipid-like molecules
L-2-Amino-3-(4-aminophenyl)propanoic acid	HMDB0030397	pos	C9H12N2O2	Phenylpropanoids and polyketides
Isopentenyladenine-9-N-glucoside	HMDB0012240	pos	C17H25N5O4	Organic oxygen compounds
Trp-P-1	-	pos	C13H13N3	-
Glucosyl 6-hydroxy-2,6-dimethyl-2E,7-octadienoate	HMDB0035822	pos	C16H26O8	Organic oxygen compounds
Margaroylglycine	HMDB0013246	pos	C19H37NO3	Organic acids and derivatives
9-Fluoro-16alpha-hydroxyandrost-4-ene-3,11,17-trione	-	pos	C19H23FO4	-
2-Amino-2-Norbornanecarboxylic acid	-	pos	C8H13NO2	-
1-[(5-Amino-5-carboxypentyl)amino]-1-deoxyfructose	HMDB0034879	pos	C12H24N2O7	Organic oxygen compounds
Leukotriene E3	LMFA03020074;HMDB0002355	pos	C23H39NO5S	Lipids and lipid-like molecules
Ethyl (S)-3-hydroxybutyrate glucoside	HMDB0031693	pos	C12H22O8	Lipids and lipid-like molecules
1,2,10-Trihydroxydihydro-trans-linalyl oxide 7-O-beta-D-glucopyranoside	HMDB0033237	pos	C16H30O10	Organic oxygen compounds
Kynuramine	HMDB0012246	pos	C9H12N2O	Organic oxygen compounds
Dolichodial	LMPR0102070037	pos	C10H14O2	-
Carbendazim	HMDB0031769	pos	C9H9N3O2	Organoheterocyclic compounds
ALANYL-dl-PHENYLALANINE	-	pos	C12H16N2O3	-
N5-(4-Methoxybenzyl)glutamine	HMDB0033598	pos	C13H18N2O4	Organic acids and derivatives
N-Acetylmuramoyl-Ala	HMDB0060494	pos	C14H24N2O9	Organic oxygen compounds
Isoleucyl-Hydroxyproline	HMDB0028908	pos	C11H20N2O4	Organic acids and derivatives
Aminocaproic acid	HMDB0001901	pos	C6H13NO2	Lipids and lipid-like molecules
Dexpanthenol	-	pos	C9H19NO4	-
8-Hydroxyoctanoate	HMDB0061914	pos	C8H16O3	Organic compounds
Benzaldehyde	HMDB0006115	pos	C7H6O	Benzenoids
ALANYL-dl-LEUCINE	-	pos	C9H18N2O3	-
2,3-Dinor-6-keto-prostaglandin F1 a	HMDB0002277	pos	C18H30O6	Lipids and lipid-like molecules
Alitame	HMDB0037324	pos	C14H25N3O4S	Organic acids and derivatives
EHNA	-	pos	C14H23N5O	-
5-Aminopentanal	HMDB0012815	pos	C5H11NO	Organic oxygen compounds
2-Pyrrolidinone	HMDB0002039	pos	C4H7NO	Organoheterocyclic compounds
5'-Dehydroadenosine	-	pos	C10H11N5O4	-

5-(hydroxymethyl)- 2-Furancarboxylic acid	-	pos	C6H6O4	-
N-Acetyl-4-O-acetylneuraminic acid	HMDB0000796	pos	C13H21NO10	Organic oxygen compounds
Deoxyadenosine	HMDB0000101	pos	C10H13N5O3	Nucleosides, nucleotides, and analogues
Beta-Alaninamide	-	pos	C3H8N2O	-
Threoninyl-Hydroxyproline	HMDB0029062	pos	C9H16N2O5	Organic acids and derivatives
Gamma-Glutamyl-beta-aminopropiononitrile	HMDB0060477	pos	C8H13N3O3	Organic acids and derivatives
4-O-Methyl-a-D-glucosyl-(1->2)-b-D-xylosyl-(1->4)-D-xylose	HMDB0039742	pos	C17H28O15	Organic oxygen compounds
D-Pipecolic acid	HMDB0005960	pos	C6H11NO2	Organic acids and derivatives
Urocanic acid	HMDB0000301	pos	C6H6N2O2	Organoheterocyclic compounds
3-Buten-1-amine	-	pos	C4H9N	-
2-O-a-L-Fucopyranosyl-galactose	HMDB0006590	pos	C12H22O10	Lipids and lipid-like molecules
1-AMINOCYCLOBUTANE CARBOXYLIC ACID	-	pos	C5H9NO2	-
Ectoine	-	pos	C6H10N2O2	-
Gerberinol	HMDB0033304	pos	C21H16O6	Phenylpropanoids and polyketides
L-Carnitine	HMDB0000062	pos	C7H15NO3	Organic nitrogen compounds
Tyrosyl-Hydroxyproline	HMDB0029106	neg	C14H18N2O5	Organic acids and derivatives
Vanillin	HMDB0012308	neg	C8H8O3	Benzenoids
Traumatic Acid	HMDB0000933;LMFA01170002	neg	C12H20O4	Lipids and lipid-like molecules
1,11-Undecanedicarboxylic acid	HMDB0002327	neg	C13H24O4	Lipids and lipid-like molecules
2'-Deoxyuridine	-	neg	C9H12N2O5	-
Hydroxyphenyllactic acid	HMDB0000755	neg	C9H10O4	Phenylpropanoids and polyketides
Armillarinin	HMDB0031674	neg	C24H29ClO7	Lipids and lipid-like molecules
P-Salicylic acid	-	neg	C7H6O3	-
Adipic acid	HMDB0000448;LMFA01170048	neg	C6H10O4	Lipids and lipid-like molecules
Gentisic acid	HMDB0000152	neg	C7H6O4	Benzenoids
Ethyl 5-oxotetrahydro-2-furancarboxylate	HMDB0059815	neg	C7H10O4	Organoheterocyclic compounds
ANHYDROBRAZILIC ACID	-	neg	C12H10O5	-
2-(2,5-dihydroxy-4-methoxyphenyl)acetic acid	HMDB0130476	neg	C9H10O5	Benzenoids
Suberic acid	LMFA01170001;HMDB0000893	neg	C8H14O4	Lipids and lipid-like molecules
Juglone glucoside	HMDB0033563	neg	C16H16O8	Organooxygen compounds
Gamma-Glu-Leu	-	neg	C11H20N2O5	-

Undecanedioic acid	HMDB0000888;LMFA01170007	neg	C11H20O4	Lipids and lipid-like molecules
N-phosphocreatinate(2-)	HMDB0062567	neg	C4H11N3O5P+	-
Riesling acetal	HMDB0037562	neg	C13H22O3	Organoheterocyclic compounds
2-Methylpropyl 2-aminobenzoate	HMDB0035072	neg	C11H15NO2	Benzenoids
2,2'-(3-methylcyclohexane-1,1-diyl)diacetic acid	-	neg	C11H18O4	-
PSOROMIC ACID	-	neg	C18H14O8	-
(8alpha,10beta,11beta)-3-Hydroxy-4,15-dinor-1(5)-xanthen-12,8-olide	HMDB0037244	neg	C13H20O3	Organoheterocyclic compounds
9,10,13-TriHOME	HMDB0004710	neg	C18H34O5	Lipids and lipid-like molecules
L-Citronellol glucoside	HMDB0029850	neg	C16H30O6	Lipids and lipid-like molecules
C75	-	neg	C14H22O4	-
6,15-Diketo,13,14-dihydro-PGF1a	HMDB0001979	neg	C20H32O6	Lipids and lipid-like molecules
Gibberellin A53	LMPR0104170007;HMDB0036895	neg	C20H28O5	Lipids and lipid-like molecules
Tetradecanedioic acid	HMDB0000872;LMFA01170018	neg	C14H26O4	Lipids and lipid-like molecules
Ajmaline	HMDB0015495	neg	C20H26N2O2	Alkaloids and derivatives
Deoxyribose	HMDB0003224	neg	C5H10O4	Organic oxygen compounds
Dulcitol	-	neg	C6H14O6	-
Quinic acid	HMDB0003072	neg	C7H12O6	Organic oxygen compounds
L-Fucose	HMDB0000174	neg	C6H12O5	Organic oxygen compounds
L-Leucyl-L- Alanine	-	neg	C9H18N2O3	-
7-Methyladenine	HMDB0011614	neg	C6H7N5	Organoheterocyclic compounds
2-Keto-6-acetamidocaproate	HMDB0012150	neg	C8H13NO4	Organic acids and derivatives
L-Phenylalanine	HMDB0000159	neg	C9H11NO2	Organic acids and derivatives
2-Ethylglutaric acid	HMDB0059738	neg	C7H12O4	Lipids and lipid-like molecules
Xi-4-Hydroxy-4-methyl-2-cyclohexen-1-one	HMDB0033629	neg	C7H10O2	Organic oxygen compounds
Acetyl-L-tyrosine	-	neg	C11H13NO4	-
5-Methoxysalicylic acid	HMDB0001868	neg	C8H8O4	Benzenoids
7-hydroxy-6-(hydroxymethyl)-2H-chromen-2-one	HMDB0136775	neg	C10H8O4	Phenylpropanoids and polyketides
8-Hydroxy-5,6-octadienoic acid	LMFA01031076;HMDB0031101	neg	C8H12O3	Organic acids and derivatives
1-(4Z,7Z,10Z,13Z,16Z,19Z-docosahexaenoyl)-glycero-3-phosphate	HMDB0062307;LMGP10050019	neg	C25H39O7P	Lipids and lipid-like molecules

4,7-Dihydroxy-2H-1-benzopyran-2-one	HMDB0032951	neg	C9H6O4	Phenylpropanoids and polyketides
2-n-Propyl-4-oxopentanoic acid	HMDB0060683	neg	C8H14O3	Organic acids and derivatives
2-Propylglutaric acid	HMDB0060684	neg	C8H14O4	Lipids and lipid-like molecules
2-Ethylsuberic acid	HMDB0059708	neg	C10H18O4	Lipids and lipid-like molecules
4-Hydroxycoumarin	-	neg	C9H6O3	-
Cis-4-Hydroxycyclohexylacetic acid	HMDB0000451	neg	C8H14O3	Organic oxygen compounds
2-HYDROXY-3,4-DIMETHOXYBENZOIC ACID	HMDB0142084	neg	C9H10O5	Benzenoids
(R)-Byakangelicinn 2'-(3-methylbutanoate)	HMDB0039068	neg	C22H26O8	Phenylpropanoids and polyketides
2,6-DIHYDROXY-4-METHOXYTOLUENE	-	neg	C8H10O3	-
Sinapyl aldehyde	-	neg	C11H12O4	-
Acetyl-DL-Leucine	-	neg	C8H15NO3	-
7-Hydroxy-6-methyl-2H-1-benzopyran-2-one	HMDB0032990	neg	C10H8O3	Phenylpropanoids and polyketides
N-Acetyl-D-phenylalanine	-	neg	C11H13NO3	-
N-formylanthranilic acid	-	neg	C8H7NO3	-
(+)-cis-5,6-Dihydro-5-hydroxy-4-methoxy-6-(2-phenylethyl)-2H-pyran-2-one	HMDB0029575	neg	C14H16O4	Phenylpropanoids and polyketides
4-Hydroxycyclohexylcarboxylic acid	HMDB0001988	neg	C7H12O3	Organic oxygen compounds
3-(4-Methylphenyl)oxiranecarboxylic acid	HMDB0030903	neg	C10H10O3	Benzenoids
(4S,8R)-8,9-Dihydroxy-p-menth-1(6)-en-2-one	HMDB0039052	neg	C10H16O3	Lipids and lipid-like molecules
7-hydroxy-1-oxo-1H-isochromene-3-carboxylic acid	HMDB0128623	neg	C10H6O5	Phenylpropanoids and polyketides
5-(2'-Carboxyethyl)-4,6-Dihydroxypicolinate	HMDB0006794	neg	C9H9NO6	Organoheterocyclic compounds
Pratenol A	HMDB0040630	neg	C14H12O5	Organoheterocyclic compounds
3alpha-Hydroxyoreadone	HMDB0036047	neg	C14H20O4	Organoheterocyclic compounds
Lysyl-Phenylalanine	HMDB0028958	neg	C15H23N3O3	Organic acids and derivatives
Caffeic Acid	HMDB0001964	neg	C9H8O4	Phenylpropanoids and polyketides
METHYLNORLICHEXANTHONE	-	neg	C15H12O5	-
4-Methoxycinnamic acid	HMDB0002040	neg	C10H10O3	Phenylpropanoids and polyketides
Alpha-Phenylcyclohexylglycolic acid	-	neg	C14H18O3	-
2-{[hydroxy(6-hydroxy-2H-1,3-benzodioxol-5-yl)methylidene]amino}acetic acid	HMDB0129401	neg	C10H9NO6	Organic acids and derivatives
2-Propanoylthiophene	HMDB0033159	neg	C7H8OS	Organic oxygen compounds

(E)-10-Hydroxy-8-decenoic acid	HMDB0039533	neg	C10H18O3	Organic acids and derivatives
Monotropein	HMDB0035608;LMPR 0102070012	neg	C16H22O11	Lipids and lipid-like molecules
{[1-(4-methoxyphenyl)-4-methylpent-1-en-3-yl]oxy}sulfonic acid	HMDB0132959	neg	C13H18O5S	Benzenoids
2,4-DINITROPHENOL	-	neg	C6H4N2O5	-
Talaromycin A	HMDB0030143	neg	C12H22O4	Organic oxygen compounds
(1R,2R,3S,1'R)-Nepetalinic acid	HMDB0034971	neg	C10H16O4	Lipids and lipid-like molecules
5-Hydroxy-p-mentha-6,8-dien-2-one	HMDB0037011	neg	C10H14O2	Lipids and lipid-like molecules
2-Carboxy-4-dodecanolide	HMDB0030987	neg	C13H22O4	Organoheterocyclic compounds
2-Hydroxyundecanoate	HMDB0059736	neg	C11H22O3	Lipids and lipid-like molecules
Gamma-CEHC	HMDB0001931	neg	C15H20O4	Organoheterocyclic compounds
13-Oxo-9,11-tridecadienoic acid	LMFA01060202;HMD B0034564	neg	C13H20O3	Lipids and lipid-like molecules
Xi-8,9-Dehydrotheaspirone	HMDB0035181	neg	C13H18O2	Organic oxygen compounds
2-nonenoylglycine	HMDB0094807	neg	C11H19NO3	Organic acids and derivatives
6-(1-carboxy-1-methylethoxy)-3,4,5-trihydroxyoxane-2-carboxylic acid	HMDB0126113	neg	C10H16O9	Organic oxygen compounds
5,7-dihydroxy-2-(4-hydroxyphenyl)-8-(3,4,5-trihydroxyoxan-2-yl)-4H-chromen-4-one	HMDB0127225	neg	C20H18O9	Phenylpropanoids and polyketides
(10betaH,11xi)-11-Hydroxy-13-nor-6-eremophilen-8-one	HMDB0037605	neg	C14H22O2	Lipids and lipid-like molecules
Aflatoxin B2	HMDB0035208	neg	C17H14O6	Phenylpropanoids and polyketides
12,20-Dioxo-leukotriene B4	HMDB0060094	neg	C20H28O5	Lipids and lipid-like molecules
15d PGD2	HMDB0060046	neg	C20H30O4	Lipids and lipid-like molecules
L-Menthyl (R,S)-3-hydroxybutyrate	HMDB0032370	neg	C14H26O3	Lipids and lipid-like molecules
Eremopetasidione	HMDB0040778	neg	C14H20O3	Lipids and lipid-like molecules
3,4,5-trihydroxy-6-{4-[(1E)-3-oxopent-1-en-1-yl]phenoxy}oxane-2-carboxylic acid	HMDB0132988	neg	C17H20O8	Organic oxygen compounds
Panaxytriol	HMDB0031928;LMFA 05000027	neg	C17H26O3	Lipids and lipid-like molecules
Tatridin B	HMDB0036931	neg	C15H20O4	Lipids and lipid-like molecules
3-[[5-Methyl-2-(1-methylethyl)cyclohexyl]oxy]-1,2-propanediol	HMDB0036133	neg	C13H26O3	Lipids and lipid-like molecules
ACRL Toxin II	HMDB0030496	neg	C17H24O5	Lipids and lipid-like molecules
7-Sulfocholic acid	LMST05020039;HMD B0002421	neg	C24H40O8S	Lipids and lipid-like molecules

Annuionone C	HMDB0032689	neg	C13H20O3	Organoheterocyclic compounds
Alpha-CEHC	HMDB0001518	neg	C16H22O4	Organoheterocyclic compounds
Hydroxypelenolide	HMDB0036663	neg	C15H24O3	Lipids and lipid-like molecules
Neocnidilide	HMDB0034450	neg	C12H18O2	Organoheterocyclic compounds
Deacetyldiltiazem	HMDB0002873	neg	C20H24N2O3S	Organoheterocyclic compounds
Ethylene brassylate	HMDB0040459	neg	C15H26O4	Phenylpropanoids and polyketides
Allocholic acid	HMDB0000505;LMST 04010092	neg	C24H40O5	Lipids and lipid-like molecules
8-Deoxy-11,13-dihydroxygrosheimin	HMDB0041144	neg	C15H20O5	Lipids and lipid-like molecules
()12,13-DiHOME	-	neg	C18H34O4	-
Tetranor-12(R)-HETE	-	neg	C16H26O3	-
Zileuton O-glucuronide	HMDB0060914	neg	C17H20N2O8S	Organic oxygen compounds
()13-HpODE	-	neg	C18H32O4	-
Carnosol	HMDB0002121	neg	C20H26O4	Lipids and lipid-like molecules
Neriantogenin	HMDB0030044	neg	C23H32O4	Lipids and lipid-like molecules
Lansiumarin A	HMDB0034837	neg	C21H20O5	Phenylpropanoids and polyketides
LysoPE(15:0/0:0)	HMDB0011502	neg	C20H42NO7P	Lipids and lipid-like molecules
2-Hydroxymyristic Acid	HMDB0002261;LMFA 01050484	neg	C14H28O3	Lipids and lipid-like molecules
Mabiogenin 3-[rhamnosyl-(1->6)-[glucosyl-(1->2)]-glucoside]	HMDB0040942	neg	C48H78O19	Lipids and lipid-like molecules
N-Undecylbenzenesulfonic acid	HMDB0032549	neg	C17H28O3S	Benzenoids
Cincassiol B	HMDB0036855	neg	C20H32O8	Lipids and lipid-like molecules
Armillarivin	HMDB0038917	neg	C23H28O5	-
2-hydroxyhexadecanoic acid	HMDB0031057	neg	C16H32O3	Lipids and lipid-like molecules
LysoPC(18:0)	HMDB0010384	neg	C26H54NO7P	Lipids and lipid-like molecules
5-Fluorodeoxyuridine monophosphate	HMDB0060394	neg	C9H12FN2O8P	Nucleosides, nucleotides, and analogues
2-Dodecylbenzenesulfonic acid	HMDB0031031	neg	C18H30O3S	Benzenoids
PA(18:4(6Z,9Z,12Z,15Z)/14:1(9Z))	LMGP10010426;HMD B0115028	neg	C35H59O8P	Lipids and lipid-like molecules
PG(16:0/0:0)[U]	-	neg	C22H45O9P	-
1-Oleoylglycerophosphoinositol	HMDB0061693	neg	C27H51O12P	Lipids and lipid-like molecules
11,12,14-Trihydroxy-7-methoxy-8,11,13-abietatrien-20,6-olide	HMDB0031932	neg	C21H28O6	Lipids and lipid-like molecules
Cyclopassifloside X	HMDB0036365	neg	C37H62O12	Lipids and lipid-like molecules
PA(14:1(9Z)/20:4(5Z,8Z,11Z,14Z))	LMGP10010129;HMD B0114801	neg	C37H63O8P	Lipids and lipid-like molecules

Dihydro-3-(1-octenyl)-2,5-furandione	HMDB0037816	neg	C12H18O3	Organoheterocyclic compounds
1-(11Z-eicosenoyl)-glycero-3-phosphate	HMDB0062305;LMGP 10050026	neg	C23H45O7P	Lipids and lipid-like molecules
Indacaterol-8-O-glucuronide	HMDB0061152	neg	C30H36N2O9	Organic oxygen compounds
Manglupenone	HMDB0030395	neg	C30H44O2	Lipids and lipid-like molecules
Adenine	HMDB0000034	neg	C5H5N5	Organoheterocyclic compounds
S-Cysteinosuccinic acid	HMDB0029418	neg	C7H11NO6S	Organic acids and derivatives
Nevskin	HMDB0030162	neg	C24H32O5	Phenylpropanoids and polyketides
PS(15:0/16:0)	LMGP03010917;HMD B0112281	neg	C37H72NO10P	Lipids and lipid-like molecules
PE(15:0/16:1(9Z))	HMDB0008891;LMGP 02011235	neg	C36H70NO8P	Lipids and lipid-like molecules
20-Trihydroxy-leukotriene-B4	LMFA03020066;HMD B0012643	neg	C20H32O7	Lipids and lipid-like molecules
(R)-6'-O-(4-Geranyloxy-2-hydroxycinnamoyl)-marmin	HMDB0039061	neg	C38H46O8	Lipids and lipid-like molecules
1-Palmitoylglycerophosphoinositol	HMDB0061695	neg	C25H49O12P	Lipids and lipid-like molecules
Theasapogenol A	HMDB0034519	neg	C30H50O6	Lipids and lipid-like molecules
13,14-Dihydro-15-keto-PGE2	HMDB0002776;LMFA 03010031	neg	C20H32O5	Lipids and lipid-like molecules
Alpha-Ionol O-[arabinosyl-(1->6)-glucoside]	HMDB0038732	neg	C24H40O10	-
LysoPC(16:0)	HMDB0010382	neg	C24H50NO7P	Lipids and lipid-like molecules
Ethisterone	HMDB0060580	neg	C21H28O2	Lipids and lipid-like molecules
Methyl cis-p-coumarate 3-(3,7-dimethyl-2,6-octadienyl)	HMDB0041152	neg	C20H26O3	Phenylpropanoids and polyketides
LysoPE(16:1(9Z)/0:0)	HMDB0011504	neg	C21H42NO7P	Lipids and lipid-like molecules
Armillasin	HMDB0038742	neg	C22H28O5	-
Mannitol 1-phosphate	HMDB0001530	neg	C6H15O9P	Organic oxygen compounds
TOFA	-	neg	C19H32O4	-
(3S,7E,9S)-9-Hydroxy-4,7-megastigmadien-3-one 9-glucoside	HMDB0036822	neg	C19H30O7	Lipids and lipid-like molecules
1-(2,6,6-Trimethyl-2-cyclohexen-1-yl)-1,6-heptadien-3-one	HMDB0029704	neg	C16H24O	Lipids and lipid-like molecules
()9-HODE	-	neg	C18H32O3	-
Formebolone	HMDB0004631	neg	C21H28O4	Lipids and lipid-like molecules
()-(E)-13-Hydroxy-10-oxo-11-octadecenoic acid	HMDB0040900	neg	C18H32O4	Lipids and lipid-like molecules
LysoPE(14:1(9Z)/0:0)	HMDB0011501	neg	C19H38NO7P	Lipids and lipid-like molecules

4-Hydroxyretinoic acid	HMDB0006254	neg	C20H28O3	Lipids and lipid-like molecules
Salsoline-1-carboxylate	HMDB0013067	neg	C12H15NO4	Organoheterocyclic compounds
5-Tetradecenoic acid	HMDB0000499	neg	C14H26O2	Lipids and lipid-like molecules
Glandulone C	HMDB0039859	neg	C15H20O3	Lipids and lipid-like molecules
12-hydroxyheptadecanoic acid	HMDB0061663	neg	C17H34O3	Lipids and lipid-like molecules
Acetylvalerenolic acid	HMDB0035687	neg	C17H24O4	Lipids and lipid-like molecules
Dehydrovomifoliol	HMDB0036819;LMPR 0103050009	neg	C13H18O3	Lipids and lipid-like molecules
MG(0:0/14:1(9Z)/0:0)	HMDB0011531	neg	C17H32O4	Lipids and lipid-like molecules
Aflatoxin GM1	HMDB0030477	neg	C17H12O8	Phenylpropanoids and polyketides
Melleolide D	HMDB0037042	neg	C24H31ClO8	Lipids and lipid-like molecules
Nonanal propyleneglycol acetal	HMDB0032440	neg	C12H24O2	Organoheterocyclic compounds
2,4,5,7alpha-Tetrahydro-1,4,4,7a-tetramethyl-1H-inden-2-ol	HMDB0036684	neg	C13H20O	Organic oxygen compounds
TUBAIC ACID	-	neg	C12H12O4	-
Valerenic acid	HMDB0030016;LMPR 0103460001	neg	C15H22O2	Lipids and lipid-like molecules
Octadecanedioic acid	LMFA01170029;HMD B0000782	neg	C18H34O4	Lipids and lipid-like molecules
2,3-dinor Prostaglandin E1	-	neg	C18H30O5	-
Sterebin G	HMDB0035380	neg	C20H34O5	Lipids and lipid-like molecules
Costunolide	HMDB0036688	neg	C15H20O2	Lipids and lipid-like molecules
Tetranor-PGAM	-	neg	C16H22O6	-
Dihydro-3-coumaric acid	-	neg	C9H10O3	-
(E)-10-Oxo-8-decenoic acid	LMFA01030949;HMD B0040883	neg	C10H16O3	Lipids and lipid-like molecules
3,4-Dimethyl-5-pentyl-2-furanpentanoic acid	HMDB0112085	neg	C16H26O3	Lipids and lipid-like molecules
3''-O-Caffeoylcosmosiin	HMDB0037345	neg	C30H26O13	Phenylpropanoids and polyketides
13-Nor-6-eremophilene-8,11-dione	HMDB0037606	neg	C14H20O2	Lipids and lipid-like molecules
7,8-Dehydroastaxanthianthin	HMDB0036872	neg	C40H50O4	Lipids and lipid-like molecules
3-phenyl-3,4-dihydro-2H-1-benzopyran-7-ol	HMDB0141210	neg	C15H14O2	Phenylpropanoids and polyketides
11,12,13-Trinor-1,3,5-bisabolatrien-10-oic acid	HMDB0039643	neg	C12H16O2	Lipids and lipid-like molecules
2'-Oxoaloesol 7-glucoside	HMDB0035734	neg	C19H22O9	Organic oxygen compounds
Pantoyllactone glucoside	HMDB0041269	neg	C12H20O8	Lipids and lipid-like molecules
Xi-7-Hydroxyhexadecanedioic acid	HMDB0037830	neg	C16H30O5	Lipids and lipid-like molecules

3,4-DHPEA-EA	HMDB0029304	neg	C19H22O8	Lipids and lipid-like molecules
Histidinyl-Proline	HMDB0028893	neg	C11H16N4O3	Organic acids and derivatives
3,14-Dihydroxy-11,13-dihydrocostunolide	HMDB0035647	neg	C15H22O4	Lipids and lipid-like molecules
6-Hydroxypentadecanedioic acid	HMDB0031885	neg	C15H28O5	Lipids and lipid-like molecules
5,6-Dihydro-4-methoxy-6-[2-(4-methoxyphenyl)ethyl]-2H-pyran-2-one	HMDB0030549	neg	C15H18O4	Phenylpropanoids and polyketides
3,4-Dimethyl-5-pentyl-2-furanpropanoic acid	HMDB0112083	neg	C14H22O3	Lipids and lipid-like molecules
Comosin	HMDB0033254	neg	C19H18O8	Phenylpropanoids and polyketides
3-(2H-1,3-benzodioxol-5-yl)-3-oxopropanoic acid	HMDB0129376	neg	C10H8O5	Organoheterocyclic compounds
Chlorogenoquinone	HMDB0029383	neg	C16H16O9	Organic oxygen compounds
Marindinin	HMDB0029504	neg	C14H16O3	Phenylpropanoids and polyketides
Quadrone	-	neg	C15H20O3	-
BENTAZON	-	neg	C10H12N2O3S	-
Cucurbic acid	HMDB0029388	neg	C12H20O3	Lipids and lipid-like molecules
3-Oxododecanoic acid	HMDB0010727	neg	C12H22O3	Organic acids and derivatives
(S)-5'-Deoxy-5'-(methylsulfinyl)adenosine	HMDB0033662	neg	C11H15N5O4S	Nucleosides, nucleotides, and analogues
5-Hydroxyflavone	HMDB0040556	neg	C21H20O8	Phenylpropanoids and polyketides
L-Thyronine	HMDB0000667	neg	C15H15NO4	Organic acids and derivatives
1,8-Dihydroxy-3-hydroxymethylanthraquinone 8-O-b-D-glucoside	HMDB0029679	neg	C21H20O10	Benzenoids
1-Methyladenosine	HMDB0003331	neg	C11H15N5O4	Nucleosides, nucleotides, and analogues
Grevilline A	HMDB0029535	neg	C18H12O6	Organoheterocyclic compounds
Eremopetasinorol	HMDB0029668	neg	C13H20O2	Organooxygen compounds
Polyethylene, oxidized	HMDB0032472	neg	C12H20O5	Lipids and lipid-like molecules
8-Hydroxy-2-methoxy-6-methyl-1,4-naphthoquinone	HMDB0030769	neg	C12H10O4	Benzenoids
Phenethylamine glucuronide	HMDB0010323	neg	C14H19NO6	Organic oxygen compounds
(S)-9-Hydroxy-10-undecenoic acid	HMDB0032662;LMFA01050436	neg	C11H20O3	Organic acids and derivatives
Withaperuvin E	HMDB0030127	neg	C28H36O8	Lipids and lipid-like molecules
3-Oxooctanoic acid	HMDB0010721	neg	C8H14O3	Organic acids and derivatives
3,5-dichlorosalicylic acid	-	neg	C7H4Cl2O3	-
Cystathionine ketimine	HMDB0002015	neg	C7H9NO4S	Organic acids and derivatives
5-Deoxyribose-1-phosphate	HMDB0060393	neg	C5H11O7P	Organic oxygen compounds
8-Hydroxyguanosine	HMDB0002044	neg	C10H13N5O6	Nucleosides, nucleotides, and analogues

Azelaic acid	HMDB0000784;LMFA01170054	neg	C9H16O4	Lipids and lipid-like molecules
CRESOPYRINE	-	neg	C10H10O4	-
(Z)-5-[(5-Methyl-2-thienyl)methylene]-2(5H)-furanone	HMDB0033845	neg	C10H8O2S	Organoheterocyclic compounds
3,5-DINITROCATECHOL (OR-486)	-	neg	C6H4N2O6	-
2,4-Dihydroxy-7-methoxy-2H-1,4-benzoxazin-3(4H)-one	HMDB0034864	neg	C9H9NO5	Organoheterocyclic compounds
4-Nitrophenol	HMDB0062627;HMDB0001232	neg	C6H5NO3	Benzenoids
Ureidoisobutyric acid	HMDB0002031	neg	C5H10N2O3	Organic acids and derivatives
(3R,5Z)-5-Octene-1,3-diol	LMFA05000556;HMDB0030368	neg	C8H16O2	Lipids and lipid-like molecules
Ferulic acid	-	neg	C10H10O4	-
Amyl salicylate	HMDB0038604	neg	C12H16O3	Benzenoids
6-Methoxy-2(3H)-benzoxazolone	HMDB0036582	neg	C8H7NO3	Organoheterocyclic compounds
6-[(E)-2-(3,4-dihydroxyphenyl)ethenyl]-4-methoxy-2H-pyran-2-one	HMDB0131199	neg	C14H12O5	Phenylpropanoids and polyketides
6-beta-hydroxymedroxyprogesterone	HMDB0061063	neg	C22H32O4	Lipids and lipid-like molecules
(1beta,2beta,5beta)-p-Menth-3-ene-1,2,5-triol	HMDB0038978	neg	C10H18O3	Lipids and lipid-like molecules
9-Oxo-nonanoic acid	HMDB0094711;LMFA01060160	neg	C9H16O3	Lipids and lipid-like molecules
DL-Benzylsuccinic acid	-	neg	C11H12O4	-
6,8-dihydroxy-7-methoxy-2H-chromen-2-one	HMDB0130546	neg	C10H8O5	Phenylpropanoids and polyketides
4-Hydroxy-8-methoxy-2H-furo[2,3-h]-1-benzopyran-2-one	HMDB0032659	neg	C12H8O5	Phenylpropanoids and polyketides
8H-1,3-Dioxolo[4,5-h][1]benzopyran-8-one	HMDB0039045	neg	C10H6O4	Phenylpropanoids and polyketides
4-Nitrocatechol	HMDB0002916	neg	C6H5NO4	Benzenoids
Phenyllactic acid	HMDB0000779	neg	C9H10O3	Phenylpropanoids and polyketides
3,4,5-trihydroxy-6-(3-methoxyphenoxy)oxane-2-carboxylic acid	HMDB0135642	neg	C13H16O8	Organic oxygen compounds
Botryodiplodin	HMDB0036594	neg	C7H12O3	Organoheterocyclic compounds
Urolithin B	HMDB0013696	neg	C13H8O3	Phenylpropanoids and polyketides
5,7-Dihydroxy-3-(3-hydroxy-4-methoxybenzyl)-4-chromanone	HMDB0037477	neg	C17H16O6	Phenylpropanoids and polyketides
Indole-3-carboxylic acid	HMDB0003320	neg	C9H7NO2	Organoheterocyclic compounds
Xanthurenic acid	HMDB0000881	neg	C10H7NO4	Organoheterocyclic compounds

2beta,9xi-Dihydroxy-8-oxo-1(10),4,11(13)-germacratrien-12,6alpha-olide	HMDB0036662	neg	C15H18O5	Lipids and lipid-like molecules
Benzoquinoneacetic acid	HMDB0002334	neg	C8H6O4	Organic oxygen compounds
1-oxo-1H-isochromene-3-carboxylic acid	HMDB0128616	neg	C10H6O4	Phenylpropanoids and polyketides
Scopoletin	HMDB0034344	neg	C10H8O4	Phenylpropanoids and polyketides
DIPROTIN A	-	neg	C17H31N3O4	-
Methyl (3b,11x)-3-Hydroxy-8-oxo-6-eremophilen-12-oate	HMDB0041229	neg	C16H24O4	Lipids and lipid-like molecules
Alpha-Carboxy-delta-nonalactone	HMDB0030990	neg	C10H16O4	Organoheterocyclic compounds
1,4-Benzodioxin-2(3H)-one	HMDB0040528	neg	C8H6O3	Organoheterocyclic compounds
Gamma-Glutamyltryptophan	HMDB0029160	neg	C16H19N3O5	Organic acids and derivatives
3-Methyladipic acid	HMDB0000555	neg	C7H12O4	Lipids and lipid-like molecules
Uralenneoside	HMDB0041272	neg	C12H14O8	Benzenoids
7-Hydroxy-2-methyl-4-oxo-4H-1-benzopyran-5-carboxylic acid	HMDB0036346	neg	C11H8O5	Organoheterocyclic compounds
2-Isopropylmalic acid	HMDB0000402	neg	C7H12O5	Lipids and lipid-like molecules
3-hydroxy-3-(3-hydroxyphenyl)propanoic acid-O-sulphate	HMDB0059967	neg	C9H10O7S	Organic acids and derivatives
6-O-Methylarmillaridin	HMDB0035690	neg	C25H31ClO6	Lipids and lipid-like molecules
Acetyl-DL-Valine	-	neg	C7H13NO3	-
Imidaprilat	HMDB0041908	neg	C18H23N3O6	Organic acids and derivatives
(-)-trans-3,4-Dihydro-4,8-dihydroxy-3-methyl-1H-2-benzopyran-1-one	HMDB0030765	neg	C10H10O4	Organoheterocyclic compounds
Propyl propane thiosulfonate	HMDB0032496	neg	C11H16O5	Organooxygen compounds
5-[(2,4,5-trihydroxyphenyl)methyl]oxolan-2-one	HMDB0128183	neg	C11H12O5	Benzenoids
Phyllanthusol B	HMDB0035904	neg	C35H49NO18	Organic oxygen compounds
Neoacrimarine K	HMDB0033192	neg	C31H29NO9	Phenylpropanoids and polyketides
2-(sulfooxy)acetic acid	HMDB0124940	neg	C2H4O6S	Organic acids and derivatives
4-Pyridoxic acid	HMDB0000017	neg	C8H9NO4	Organoheterocyclic compounds
Deoxyguanosine	HMDB0000085	neg	C10H13N5O4	Nucleosides, nucleotides, and analogues
Guanosine	HMDB0000133	neg	C10H13N5O5	Nucleosides, nucleotides, and analogues
Gentiotriose	HMDB0029910	neg	C18H32O16	Organic oxygen compounds
Hydroxyminaline	HMDB0034368	neg	C5H5NO3	Organoheterocyclic compounds
(3,4,5,6-tetrahydroxyoxan-2-yl)methyl acetate	HMDB0125202	neg	C8H14O7	-
2-C-Methyl-1,4-erythrono-D-lactone	HMDB0029884	neg	C5H8O4	Organoheterocyclic compounds
Isocitrate	-	neg	C6H8O7	-

N-Acetyl-DL-serine	-	neg	C5H9NO4	-
O2'-4a-cyclic-tetrahydrobiopterin	HMDB0013031	neg	C9H13N5O3	Organoheterocyclic compounds
Panose	HMDB0029937	neg	C18H32O16	Organic oxygen compounds

Note: pos and neg are positive ion and negative ion respectively.

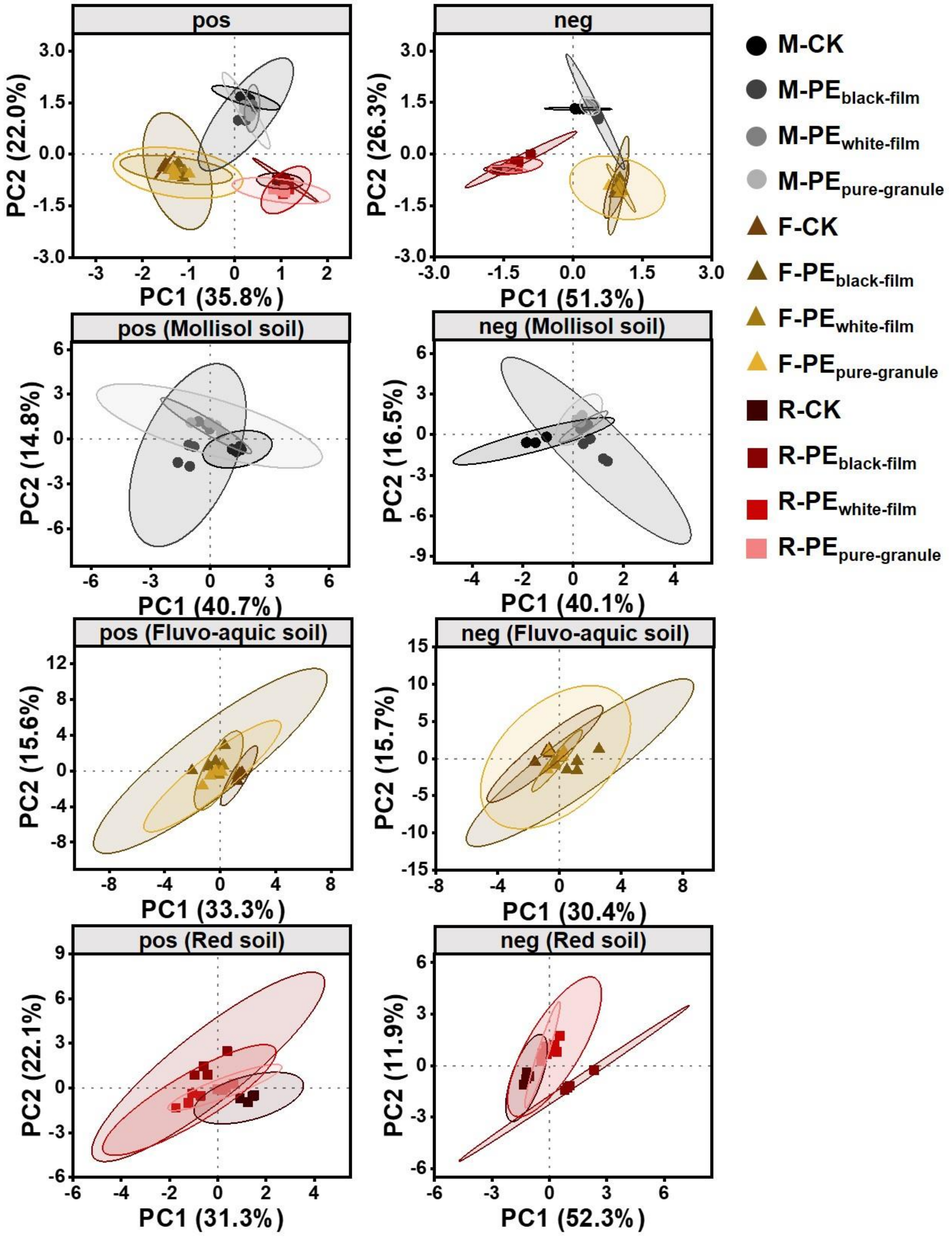

Figure S6. The principal component analysis of water extracted soil metabolites. Pos and neg are positive ion and negative ion respectively. CK: Pure soil solution; PE$_{black-film}$: black polyethylene film microplastics; PE$_{white-film}$: white polyethylene film microplastics; PE$_{pure-granule}$: pure polyethylene microplastic granules.

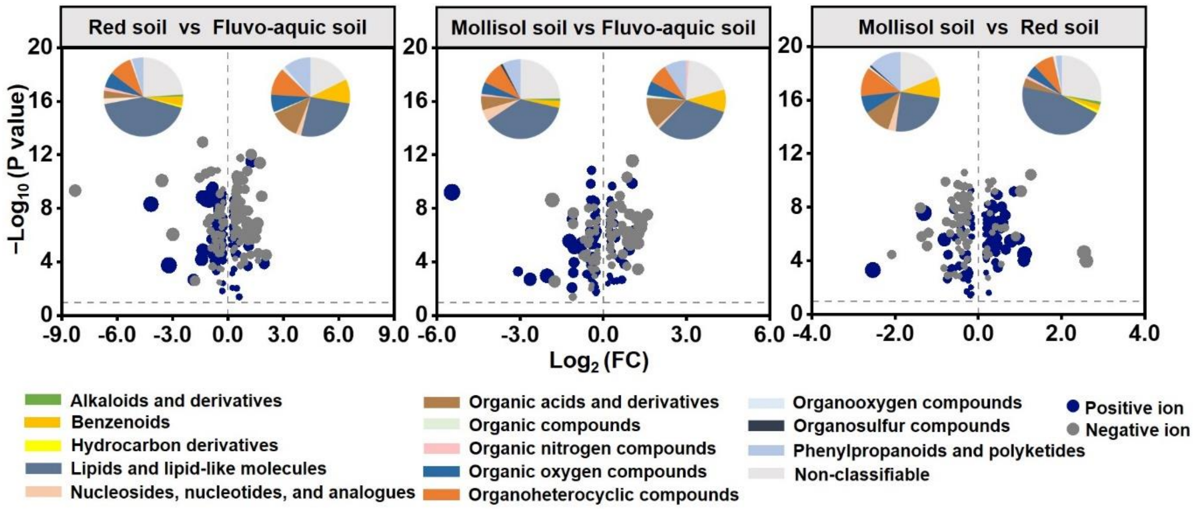

Figure S7. A volcano-plot of water extracted soil metabolites in different soils. For example, within the chart of Red soil vs Fluvo-aquic soil, FC = Fluvo-aquic soil / Red soil. The positive Log_2(FC) values mean those metabolites were abundant in Fluvo-aquic soil, while the negative Log_2(FC) values mean those metabolites were abundant in Red soil. The $-Log_{10}$(p value) higher than 1.30 indicates that the significance of all data was in level of $p \leq 0.05$.

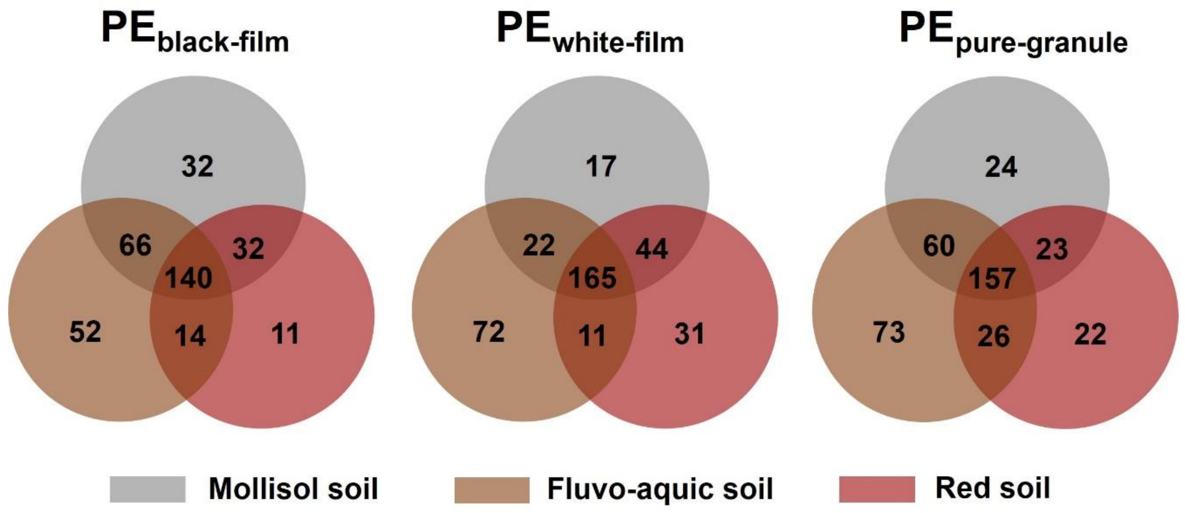

Figure S8. Veen plot of decreased ($p \leq 0.05$) water extracted soil metabolites of different soils (mollisol soil, fluvo-aquic soil, red soil) after interaction with black polyethylene film microplastics ($PE_{black\text{-}film}$), white polyethylene film microplastics ($PE_{white\text{-}film}$) and pure polyethylene microplastic granules ($PE_{pure\text{-}granule}$).

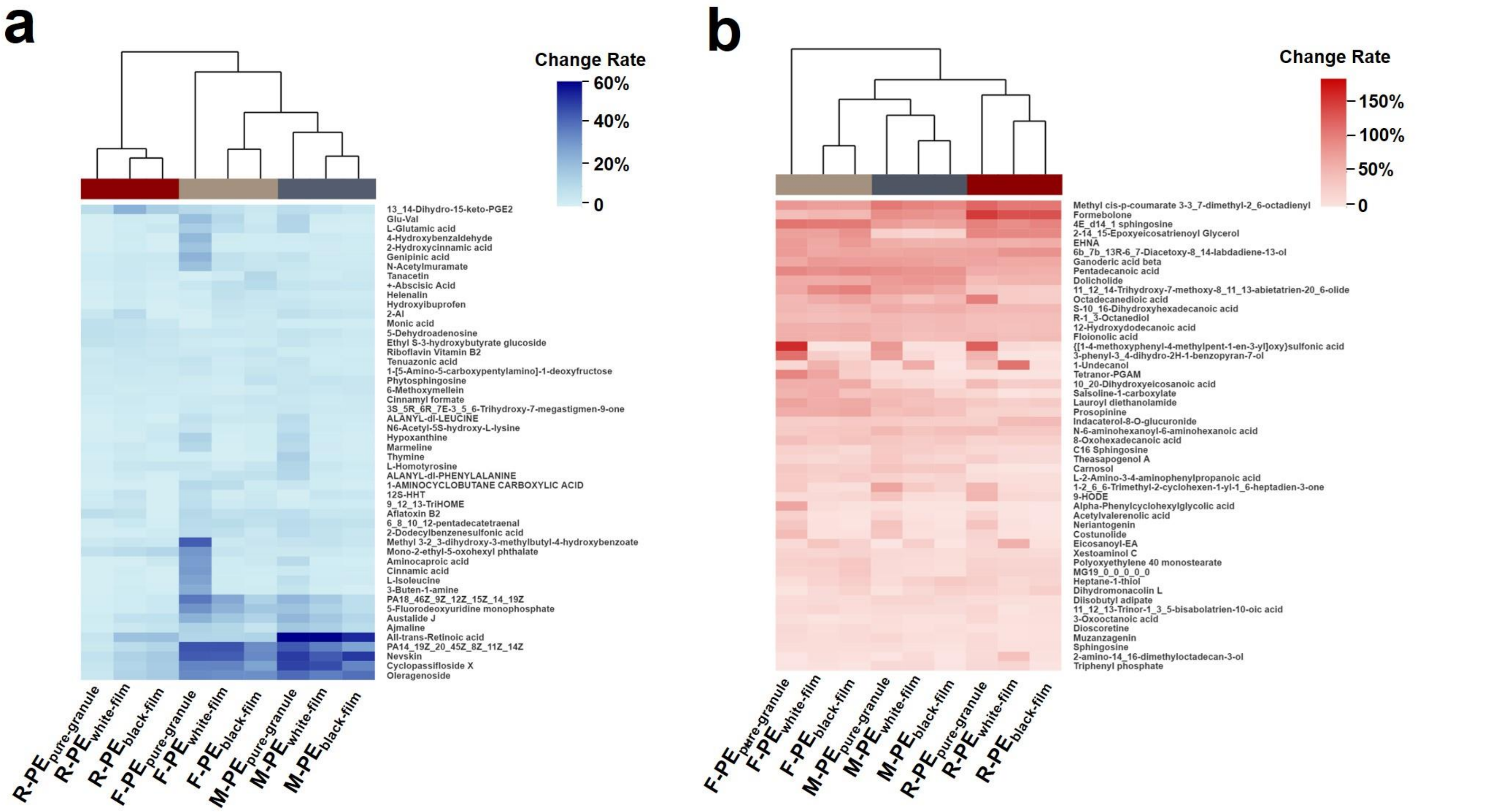

Figure S9. The relative change in decreased (a) and increased (b) water extracted soil metabolites of different soil solutions (mollisol soil (M), fluvo-aquic soil (F), red soil (R)) after interaction with black polyethylene film microplastics ($PE_{black\text{-}film}$), white polyethylene film microplastics ($PE_{white\text{-}film}$) and pure polyethylene microplastic granules ($PE_{pure\text{-}granule}$).

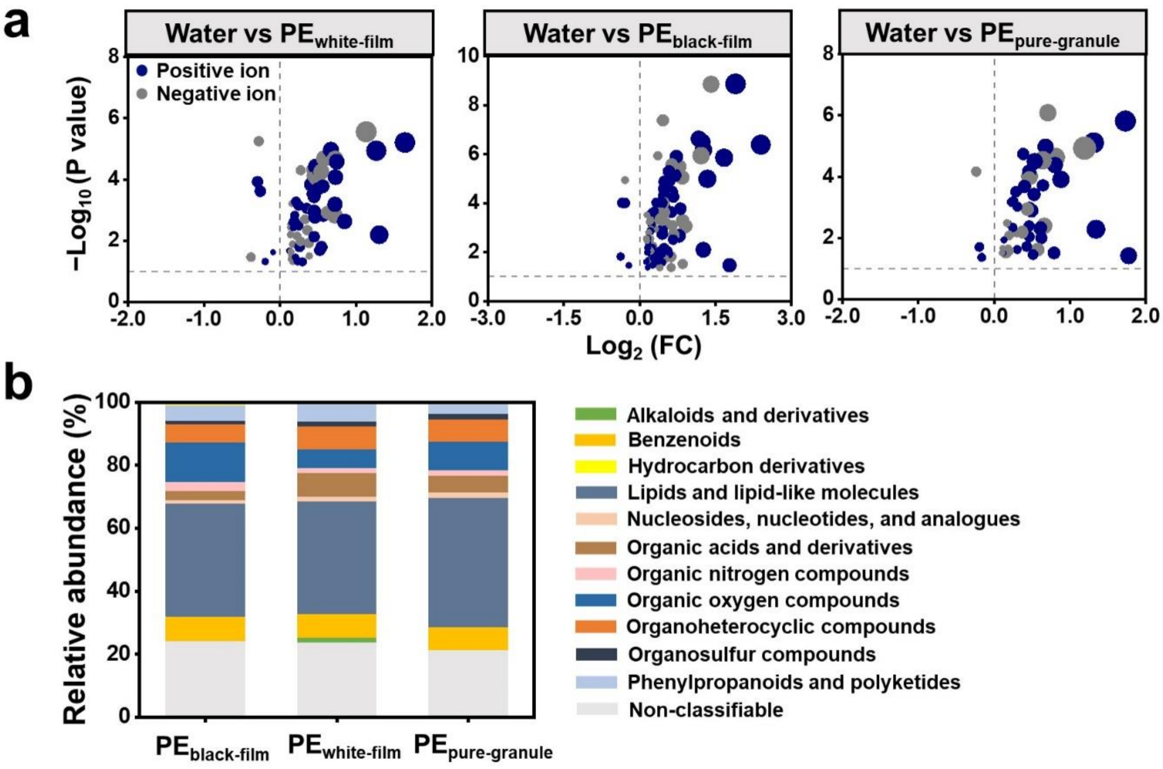

Figure S10. The volcano-plot (a) and compounds composition (b) of compounds released by black polyethylene film microplastics ($PE_{black\text{-}film}$), white polyethylene film microplastics ($PE_{white\text{-}film}$) and pure polyethylene microplastic granules ($PE_{pure\text{-}granule}$) in water. The FC value is quantified as the ratio of the concentrations of each metabolite groups in the two systems. For example, within the chart of Water vs $PE_{black\text{-}film}$, FC = $PE_{black\text{-}film}$ / Water. The positive Log_2(FC) values mean those metabolites were abundant in $PE_{black\text{-}film}$, while the negative Log_2(FC) values mean those metabolites were abundant in Water. The $-Log_{10}$(p value) higher than 1.30 indicates that the significance of all data was in level of $p \leq 0.05$.

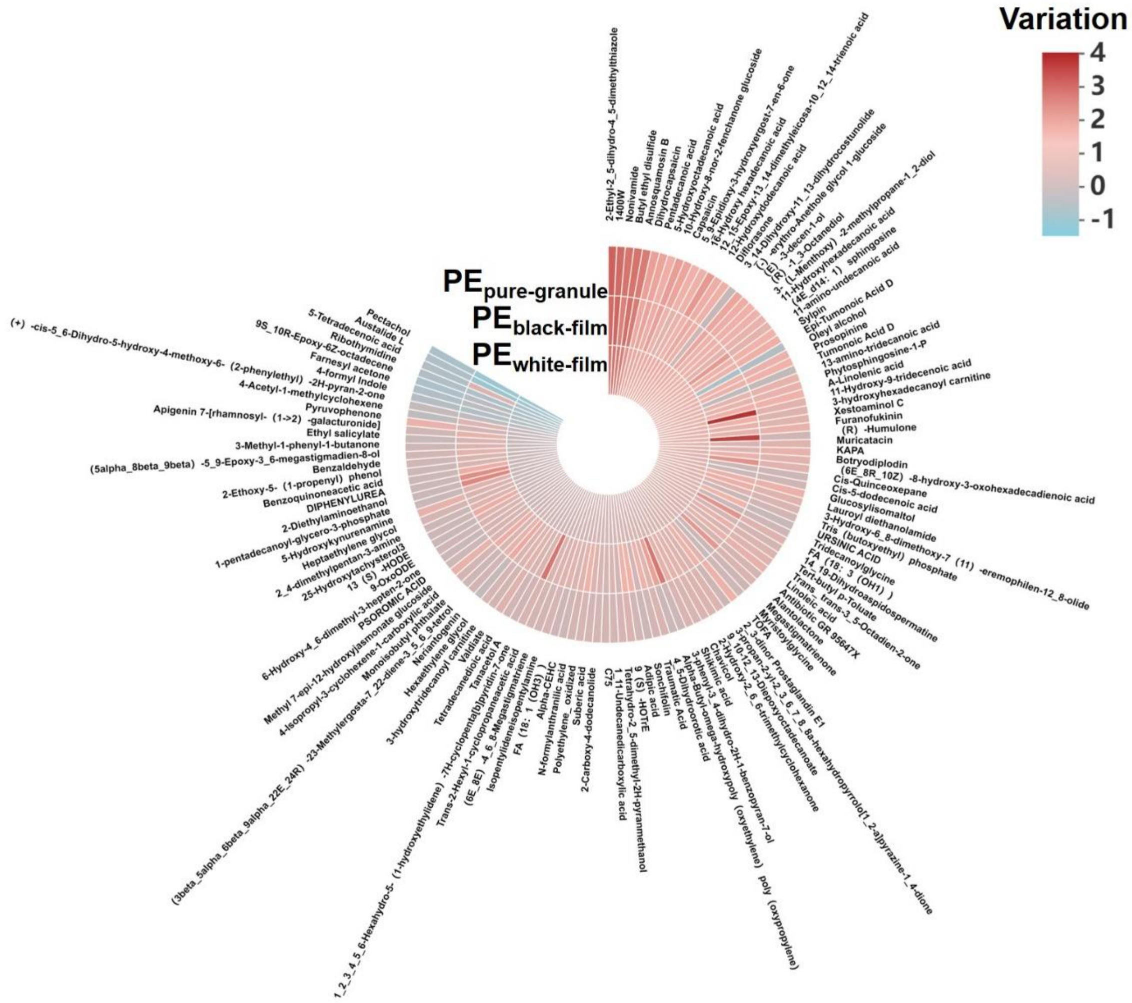

Figure S11. The variation of compounds released from black polyethylene film microplastics ($PE_{black\text{-}film}$), white polyethylene film microplastics ($PE_{white\text{-}film}$) and pure polyethylene microplastic granules ($PE_{pure\text{-}granule}$) in water.

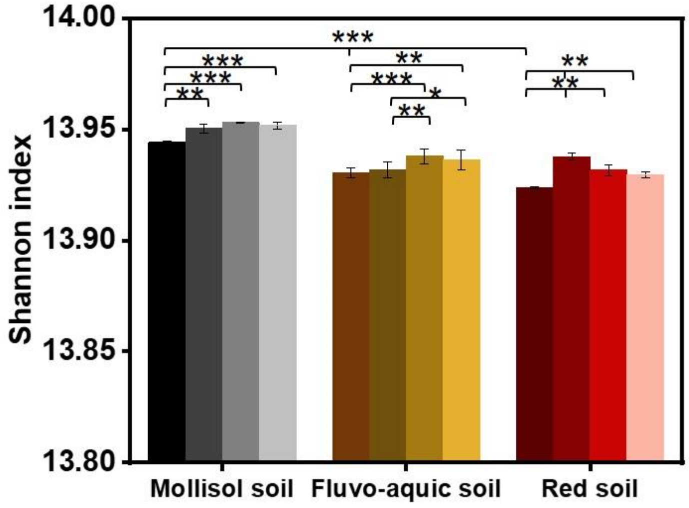

Figure S12. The Shannon index of water extracted soil metabolites in different soils before and after microplastic exist. The color depth of each soil system was in order of pure soil solution control group, black polyethylene film microplastics, white polyethylene film microplastics and pure polyethylene microplastic granules treatments. (***$p \leq 0.001$, **$p \leq 0.01$, *$p \leq 0.05$).

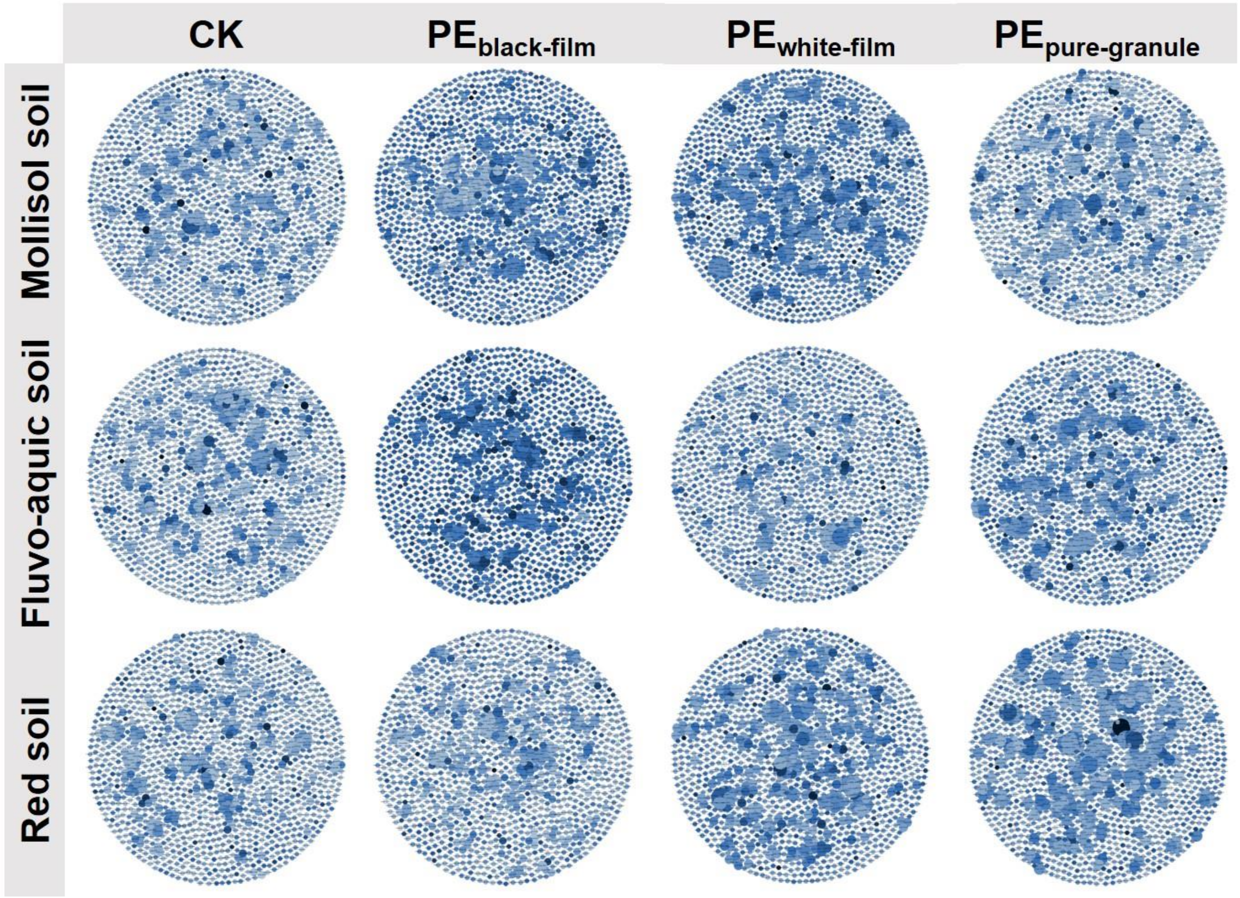

Figure S13. The co-occurrence network of water extracted soil metabolites with or without microplastics. The node size indicates the degree of connectivity and the color shade represents the abundance of metabolites. CK: solution without microplastics; $PE_{black-film}$: black polyethylene film microplastics; $PE_{white-film}$: white polyethylene film microplastics; $PE_{pure-granule}$: pure polyethylene microplastic granules.

Table S4. The co-occurrence network parameters of water extracted soil metabolites with or without microplastics. CK: solution without microplastics; $PE_{black\text{-}film}$: black polyethylene film microplastics, $PE_{white\text{-}film}$: white polyethylene film microplastics, $PE_{pure\text{-}granule}$: pure polyethylene microplastic granules.

		Node	Eage	Average degree	Modularization	Average clustering coefficient
Mollisol soil	CK	1277	794	0.622	0.997	0.056
	$PE_{black\text{-}film}$	1270	1061	0.853	0.984	0.071
	$PE_{white\text{-}film}$	1298	789	0.608	0.998	0.059
	$PE_{pure\text{-}granule}$	1223	795	0.650	0.997	0.064
Fluvo-aquic soil	CK	1254	795	0.634	0.997	0.061
	$PE_{black\text{-}film}$	1217	817	0.671	0.995	0.062
	$PE_{white\text{-}film}$	1263	790	0.625	0.996	0.049
	$PE_{pure\text{-}granule}$	1241	795	0.641	0.997	0.064
Red soil	CK	1317	789	0.599	0.997	0.046
	$PE_{black\text{-}film}$	1228	791	0.644	0.996	0.054
	$PE_{white\text{-}film}$	1252	786	0.628	0.997	0.064
	$PE_{pure\text{-}granule}$	1324	790	0.597	0.998	0.038

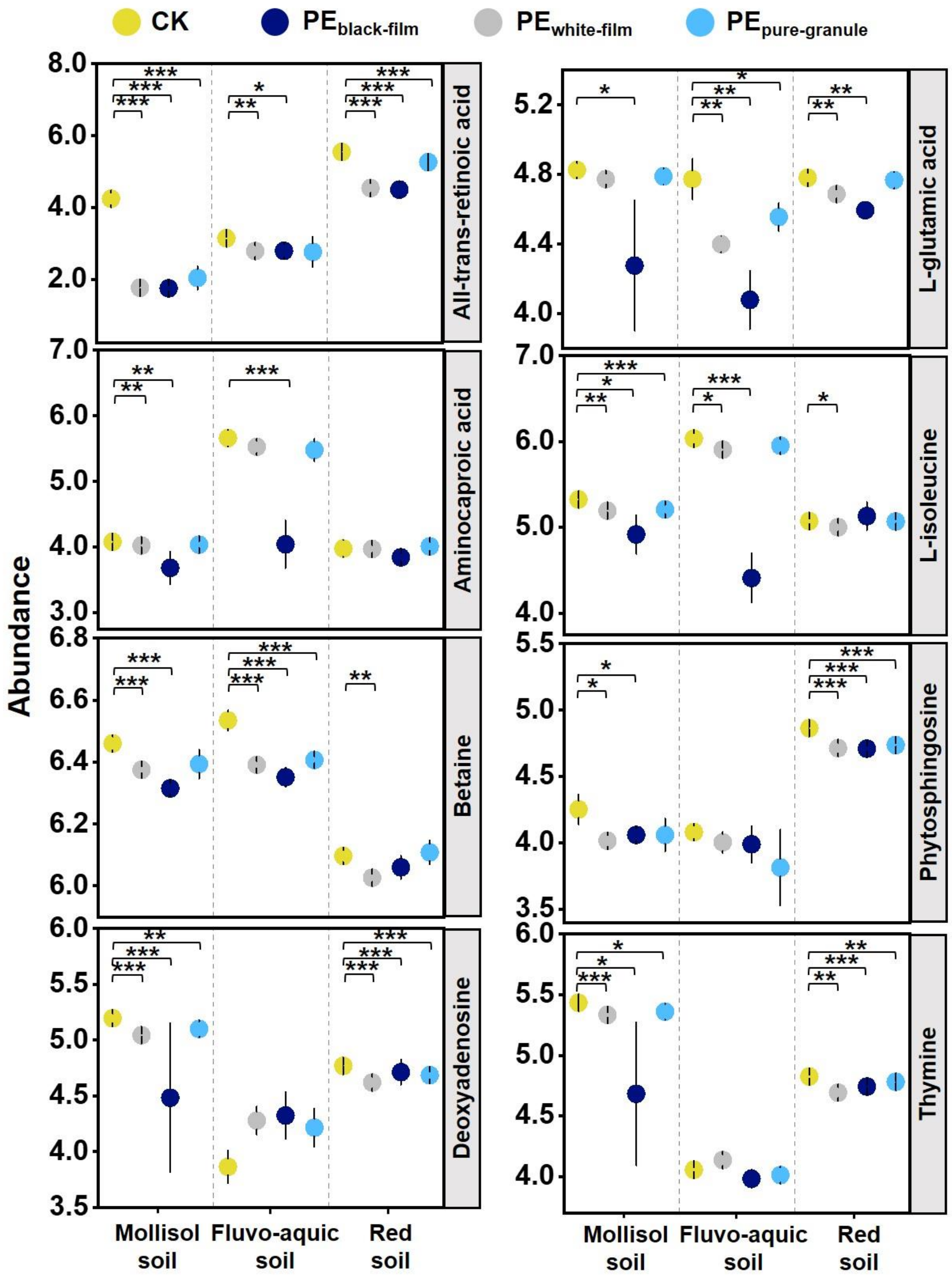

Figure S14. The abundance of all-trans-retinoic acid, thymine, aminocaproic acid, betaine, l-isoleucine, l-glutamic acid, phytosphingosine and deoxyadenosin in water extracted soil metabolites with or without microplastics. CK: solution without microplastics; $PE_{black\text{-}film}$: black polyethylene film microplastics; $PE_{white\text{-}film}$: white polyethylene film microplastics; $PE_{pure\text{-}granule}$: pure polyethylene microplastic granules (***$p \leq 0.001$, **$p \leq 0.01$, *$p \leq 0.05$).

Table S5. Kinetics parameters and partition ratios for all-trans-retinoic acid, aminocaproic acid, betaine, deoxyadenosin and thymine sorption on microplastics. PE$_{black-film}$: black polyethylene film microplastics, PE$_{white-film}$: white polyethylene film microplastics, PE$_{pure-granule}$: pure polyethylene microplastic granules.

		All-trans-retinoic acid			Aminocaproic acid			Betaine			Deoxyadenosin			Thymine		
		PE$_{black-film}$	PE$_{white-film}$	PE$_{pure-granule}$	PE$_{black-film}$	PE$_{white-film}$	PE$_{pure-granule}$	PE$_{black-film}$	PE$_{white-film}$	PE$_{pure-granule}$	PE$_{black-film}$	PE$_{white-film}$	PE$_{pure-granule}$	PE$_{black-film}$	PE$_{white-film}$	PE$_{pure-granule}$
Pesudo-first-order	k_1	2.83cd	3.83d	2.90cd	0.72a	2.02bc	-	0.47a	0.86ab	2.05bc	-	-	0.64a	0.06a	-	0.38a
	Q_e	7.04g	7.81h	7.91i	0.23a	0.60d	-	0.48c	0.37b	1.20f	-	-	0.40bc	0.40bc	-	0.25a
	R^2	0.99	0.99	0.99	0.52	0.75	-	0.73	0.97	0.96	-	-	0.91	0.98	-	0.98
Partition ratio	K_p	7.33a	42.11b	87.89c	0.03a	0.08a	-	0.06a	0.05a	0.18a	-	-	0.05a	0.05a	-	0.03a

Note: Q_e is the sorption amount at sorption equilibrium respectively (mg kg^{-1}); k_1 is pseudo-first-order rate constants, and the units is (min^{-1}); K_p is equilibrium partition ratio between all-trans-retinoic acid, aminocaproic acid, betaine, deoxyadenosin and thymine concentration in microplastics and water. The lower case letters in each column represent the significance of the difference at $p \leq 0.05$.

Table S6. The pH of the different solution. Background solution: LC-MS water with 0.02% NaN_3 solution; $PE_{black\text{-}film}$: black polyethylene film microplastics, $PE_{white\text{-}film}$: white polyethylene film microplastics, $PE_{pure\text{-}granule}$: pure polyethylene microplastic granules, BSA: Bovine serum albumin.

Solution	pH
Background solution	6.92
Background solution + $PE_{white\text{-}film}$	7.14
Background solution + $PE_{black\text{-}film}$	7.41
Background solution + $PE_{pure\text{-}granule}$	7.19
Background solution + BSA coated $PE_{white\text{-}film}$	7.04
Background solution + BSA coated $PE_{black\text{-}film}$	7.28
Background solution + BSA coated $PE_{pure\text{-}granule}$	7.17

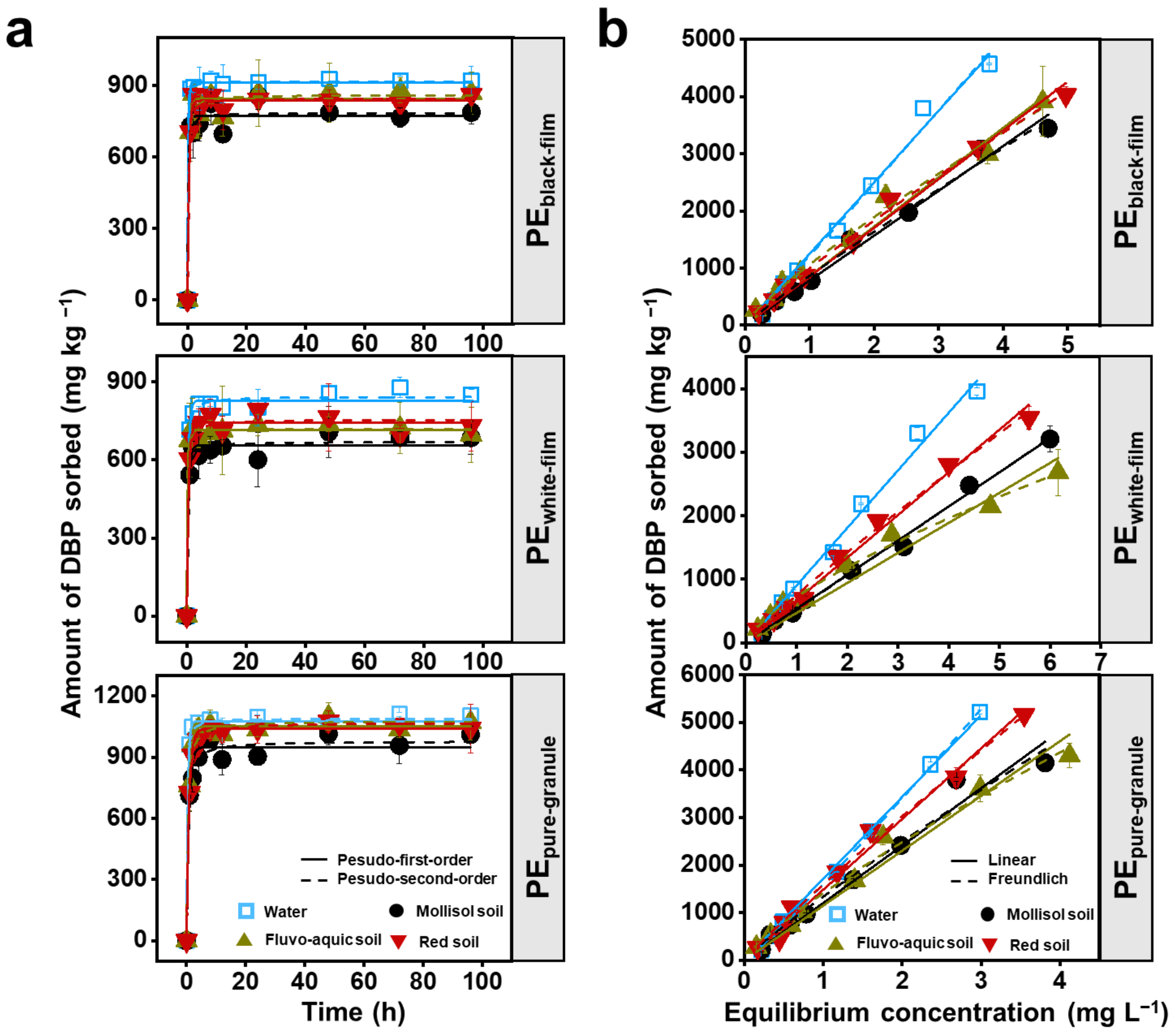

Figure S15. Sorption kinetic (a) and isotherms (b) of dibutyl phthalate (DBP) on black polyethylene film microplastics ($PE_{black\text{-}film}$), white polyethylene film microplastics ($PE_{white\text{-}film}$) and pure polyethylene microplastic granules ($PE_{pure\text{-}granule}$) with water or water extractable soil metabolites from different soils as backrogund solutions, respectively.

Table S7. Kinetics, isotherms parameters and partition ratios of dibutyl phthalate (DBP) onto microplastics. $PE_{black\text{-}film}$: black polyethylene film microplastics, $PE_{white\text{-}film}$: white polyethylene film microplastics, $PE_{pure\text{-}granule}$: pure polyethylene microplastic granules.

		Pseudo-first-order			Pseudo-second-order			Linear		Freundlich			Partition ratio
		k_1	Q_e	R^2	k_2	Q_e	R^2	K_d	R^2	K_F	$1/n$	R^2	K_p
$PE_{black\text{-}film}$	Water	3.52e	911.41f	0.99	0.024b	917.39f	0.99	1250.76g	0.99	1216.35g	1.03f	0.99	1.41de
	Mollisol soil	2.70cd	771.35d	0.96	0.011a	784.45d	0.96	783.95d	0.99	865.19cd	0.92cd	0.99	1.01bc
	Fluvo-aquic soil	1.86ab	845.39e	0.97	0.0064a	860.33e	0.97	863.21e	0.99	1061.28f	0.88bc	0.99	1.34de
	Red soil	1.96abc	835.71e	0.99	0.0084a	845.43e	0.98	852.03e	0.99	995.41ed	0.83bc	0.99	1.16cd
$PE_{white\text{-}film}$	Water	1.92ab	828.06e	0.99	0.0067a	843.32e	0.99	905.82e	0.99	904.76de	1.00de	0.99	1.04bc
	Mollisol soil	1.76ab	656.39a	0.97	0.0071a	670.04a	0.98	536.93b	0.99	530.10a	1.01de	0.99	0.67a
	Fluvo-aquic soil	2.95de	714.21b	0.99	0.025b	718.64b	0.99	472.15a	0.97	726.36b	0.72a	0.99	0.83ab
	Red soil	1.60ab	742.42c	0.98	0.0060a	755.60c	0.98	671.24c	0.99	756.20bc	0.92cd	0.99	0.86ab
$PE_{pure\text{-}granule}$	Water	2.21bcd	1077.28i	0.99	0.0077a	1090.27h	0.99	1717.52i	0.99	1605.90i	1.08f	0.99	1.98g
	Mollisol soil	1.22a	948.89g	0.97	0.0026a	978.80g	0.98	1205.45fg	0.99	1341.30h	0.89bc	0.97	1.59ef
	Fluvo-aquic soil	1.27a	1051.05h	0.99	0.0027a	1077.57h	0.99	1151.75f	0.98	1412.50h	0.81b	0.98	1.73fg
	Red soil	1.14a	1038.16h	0.99	0.0022a	1070.66h	0.99	1485.31h	0.99	1591.79i	0.93cd	0.99	1.82fg

Note: Q_e is the sorption amount at sorption equilibrium respectively (mg kg^{-1}); k_1 and k_2 are pseudo-first-order and pseudo-second-order rate constants, respectively, and the units are (min^{-1}) and (kg mg^{-1} min^{-1}), respectively; K_d and K_F are the Linear and Freundlich constant (L mg^{-1}), respectively; $1/n$ is the sorption affinity constant; K_p is equilibrium partition ratio between DBP concentration in microplastics and background solution. The lower case letters in each column represent the significance of the difference at $p \leq 0.05$.

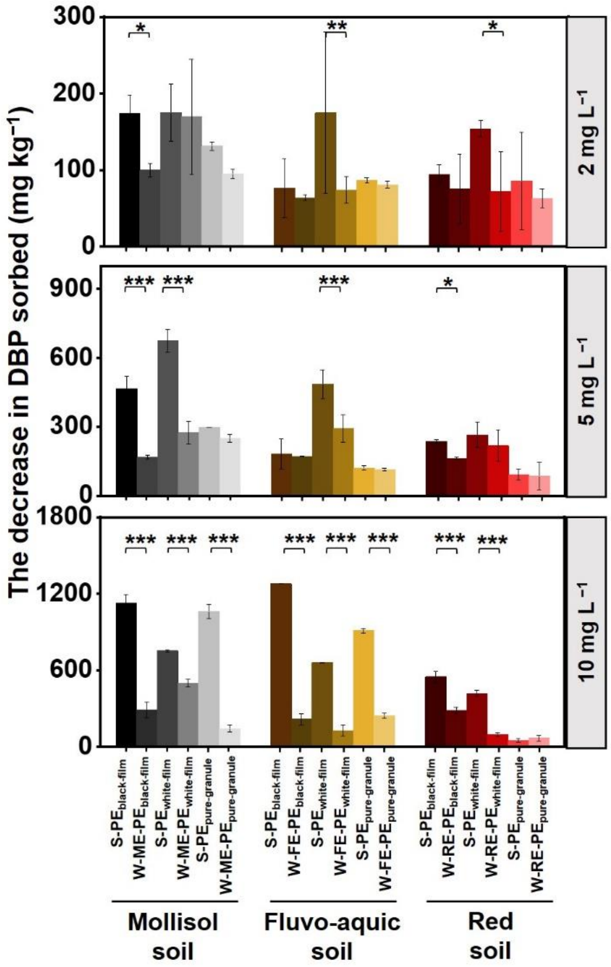

Figure S16. The decreased sorption capacity of dibutyl phthalate (DBP) to microplastics under different treatment conditions. PE$_{\text{black-film}}$: black polyethylene film microplastics; PE$_{\text{white-film}}$: white polyethylene film microplastics; PE$_{\text{pure-granule}}$: pure polyethylene microplastic granules; ： S-PE$_{\text{black-film}}$, S-PE$_{\text{white-film}}$, S-PE$_{\text{pure-granule}}$: PE$_{\text{black-film}}$, PE$_{\text{white-film}}$ and PE$_{\text{pure-granule}}$ in the soil solution; W-ME-PE$_{\text{black-film}}$, W-ME-PE$_{\text{white-film}}$, W-ME-PE$_{\text{pure-granule}}$: mollisol soil metabolites formed eco-corona on the surface of PE$_{\text{black-film}}$, PE$_{\text{white-film}}$ and PE$_{\text{pure-granule}}$ in water; W-FE-PE$_{\text{black-film}}$, W-FE-PE$_{\text{white-film}}$, W-FE-PE$_{\text{pure-granule}}$: fluvo-aquic soil metabolites formed eco-corona on the surface of PE$_{\text{black-film}}$, PE$_{\text{white-film}}$ and PE$_{\text{pure-granule}}$ in water; W-RE-PE$_{\text{black-film}}$, W-RE-PE$_{\text{white-film}}$, W-RE-PE$_{\text{pure-granule}}$: red soil metabolites formed eco-corona on the surface of PE$_{\text{black-film}}$, PE$_{\text{white-film}}$ and PE$_{\text{pure-granule}}$ in water (***$p \leq 0.001$, **$p \leq 0.01$, *$p \leq 0.05$).

Table S8. The chemical and physical properties of targeted metabolites.

	HMDB Superclass	Formula	CAS ID	Solubility	Structure
All-trans-Retinoic acid	Lipids and lipid-like molecules	$C_{20}H_{28}O_2$	302-79-4	< 1 g L^{-1}	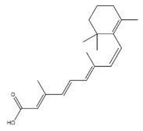
Thymine	Organoheterocyclic compounds	$C_5H_6N_2O_2$	65-71-4	3.82 g L^{-1}	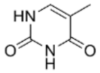
Aminocaproic acid	Lipids and lipid-like molecules	$C_6H_{13}NO_2$	60-32-2	505 g L^{-1}	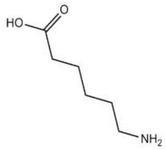
Betaine	Organic acids and derivatives	$C_5H_{11}NO_2$	107-43-7	611 g L^{-1}	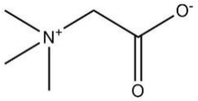
L-isoleucine	Organic acids and derivatives	$C_6H_{13}NO_2$	73-32-5	41.2 g L^{-1}	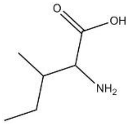
L-glutamic acid	Organic acids and derivatives	$C_5H_9NO_4$	56-86-0	7.5 g L^{-1}	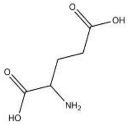
Phytosphingosine	Organic nitrogen compounds	$C_{18}H_{39}NO_3$	554-62-1	-	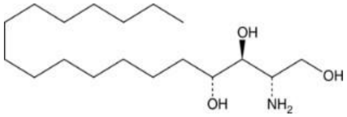
Deoxyadenosine	Nucleosides, nucleotides, and analogues	$C_{10}H_{13}N_5O_3$	958-09-8	≥ 8.37 g L^{-1}	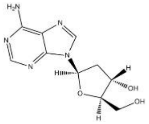
